# 2,5-Di-tert-butyl-2,5-diethylpyrrolidine-1-oxyls: Where Is a Reasonable Limit of Sterical Loading for Higher Resistance to Reduction?

**DOI:** 10.3390/molecules29030599

**Published:** 2024-01-25

**Authors:** Irina F. Zhurko, Sergey A. Dobrynin, Yurii I. Glazachev, Yuri V. Gatilov, Igor A. Kirilyuk

**Affiliations:** 1 N. N. Vorozhtsov Novosibirsk Institute of Organic Chemistry SB RAS, Lavrentiev Ave. 9, Novosibirsk 630090, Russia; zhurko@nioch.nsc.ru (I.F.Z.); dobrynin@nioch.nsc.ru (S.A.D.); gatilov@nioch.nsc.ru (Y.V.G.); 2 Voevodsky Institute of Chemical Kinetics and Combustion SB RAS, Institutskaya 3, Novosibirsk 630090, Russia; glaza@kinetics.nsc.ru

**Keywords:** three-component domino reaction, azomethine ylide, 1,3-dipolar cycloaddition, reduction-resistant nitroxide, EPR

## Abstract

The pyrrolidine nitroxides with four bulky alkyl substituents adjacent to the N–O∙ group demonstrate very high resistance to reduction with biogenic antioxidants and enzymatic systems. This makes them valuable molecular tools for studying the structure and functions of biomolecules directly in a living cell and for functional EPR and NMR tomography in vivo. The first example of highly strained pyrrolidine nitroxides with both ethyl and *tert*-butyl groups at each of the α-carbon atoms of the nitroxide moiety with *cis*-configuration of the *tert*-butyl groups was prepared using a three-component domino reaction of *tert*-leucine and 2,2-dimethylpentan-3-one with dimethyl fumarate with subsequent conversion of the resulting strained pyrrolidine into 1-pyrroline-1-oxide and addition of EtLi. The nitroxide has demonstrated unexpectedly fast reduction with ascorbate, the rate constant *k*_2_ = (2.0 ± 0.1) × 10^−3^ M^−1^s^−1^. This effect was explained by destabilization of the planar nitroxide moiety due to repulsion with the two neighboring *tert*-butyl groups *cis* to each other.

## 1. Introduction

Nitroxides are a broad family of organic free radicals which have been of constantly growing interest to researchers for many decades [1,2,3]. The relative simplicity of chemical modification of nitroxide structures resulted in the synthesis of countless numbers of derivatives, allowing for tuning of their chemical and physical properties and spectral parameters for specific applications in various fields of science and technology [4,5,6,7,8,9,10,11,12]. Rapid development of the chemistry of nitroxides is facilitating the progress in their applications. For example, the recent findings in synthesis of the nitroxides with enhanced stability to chemical reduction [13,14,15,16] opened up new opportunities for studying the structure and functions of biomolecules directly in a living cell [17,18,19,20] and for the development of new reagents for functional EPR and NMR tomography in vivo [21,22,23,24]. The higher stability of these so-called “sterically shielded” nitroxides resulted from introduction of four ethyl groups to the neighboring carbons of the nitroxide moiety instead of methyls, typical for a broad majority of conventionally used nitroxides. This effect has been carefully studied by various authors [25,26,27,28], and it was demonstrated that ring size and steric and electronic effects of the substituents play important roles. However, synthesis of highly sterically loaded structures remains a challenge. 

**Figure 1:**
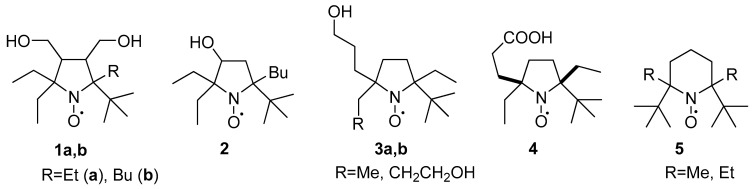
Structure of nitroxides **1**–**5**. **1a,b** [33,34]; **2** [33]; **3a,b** [36]; **4** [36]; **5** [37].

The three-component domino reaction of amino acids with ketones and activated alkenes is a powerful method of synthesis of various substituted pyrrolidines, including highly strained molecules [29,30,31]. This reaction was used to prepare highly strained, stable nitroxides with β-hydrogen [32]. In our studies of this reaction, we have developed a convenient procedure for the synthesis of strained pyrrolidines with three large alkyl substituents at positions 2 and 5, which can be used for preparation of various sterically shielded nitroxides of pyrrolidine series [16,33,34,35,36]. It should be noted that even highly strained 2-*tert*-butyl-substituted nitroxides **1a**,**b** (Figure 1) can be prepared with much higher overall yields than nitroxides **2**–**4** prepared via alternative methods [33,36]. For example, 2-(*tert*-butyl)-2-butyl-5,5-diethyl-3,4-bis(hydroxymethyl)pyrrolidin-1-oxyl (**1b**) was prepared from *tert*-leucine, 3-pentanone and dimethyl fumarate in four steps with the overall yield 36% [33]. These radicals showed very high stability to reduction. 

It is known that additional large splittings on the methylene hydrogens of the ethyl (*n*-alkyl) groups may be observed in the ESR spectra of pyrrolidine radicals with substituents in the 3 and 4 positions of the heterocycle [16,34,35]. These splittings were assigned to hyperfine coupling (*hfc*) with γ-hydrogen in the side chain and result from overlapping of the smaller lobe of the C–H orbital in the α-position of the side alkyl chain and the non-bonding orbital of the nitroxide group [38]. This overlapping is efficient when the ethyl group is in the pseudoaxial position of the pyrrolidine ring with the CH_2_–CH_3_ bond nearly parallel to the N–O axis. The introduction of a bulky group, such as *tert*-butyl, makes this conformation unfavorable and leads to the disappearance of the large *hfc* on the methylene hydrogen of the adjacent ethyl group, which makes the spectrum simpler.

Considering the above-described influence of the *tert*-butyl group on the properties of nitroxides, it can be assumed that a similar radical with two *tert*-butyl groups in positions 2 and 5 should be characterized by a higher resistance to reduction and a simple triplet EPR spectrum without large extra splittings. This work describes our efforts to synthesize such a radical and some of its properties and spectral features. To the best of our knowledge, the only communication on synthesis of a stable cyclic nitroxide with two *tert*-butyl groups at the α-carbons (**5**) was given at the conference [37], and this information was never published elsewhere.

## 2. Results and Discussion

**Scheme 1:**
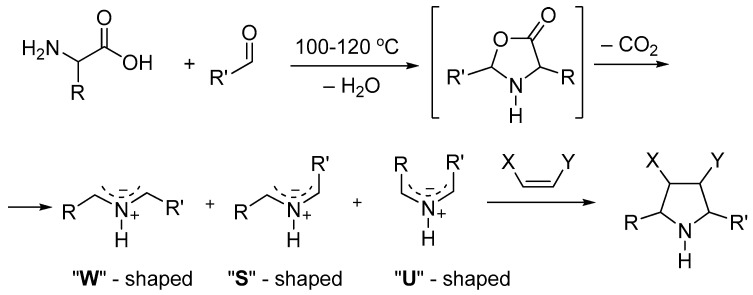
Formation of pyrrolidines in a three-component reaction of amino acids, carbonyl compounds and activated alkenes.

A reaction of amino acids with carbonyl compounds and activated alkenes is known to proceed via oxazolidin-5-one formation with subsequent release of carbon dioxide to give azomethine ylide (Scheme 1). 

**Scheme 2:**
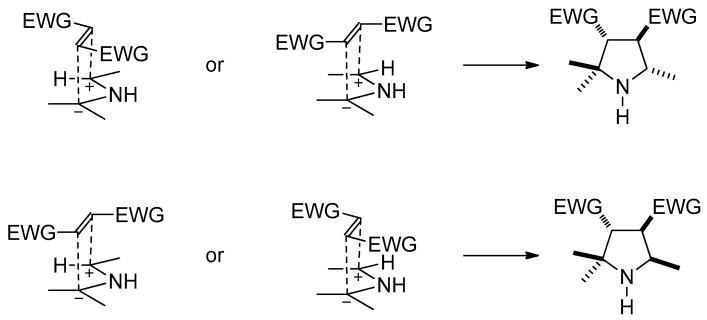
Formation of pyrrolidines in a three-component reaction of amino acids, carbonyl compounds and activated alkenes.

1,3-Dipolar cycloaddition of alkenes to azomethine ylides can give a set of isomeric pyrrolidines, resulting from the reaction of the “W”-, “S”- or “U”-shaped ylide with the alkene approaching from the upper or lower side [39,40]. We have previously observed formation of two diastereoisomeric pyrrolidines in the reactions with symmetric ketones [33,34], which is the maximal number of isomers that can form upon the reaction of two possible configurations of an azomethine ylide with a symmetric trans-alkene (Scheme 2). Introduction of an asymmetric ketone into this reaction was expected to give four isomers due to addition of a new asymmetric center in position 5 of the heterocycle. 

**Scheme 3:**
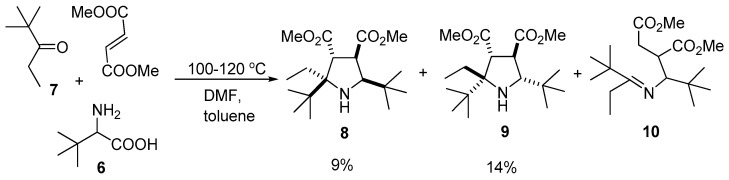
The three-component reaction of 2,2-dimethylpentan-3-one (**6**), *tert*-leucine (**7**) and dimethyl fumarate.

According to our previous experience [34], the yield of the target pyrrolidine in the three-component domino reaction of amino acids with ketones and activated alkenes may be sensitive to steric hindrance (volume of the substituents in the reagents); however, the resulting pyrrolidines can be easily separated via extraction with aqueous acidic solution even if the yield is low. After heating *tert*-leucine (**6**) and 2,2-dimethylpentan-3-one (**7**) with dimethyl fumarate in a DMF–toluene mixture in Dean–Stark apparatus for 80 h, a mixture of basic compounds was separated using extraction with an acid, and pure compounds **8**, **9** and **10** were isolated using column chromatography (Scheme 3). 

The spot of **10** on TLC did not give the characteristic staining with Dragendorff’s reagent, but HRMS of **8** and **10** and the element analysis data for **9** corresponded to the same formula, C_18_H_33_NO_4_. The spectra of **8** and **9** showed much similarity to those of previously described diastereomeric pyrrolidines [16,33]. In the IR spectra of the **8** and **9** bands, N–H vibrations at 3500–3300 cm^−1^ were very weak, and C=O vibrations of the ester groups were represented by a single strong band at 1720–1740 cm^−1^ (see Section 3 and Figures S50 and S51, cf. [33]). Similarly to that described for mono-*tert*-butyl pyrrolidines [33], one of the isomers (**9**) showed well-resolved signals of methine protons in the NMR ^1^H while they were overlapping in the spectrum of **8**. Addition of CF_3_COOH allowed the spectrum to be recorded with resolved signals of these protons (Figures S3 and S5). The NMR ^13^C spectra of **8** and **9** demonstrated much difference, but were both consistent with the pyrrolidine structure (see Section 3 and Figures S4, S6 and S7). 

To determine the relative configuration of the asymmetric centers in **8** and **9**, ^1^H–^1^H NOESY correlation spectra were recorded (Figures S36 and S37, Appendix A), which showed that the *tert*-butyl groups are *cis* to each other in both isomers **8** and **9**. Obviously, formation of these isomers results from addition of the dipolarophil from both sides of the “W”-shaped (with respect to *tert*-butyls) azomethine ylide plane because a “U” shape is hardly possible for the ylide with such bulky substituents.

In contrast, the IR spectrum of **10** showed a band at 1647 cm^−1^, typical for C=N bond vibrations (Figure S52). The ^1^H NMR spectrum of **10** (Figure S9) showed the signals of a four-spin system of two methylene and two methine protons, along with the signals of two *tert*-butyl groups, two methoxy groups and an ethyl group. The NMR ^13^C spectrum (Figure S10) showed three signals between 173 and 178 ppm, a signal of a methylene carbon at 32.9 ppm and only two signals of methine carbons at 42.3 and 65.5 ppm. These data support assignment of the acyclic structure to **10**. Formation of this compound could result from Michael addition of an azomethine ylide to dimethyl fumarate; for examples of similar reactions, see [41] and reference [2] therein. 

**Scheme 4:**
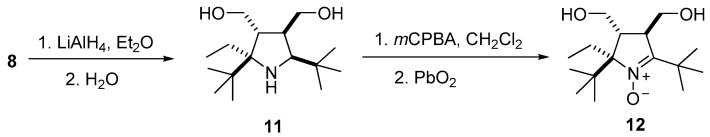
Synthesis of **12**.

The subsequent transformations of **8** were carried out in analogy with our previous syntheses of the sterically shielded nitroxides [16,33,34]. The ester groups were reduced with LiAlH_4_ to give **11** (Scheme 4). The structure of the new compound was assigned on the basis of IR, NMR ^1^H and ^13^C spectra and confirmed by the elemental analyses (see Section 3 and Figures S11, S12 and S53). Surprisingly, no trace of conversion was observed upon treatment of **11** with hydrogen peroxide in the presence of sodium tungstate. The desired oxidation to nitrone was achieved using *m*-chloroperbenzoic acid (*m*CPBA). To avoid over-oxidation to oxoammonium cations with possible affection of the hydroxymethyl groups (cf. [42,43]), the amines were treated with ca. one equivalent of the oxidant. Analysis of the reaction mixtures using TLC showed the spots that give blue staining upon treatment with 10% solution of phosphomolibdic acid in ethanol, typical for hydroxylamines [16]. To complete the oxidation, lead dioxide was added to the reaction mixtures after *m*CPBA was consumed. The resulting nitrone **12** was isolated with the yield 65%. 

The structure of **12** was proved using NMR ^1^H and ^13^C spectra and experiments examining correlations ^1^H–^13^C HMBC and ^1^H–^1^H NOESY and supported by elemental analysis data (see Section 3, Figures S13, S14, S38 and S39 and Appendix A).

**Scheme 5:**
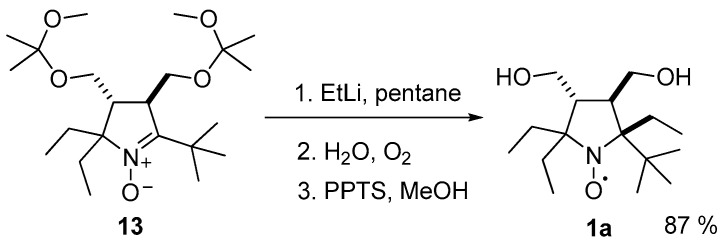
Addition of EtLi to the nitrone **13**.

Earlier, the nitroxides **3a**,**b** were prepared with 50–70% yield via addition of ethyl lithium to corresponding 2-*tert*-butyl-1-pyrroline-1-oxides [36]. This reagent also rapidly reacted with the nitrone **13** to give the nitroxide **1a** with 87% yield (Scheme 5). An alternative synthesis of this nitroxide from the same nitrone via addition of ethynylmagnesium bromide with subsequent hydrogenation gave 43%, and it took 72 days for the reaction to complete [34]. 

**Scheme 6:**
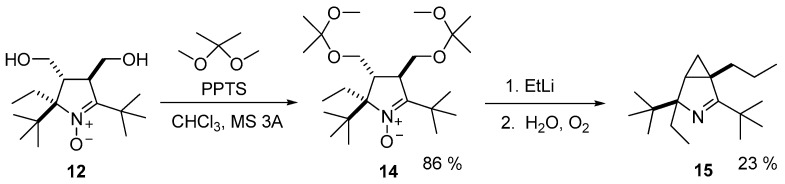
Synthesis of **14** and its reaction with EtLi.

Protected 1-pyrroline-*N*-oxide **14** was prepared from **12** using a previously described procedure [34] and isolated using column chromatography (Scheme 6), and the structure of this nitrone was confirmed by IR, NMR ^1^H and ^13^C spectra and the elemental analysis (Figures S15, S16 and S56). Contrary to our expectations, the reaction of **14** with an excess of ethyl lithium did not lead to the formation of a nitroxide radical. A single diamagnetic compound was isolated from the complex mixture of the reaction products. The high-resolution mass spectrum of the new compound contained molecular ion [M^+^] = 263.2612, which corresponded to the formula C_18_H_33_N, i.e., all oxygen atoms were lost. In addition to the signals of *tert*-butyl and *n*-alkyl groups, the NMR spectrum ^1^H contained multiplets in the high field (0.12 and 0.77 ppm), apparently indicating formation of a cyclopropane ring (Figure S19). The detailed analysis of the spectrum with line shape simulations (Figure S48) allowed us to assign the structure **15** to the compound. Final assignment of the relative configuration of the asymmetric centers was performed on the basis of ^1^H–^1^H COSY, ^1^H–^13^C HSQC, ^1^H–^13^C HMBC and ^1^H–^1^H NOESY correlations (Figures S40–S43 and Appendix A). 

**Scheme 7:**
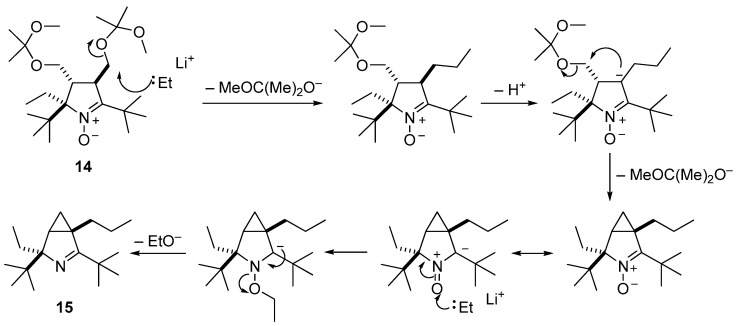
Possible scheme of formation of **15**.

Compound **15** could be formed according to Scheme 7. Presumably the nitrone carbon in **14** is too hindered for the addition of EtLi to occur. As a result, metalation proceeds due to the relatively high acidity of β-hydrogen of the nitrone group. Subsequent nucleophilic substitution leads to cyclopropane ring formation. Earlier, we observed deoxygenation of the nitrone group and nucleophilic substitution of the OTMS group in the reaction of TMS-protected 2-tert-butyl-1-pyrroline-1-oxide with butyllithium [33]. 

**Scheme 8:**
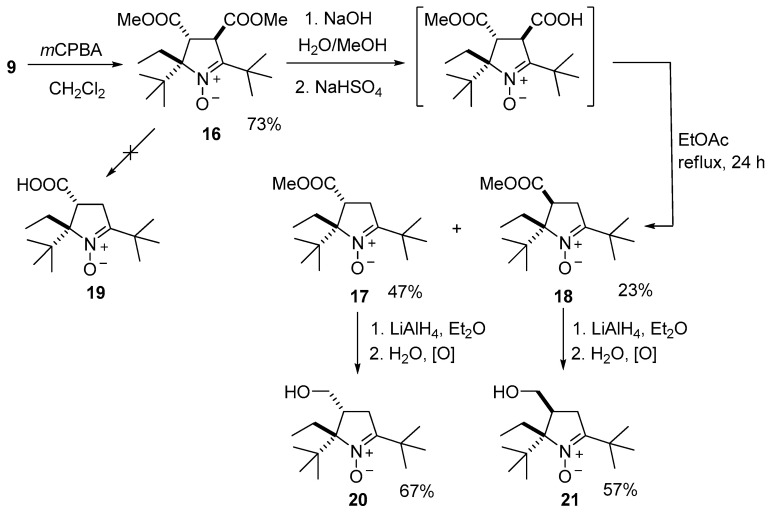
Synthesis of **16**, alkaline hydrolysis and decarboxylation. Synthesis of **20** and **21**.

Despite the synthesis of 2,5-di-tert-butylpyrrolidine nitroxide from **12** being unsuccessful, the pyrrolidines **8** and **9** can be converted into less hindered 3-unsubstituted nitrones in analogy to the previously described procedure [35]. The major isomer **9** was subjected to oxidation with *m*CPBA in dichloromethane to give **16** (Scheme 8). The latter was subjected to alkaline hydrolysis in aqueous methanol at ambient temperature. The starting compound reacted completely within 48 h. After acidification of the reaction mixture, the products were extracted and heated to reflux in EtOAc in analogy to the previously described procedure [35]. Surprisingly, the two major products (total yield 70%) did not demonstrate an acidic nature. The compounds were isolated using column chromatography with the yields 47 and 23%. NMR, IR and HRMS spectra of both compounds unambiguously corresponded to the structure of methyl esters **17** and **18** of the desired carboxylic acid **19** (Figures S23–S26, S59 and S60). Presumably, the low steric accessibility of the carbon of the ester group at position 4 of the heterocycle prevented its alkaline hydrolysis; however, in an alkaline solution, an inversion of the configuration of the neighboring asymmetric center could occur, which led to the formation of two diastereomers.

**Figure 2:**
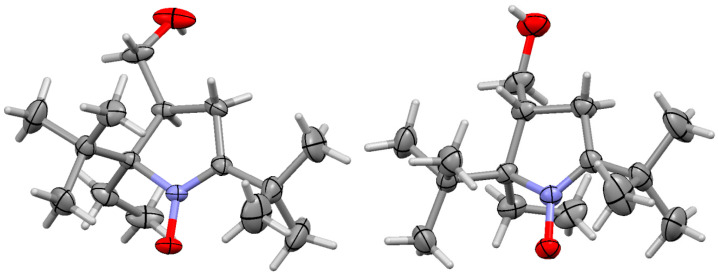
The structure of **20** (**left**) and **21** (**right**) according to single-crystal X-ray diffraction data.

Treatment of **17** and **18** with LiAlH_4_ leads to reduction of both the ester group to hydroxymethyl one and the nitrone group to hydroxylamine. Subsequent oxidation with manganese dioxide recovered the nitrone group to give **20** and **21**. The data of a single crystal X-ray analysis of these compounds (Figure 2, Figures S66 and S67) showed the relative configuration of the asymmetric centers and confirmed the structure of **17**, **18**, **20** and **21**.

**Scheme 9:**
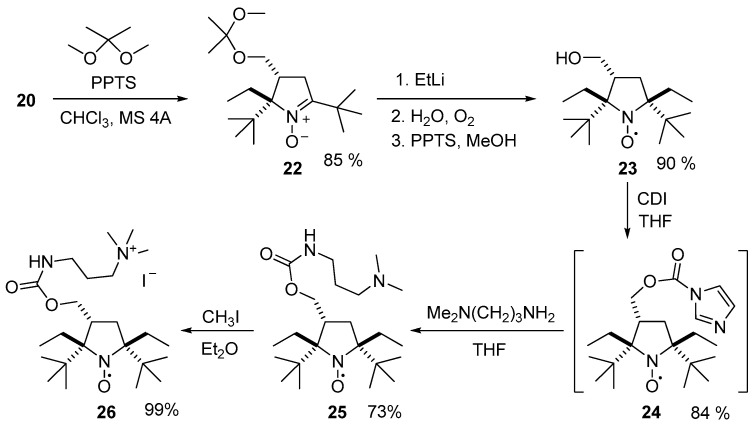
Synthesis of **23**.

The minor isomer **21** was prone to rapid tarring when stored at ambient temperature in aerobic conditions. For this reason, the major isomer **20** was used in subsequent syntheses. After protection of the hydroxyl group, the resulting nitrone **22** was treated with a 2.5-fold excess of EtLi, affording the nitroxide **23** with 90% yield (77% from **20**) (Scheme 9). The resulting nitroxide was isolated as an orange oil. The samples of **23** showed some evidence of slow decomposition at 25 °C. We previously reported on the thermal instability of some 2-*tert*-butyl-substituted nitroxides [33,44]. The structure of the nitroxide was confirmed with the NMR spectra recorded after reduction with Zn in the presence of trifluoroacetic acid in methanol-D_4_. To avoid thermal decomposition of the radical, the mixture was first stirred at −15 °C for 5 min and then heated to reflux, cf. [35]. The NMR ^1^H spectrum showed singlets of two different *tert*-butyl groups, well-resolved multiplets of two different ethyl groups and signals of an OCH_2_–CH–CH_2_ system (Figure S31). The data of NMR ^13^C, IR (Figures S32 and S63) and HRMS spectra and elemental analysis data were in agreement with the assigned structure. 

Bulky alkyl substituents make **23** insoluble in water, so it was converted into hydrophilic derivative **26** to carry out reduction rate and EPR spectra parameter measurements in conditions similar to previous measurements [16,35,36,45] (Scheme 9). The structure of **26** was confirmed by IR (Figure S65), HRMS TOF (ESI) and NMR ^1^H and ^13^C spectra recorded after the reduction with Zn/CF_3_COOH as described above (Figures S33, S34 and S49). The relative configuration of the asymmetric centers was determined using ^1^H–^1^H COSY, ^1^H–^13^C HSQC, ^1^H–^13^C HMBC and ^1^H–^1^H NOESY correlations (Figures S44–S47 and Appendix A). Analysis of the correlations showed that the synthesis afforded the nitroxides with cis-configuration of the *tert*-butyl groups. Formation of this isomer results from the addition of EtLi from the side opposite to the position of the bulky *tert*-butyl group.

In agreement with our expectations, the EPR spectra of **26** showed broadened triplet with peak-to-peak linewidths, H_p-p_ = 0.226 mT. This spectrum resembles the spectra of the reduction-resistant nitroxides **3a**,**b** and **4**. Thus, introduction of two *tert*-butyl groups into positions 2 and 5 allowed us to remove the large additional *hfc* with γ-hydrogens. 

Analysis of the kinetics of reduction of nitroxides **1a** and **26** with ascorbate in the presence of glutathione gave second-order rate constants (7.0 ± 2) × 10^−5^ M^−1^s^−1^ and (2.0 ± 0.1) × 10^−3^ M^−1^s^−1^, correspondingly. While the value of the rate constant for **1a** was in line with our previous data for highly strained nitroxides [33,36], the rate constant for **26** was even higher than those for 3-monosubstituted 2,2,5,5-tetraethylpyrrolidin-1-oxyls [35]. This result seems paradoxical. However, we earlier noticed that the effect of bulky substituents adjacent to the nitroxide group is associated with relative stabilization or destabilization of a nitroxide and corresponding hydroxylamine [26]. Obviously, the repulsion of the oxygen atom with the two neighboring *tert*-butyl groups makes the planar geometry of the nitroxide group unfavorable, and reduction to pyramidal hydroxylamine makes the molecule less distorted. This example clearly demonstrates that the effect of bulky substituents is not limited to a decrease in the accessibility of the nitroxide moiety, and distortions and tensions produced by bulky substituents may play an important role for nitroxide stability to reduction. This effect should be taken into account in the molecular design of reduction-resistant nitroxides.

## 3. Materials and Methods

### 3.1. General Information

The IR spectra were recorded on a Bruker Vector 22 FT-IR spectrometer (Bruker, Billerica, MA, USA) in KBr pellets (1:150 ratio) or in neat samples (Figures S50–S65) and are reported in wave numbers (cm^−1^). ^1^H NMR spectra were recorded on a Bruker AV 400 (400.134 MHz), DRX 500 (500.130 MHz) and AV 600 (600.300 MHz) spectrometers (Bruker, Billerica, MA, USA). ^13^C NMR spectrum was recorded on a Bruker AV 400 (100.033 MHz), DRX 500 (125.032 MHz) and AV 600 (150.075 MHz) (Figures S1–S49). All the NMR spectra were acquired for 5–10% solutions in CDCl_3_, (CD_3_)_2_CO or CD_3_OD at 300 K using the signal of the solvent as a standard. To confirm the structure of stable nitroxides, NMR spectra were recorded of the solutions of corresponding amines prepared via reduction of the nitroxide samples in analogy to the previously described method [35]. To avoid thermal decomposition of the radical, the solution of a nitroxide (20–40 mg) in CD_3_OD (0.5 mL) was cooled to −15 °C, and trifluoroacetic acid (0.1 mL) was added dropwise within 5 min, then the mixture was heated to reflux for 5 min and filtered into an NMR tube, cf. [35]. HRMS analyses were performed using a High-Resolution Mass Spectrometer DFS (Thermo Electron, Waltham, MA, USA) and Bruker micrOTOF-Q (Bruker, Billerica, MA, USA).

Reactions were monitored by TLC on precoated ALUGRAM Xtra SIL G/UV254 TLC sheets (Macherey-Nagel GmbH & Co. KG, Düren, Germany) using UV light 254 nm, 1% aqueous permanganate, 10% solution of phosphomolybdic acid in ethanol and Dragendorff’s reagent as visualizing agents. Kieselgel 60 (Macherey-Nagel GmbH & Co. KG) was utilized as an adsorbent for column chromatography. 

The X-ray diffraction experiments for crystals of **20** and **21** were carried out at 296(2) K on a Bruker KAPPA APEX II diffractometer (graphite-monochromated Mo Kα radiation). Reflection intensities were corrected for absorption by the SADABS program. The structures were solved by direct methods using the SHELXT 2014/5 program [46] and refined by anisotropic (isotropic for all H atoms) with full-matrix least-squares method against the F2 of all reflections by SHELXL2018/3 [46]. The positions of the hydrogen were calculated geometrically and refined in a riding model. Crystallographic data for **20** and **21** have been deposited at the Cambridge Crystallographic Data Centre as supplementary publication no. CCDC 2312569, CCDC 2312570. A copy of the data can be obtained free of charge, on application to CCDC, 12 Union Road, Cambridge CB21EZ, UK (fax: +44 122 3336033 or e-mail: deposit@ccdc.cam.ac.uk; internet: www.ccdc.cam.ac.uk, accessed on 1 January 2020).

### 3.2. Synthesis

#### 3.2.1. Procedure for 2,2-Dimethylpentan-3-one (**7**)

**Scheme 10:**
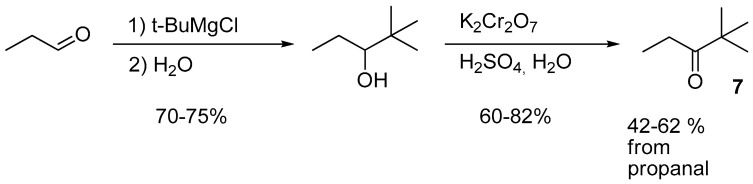
Synthesis of 2,2-dimethylpentan-3-one (**7**).

Procedures from the literature [47,48,49] did not give a satisfactory yield of this ketone; therefore, this reagent was prepared in a two-step procedure according to Scheme 10. 

A solution of propionaldehyde (20 g, 0.356 mol) in dry diethyl ether (70 mL) was added dropwise within 3 h to a solution of *t*-BuMgCl prepared from 43 g (0.463 mol) of *tert*-butylchloride and 11 g of magnesium foil in 500 mL of dry diethyl ether upon active stirring. The reaction mixture was then left overnight at room temperature and carefully quenched with water until viscous inorganic sludge formation. The organic solution was separated by decantation, and the sludge was washed repeatedly with portions of diethyl ether. The combined extract was dried with sodium carbonate and concentrated under reduced pressure without heating. The resulting compound contained up to 8% Et_2_O and was then used without purification. Yield of 2,2-dimethylpentan-3-ol was 31.3 g (72%). ^1^H NMR spectra (Figure S1) corresponded to the literature data [50].

The mixture of sodium dichromate (40 g, 0.15 mol), water (500 mL) and sulfuric acid (30 mL) was cooled to 0 °C and added dropwise within 3.5 h to the stirred cold (0–+5 °C) solution of 2,2-dimethylpentan-3-ol (31.3 g, 0.245 mol) in diethyl ether (30 mL). The temperature was kept below 5 °C. Then, the mixture was allowed to warm to 20 °C, and the organic phase was separated. The aqueous solution was saturated with sodium sulfate and extracted with diethyl ether (5 × 50 mL). The combined ether extract was washed with saturated aqueous NaCl solution and dried with sodium carbonate. The ether was distilled off, and the residue was distilled at normal pressure to give 2,2-dimethylpentan-3-one (**7**), 25 g (81%); ^1^H NMR spectra of 2,2-dimethylpentan-3-one (Figure S2) corresponded to the literature [51].

#### 3.2.2. Condensation of 2,2-Dimethylpentan-3-one (**7**), 2-Amino-3,3-dimethylbutanoic Acid (**6**) and Dimethyl fumarate

A mixture of **6** (1.6 g, 11.8 mmol), dimethyl fumarate (1.7 g, 11.8 mmol), **7** (2.7 g, 23.7 mmol), DMF (12 mL) and toluene (12 mL) was placed into a Dean–Stark apparatus and stirred under reflux for 8 days. The solvent was removed in vacuum, and the residue was dissolved in ethyl acetate (50 mL). The solution was washed with aqueous sodium bicarbonate (30 mL × 4 times) and extracted with 5% (1 M) sulfuric acid (30 mL × 10 times) until all the products were extracted according to TLC on silica gel, eluent ethyl acetate–hexane 1:20 mixture, *R_f_* = 0.8 for **8**, 0.7 for **9**, 0,6 (for **10**). The combined acidic extracts were basified with Na_2_CO_3_ to pH 8 and extracted with ethyl acetate (30 mL × 3 times). The extract was dried with Na_2_CO_3_, and the solvent was removed in vacuum to give 1.75 g of a crude products mixture. The compounds **8**, **9** and **10** were isolated via column chromatography on silica gel using ethyl acetate–hexane mixture 1:20 as eluent.

*(2R(S),3R(S),4R(S),5S(R))-2,5-Di-tert-butyl-3,4-bis(methoxycarbonyl)-2-ethylpyrrolidine (***8***).* Yield 0.365 g (9%), colorless oil; HRMS (EI/DFS) m/z [M]^+^ calcd for C_18_H_33_NO_4_ 327.2404, found 327.2406; IR (neat) ν_max_: 1730 (C=O) cm^−1^; ^1^H NMR (500 MHz; CDCl_3_, δ): 0.89 (t, J_t_ = 7.5 Hz, 3H), 0.92 (s, 9H), 1.04 (s, 9H), 1.58 (dq, J_d_ = 15.2 Hz, J_q_ = 7.5 Hz, 1H), 1.66 (dq, J_d_ = 15.2 Hz, J_q_ = 7.5 Hz, 1H), 1.75–1.82 (br, 1H), 3.06 (d, J_d_ = 8.1 Hz, 1H), 3.44 (d, J_d_ = 6.4 Hz, 1H), 3.45 (dd, J_d1_ = 8.1 Hz, J_d2_ = 6.4 Hz, 1H), 3.60 (s, 3H), 3.61 (s, 3H); ^13^C NMR (125 MHz; CDCl_3_, δ): 10.1, 23.4, 27.2, 27.5, 33.7, 38.6, 50.1, 51.3, 51.4, 55.0, 68.8, 70.8, 174.0, 175.4; ^1^H NMR (400 MHz; (CD_3_)_2_CO, CF_3_COOH, δ): 1.15 (t, J_t_ = 7.5 Hz, 3H), 1.17 (s, 9H), 1.27 (s, 9H), 2.10 (dq, J_d_ = 15.6 Hz, J_q_ = 7.5 Hz, 1H), 2.33 (dq, J_d_ = 15.6 Hz, J_q_ = 7.5 Hz, 1H), 3.74 (s, 3H), 3.76 (s, 3H), 3.88 (d, J_d_ = 3.8 Hz, 1H), 3.93 (dd, J_d1_ = 7.5 Hz, J_d2_ = 3.8 Hz, 1H), 4.12 (d, J_d_ = 7.5 Hz, 1H); ^13^C NMR (100 MHz; (CD_3_)_2_CO, CF_3_COOH, δ): 10.2, 24.7, 27.0, 27.4, 33.7, 40.1, 47.5, 52.6, 53, 30, 53.31, 70.8, 78.5, 170.9, 173.9.

*(2S(R),3R(S),4R(S),5R(S))-2,5-Di-tert-butyl-3,4-bis(methoxycarbonyl)-2-ethylpyrrolidine (***9***).* Yield 0.530 g (14%), colorless oil; HRMS (EI/DFS) m/z [M-15]^+^ calcd for C_17_H_30_NO_4_ 312.2169, found 312.2170; Elemental analysis: found: C, 66.16; H, 10.06; N, 4.37; calcd for C_18_H_33_NO_4_: C, 66.02; H, 10.16; N, 4.28. IR (neat) ν_max_: 3379 (NH), 1738 (C=O) cm^−1^; ^1^H NMR (500 MHz; CDCl_3_, δ): 0.87 (s, 18H), 1.03 (t, J_t_ = 7.4 Hz, 3H), 1.15–1.21 (br, 1H), 1.69 (dq, J_d_ = 14.3 Hz, J_q_ = 7.4 Hz, 1H), 1.96 (dq, J_d_ = 14.3 Hz, J_q_ = 7.4 Hz, 1H), 3.02 (d, J_d_ = 10.2 Hz, 1H), 3.13 (dd, J_d1_ = 12.3 Hz, J_d2_ = 10.2 Hz, 1H), 3.40 (d, J_d_ = 12.3 Hz, 1H), 3.60 (s, 3H), 3.61 (s, 3H); ^13^C NMR (125 MHz; CDCl_3_, δ): 8.1, 26.2, 26.3, 28.2, 33.9, 40.0, 47.5, 51.2, 51.6, 53.5, 68.0, 70.3, 172.9, 175.1.

*Dimethyl 2-(1-(2,2-dimethylpentan-3-ylideneamino)-2,2-dimethylpropyl)succinate (***10***)*. Yield 0.23 g (6%), colorless oil; IR (neat) HRMS (EI/DFS) m/z [M]^+^ calcd for C_18_H_33_NO_4_ 327.2404, found 327.2405; ν_max_: 1740 (C=O), 1647 (C=N) cm^−1^; ^1^H NMR (500 MHz; CDCl_3_, δ): 0.86 (s, 9H), 0.96 (t, J_t_ = 7.8 Hz, 3H), 1.08 (s, 9H), 2.10 (dq, J_d_ = 13.7 Hz, J_q_ = 7.8 Hz, 1H), 2.19 (dq, J_d_ = 13.7 Hz, J_q_ = 7.8 Hz, 1H), 2.46 (dd, J_d1_ = 17.2 Hz, J_d2_ = 10.5 Hz, 1H), 2.62 (dd, J_d1_ = 17.2 Hz, J_d2_ = 3.8 Hz, 1H), 3.27 (ddd, J_d1_ = 10.5 Hz, J_d2_ = 3.8 Hz, J_d2_ = 3.9 Hz, 1H), 3.50 (d, J_d_ = 3.9 Hz, 1H), 3.60 (s, 3H), 3.62 (s, 3H); ^13^C NMR (125 MHz; CDCl_3_, δ): 12.6, 19.9, 26.6, 28.3, 32.9, 42.3, 51.4, 51.7, 65.5, 173.3, 175.8, 178.0.

#### 3.2.3. Procedure for the Synthesis of (2R(S),3R(S),4R(S),5S(R))-2,5-Di-tert-butyl-3,4-bis(hydroxymethyl)-2-ethylpyrrolidine (**11**)

A solution of the diester **8** (5.7 mmol) in 20 mL of dry diethyl ether was added dropwise to a stirred suspension of LiAlH_4_ (0.87 g, 22.9 mmol) in 25 mL of dry diethyl ether. After addition was complete, the reaction mixture was stirred at reflux for 1 h, then cooled in an ice bath and carefully quenched with 5% aqueous sodium hydroxide (2 mL) and then water (2 mL). The organic solution was separated via decantation, the precipitate was washed with diethyl ether (10 mL × 3 times). The combined extract was dried with sodium carbonate and evaporated under reduced pressure to give **11**. Yield 100%, colorless crystalline solid, m.p. 83–85 °C (hexane). Elemental analysis: found: C, 71.36; H, 12.32; N, 5.20; calcd for C_16_H_33_NO_2_: C, 70.80; H, 12.25; N, 5.16. IR (KBr) ν_max_: 3475, 3334 (OH, NH) cm^−1^; HRMS (EI/DFS) m/z [M-1]^+^ calcd for C_16_H_32_O_2_N 270.2428, found 270.2426; ^1^H NMR (500 MHz; CDCl_3_, δ): 0.80 (t, J_t_ = 7.4 Hz, 3H), 0.90 (s, 9H), 0.92 (s, 9H), 1.35–1.45 (br, 1H), 1.50 (dq, J_d_ = 14.9 Hz, J_q_ = 7.4 Hz, 1H), 1.57 (dq, J_d_ = 14.9 Hz, J_q_ = 7.4 Hz, 1H), 1.93 (dddd, J_d1_ = 11.5 Hz, J_d2_ = 7.1 Hz, J_d3_ = 3.8 Hz, J_d4_ = 2.7 Hz, 1H), 2.09 (ddd, J_d1_ = 10.7 Hz, J_d2_ = 3.3 Hz, J_d3_ = 2.7 Hz, 1H), 2.80 (d, J_d_ = 7.1 Hz, 1H), 3.34 (dd, J_d1_ = 11.5 Hz, J_d2_ = 8.7 Hz, 1H), 3.37 (dd, J_d1_ = 10.7 Hz, J_d2_ = 9.5 Hz, 1H), 3.64 (dd, J_d1_ = 9.5 Hz, J_d2_ = 3.3 Hz, 1H), 3.87 (dd, J_d1_ = 8.7 Hz, J_d2_ = 3.8 Hz, 1H); 4.18–4.28 (br, 1H), 4.30–4.40 (br, 1H); ^13^C NMR (125 MHz; CDCl_3_, δ): 9.6, 20.3, 26.5, 27.7, 33.3, 38.8, 50.0, 51.7, 62.4, 64.7, 65.3, 66.7.

#### 3.2.4. Procedure for the Synthesis of (2R(S),3R(S),4R(S))-2,5-Di-tert-butyl-2-ethyl-3,4-bis(hydroxymethyl)-3,4-dihydro-2H-pyrrole 1-oxide (**12**)

A solution of *m*CPBA in CH_2_Cl_2_ (0.67 g, 3.9 mmol) was cooled to −15 °C (10 mL) and added quickly to a solution of **11** in CH_2_Cl_2_ (0.84 g, 3.1 mmol) cooled to −15 °C (10 mL). The reaction mixture was allowed to stand at room temperature for 12 h and then stirred with PbO_2_ (0.5 g, 2.1 mmol) for 1 h. The precipitate was filtered off, and the filtrate was evaporated in vacuum. The crude product was purified via column chromatography on silica gel using ethyl acetate–hexane 1:1 mixture as eluent and recrystallized from ethyl acetate to give **12** in 0.57 g (65%) yield. Colorless crystalline solid, m.p. 157–159 °C (ethyl acetate). Elemental analysis: found: C, 67.15; H, 10.99; N, 5.03; calcd for C_16_H_31_NO_3_: C, 67.33; H, 10.95; N, 4.91; IR (KBr) ν_max_: 3402 (OH), 1566 (C=N) cm^−1^; ^1^H NMR (400 MHz, CDCl_3_, δ): 0.69 (t, J_t_ = 7.2 Hz, 3H), 1.05 (s, 9H), 1.28 (s, 9H), 1.35 (dq, J_d_ = 14.8 Hz, J_q_ = 7.2 Hz, 1H), 2.19 (dq, J_d_ = 14.8 Hz, J_q_ = 7.2 Hz, 1H), 2.32 (ddd, J_d1_ = 9.6 Hz, J_d2_ = 5.6 Hz, J_d3_ = 3.9 Hz, 1H), 2.81 (ddd, J_d1_ = 9.4 Hz, J_d2_ = 5.6 Hz, J_d3_ = 2.9 Hz, 1), 3.28 (dd, J_d1_ = 10.0 Hz, J_d2_ = 9.4 Hz, 1H), 3.60–3.72 (m, 2H), 4.17 (d, J_d_ = 10.0 Hz, 1H), 5.12–5.23 (br, 1H), 5.31–5.43 (br, 1H); ^13^C NMR (100 MHz, CDCl_3_, δ): 9.6, 21.1, 25.7, 26.3, 34.5, 37.7, 44.8, 51.4, 62.5, 64.6, 85.0, 152.0.

#### 3.2.5. Procedure for the Synthesis of (2S(R),3R(S),4R(S))-2,5-Di-tert-butyl-2-ethyl-3,4-bis(methoxycarbonyl)-3,4-dihydro-2H-pyrrole 1-oxide (**16**)

The compound **16** was prepared from **9** similarly to the above procedure. The resulting compound **16** was isolated via column chromatography on silica gel using ethyl acetate–hexane 1:6 mixture as eluent. Yield from 3.27 g (10 mmol) of **9**—2.50 g (7.33 mmol, 73%). Colorless oil; Elemental analysis: found: C, 63.62; H, 9.50; N, 3.85; calcd for C_18_H_31_NO_5_: C, 63.32; H, 9.15; N, 4.10; IR (neat) ν_max_: 1743 (C=O), 1550 (C=N) cm^−1^; ^1^H NMR (500 MHz; CDCl_3_, δ): 0.95 (t, J_t_ = 7.2 Hz, 3H), 0.97 (s, 9H), 1.24 (s, 9H), 1.71 (dq, J_d_ = 14.2 Hz, J_q_ = 7.2 Hz, 1H), 2.33 (dq, J_d_ = 14.2 Hz, J_q_ = 7.2 Hz, 1H), 3.47 (d, J_d_ = 10.0 Hz, 1H), 3.66 (s, 3H), 3.69 (s, 3H), 4.23 (d, J_d_ = 10.0 Hz, 1H); ^13^C NMR (125 MHz; CDCl_3_, δ):7.9, 23.0, 25.0, 26.9, 34.4, 39.3, 46.8, 49.9, 52.0, 52.6, 86.4, 147.1, 170.5, 173.4.

#### 3.2.6. Procedure for the Synthesis of (2R(S),3R(S),4R(S))-2,5-di-tert-butyl-2-ethyl-3,4-bis(((2-methoxypropan-2-yl)oxy)methyl)-3,4-dihydro-2H-pyrrole 1-oxide (**14**)

The compound **14** was prepared according to the literature procedure for protecting hydroxy groups with 2,2-dimethoxypropane [34]. The crude product was purified via column chromatography on silica gel using ethyl acetate–hexane 1:4 mixture as eluent. The yield of **14** from 0.5 g (1.75 mmol) was 0.65 g (86%). Colorless crystalline solid, m.p. 80–84 °C (ethyl acetate–hexane). HRMS (EI/DFS) m/z [M-73]^+^ calcd for C_20_H_38_O_4_N 356.2795, found 356.2793, [M-72]^+^ calcd for C_20_H_39_O_4_N 357.2874, found 357.2872; Elemental analysis: found: C, 67.21; H, 11.08; N, 3.42; calcd for C_24_H_47_NO_5_: C, 67.09; H, 11.03; N, 3.26; IR (KBr) ν_max_: 1551 (C=N) cm^−1^; ^1^H NMR (500 MHz, CDCl_3_, δ): 0.71 (t, J_t_ = 7.2 Hz, 3H), 1.08 (s, 9H), 1.27 (s, 3H), 1.28 (s, 6H), 1.29 (s, 3H), 1.31 (s, 9H), 1.34 (dq, J_d_ = 14.7 Hz, J_q_ = 7.2 Hz, 1H), 2.23 (dq, J_d_ = 14.7 Hz, J_q_ = 7.2 Hz, 1H), 2.55 (ddd, J_d1_ = 9.3 Hz, J_d2_ = 7.6 Hz, J_d3_ = 3.8 Hz, 1H), 2.70 (ddd, J_d1_ = 7.6 Hz, J_d2_ = 2.7 Hz, J_d3_ = 2.7 Hz, 1H), 3.13 (s, 3H), 3.21 (s, 3H), 3.48 (dd, J_d1_ = 9.3 Hz, J_d2_ = 9.1 Hz, 1H), 3.57 (dd, J_d1_ = 9.1 Hz, J_d2_ = 3.8 Hz, 1H), 3.76 (d, J_d_ = 2.7 Hz, 2H); ^13^C NMR (125 MHz; CDCl_3_, δ): 9.8, 21.6, 24.02, 24.04, 24.29, 24.33, 25.5, 26.1, 34.8, 38.3, 39.6, 48.4, 48.8, 49.5, 62.0, 62.7, 82.7, 99.7, 100.0, 150.8.

#### 3.2.7. Preparation of Ethyllithium Solution

The EtLi solution was prepared in analogy to the literature procedure [52]. Small portions of bromoethane (up to ca. 150 μL) were added to a stirred suspension of finely chopped lithium tape (2 g, 286 mmol) in dry pentane (50 mL) in argon atmosphere until the reaction initiated (which was manifested by self-refluxing and violet coloring). Then, a solution of bromoethane (8 mL, 107 mmol) in dry pentane (15 mL) was added dropwise. The resulting purple suspension was heated to reflux for 1 h, then cooled down to room temperature. The suspension was allowed to settle, and the pentane solution of ethyllithium was siphoned to another vessel through a cannula under argon pressure. Then, dry benzene (50 mL) was added to precipitate, the mixture was stirred for 15 min and the benzene solution was separated as described above. The combined pentane–benzene solution contained 0.7–0.9 M EtLi (measured by titration with N-benzylbenzamide [53]). The solution was used immediately after preparation.

#### 3.2.8. Procedure for the Synthesis of (2R(S),3R(S),4R(S),5S(R))-2,2,5-Triethyl-5-tert-butyl-3,4-bis(hydroxymethyl)-pyrrolidine-1-oxyl (**1a**)

The ethyllithium solution (11 mL, 7.7–9.9 mmol) was added dropwise with stirring to a solution of nitrone **13** (1.34 g, 3.34 mmol) in dry hexane (10 mL) in argon atmosphere. When the reaction was complete (ca. 30 min; control by TLC of an aliquot quenched with water, silica gel, eluent ethyl acetate–hexane 2:1, *R_f_* = 0.4 for **13**), the mixture was carefully quenched with brine, the organic layer was separated and the aqueous layer was extracted with ethyl acetate (2 × 10 mL). The combined organic extract was evaporated in a vacuum, and the residue was dissolved in methanol (30 mL). A 1 mL amount of solution of PPTS (50 mg, 0.2 mmol) in water was added, and the mixture was bubbled with air for 2 days. Then, the solvents were distilled off in vacuum, and the residue was dissolved in ethyl acetate (20 mL), washed with water (3 × 10 mL) and dried over magnesium sulfate. The solvent was distilled off in vacuum, and the residue was triturated with diethyl ether to afford **1a**, yield 0.83 g (87%). Yellow crystals, m.p. 106–109 °C (from diethyl ether). Elemental analysis: found: C, 67.14; H, 11.22; N, 4.86; calcd for C_16_H_32_NO_3_: C, 67.09; H, 11.26; N, 4.89.; IR (KBr) ν_max_: 3371, 3294 (OH) cm^−1^; ^1^H NMR spectrum of **1a** reduced with Zn dust in CF_3_COOH in CD_3_OD corresponded to the literature data [34]; ^13^C NMR (100 MHz; CD_3_OD, Zn/CF_3_COOH system, δ): 8.5, 8.6, 11.5, 25.4, 26.8, 30.3, 39.3, 50.4, 56.9, 61.6, 62.5, 72.7, 77.7. 

#### 3.2.9. Procedure for the Synthesis of (1S(R),4R(S))-2,4-Di-tert-butyl-4-ethyl-1-propyl-3-azabicyclo[3.1.0]hex-2-ene (**15**)

A 14 mL amount of ethyllithium solution (9.8–12.6 mM) was added dropwise to a stirred solution of **14** (0.62 g, 1.50 mmol) in dry hexane (10 mL) in argon atmosphere. After **14** reacted completely (ca. 12 h, TLC control of aliquot quenched with water, silica gel, eluent ethyl acetate–hexane 2:1, *R_f_* = 0.5 for **14**), the mixture was quenched with brine, the organic layer was separated and the aqueous layer was extracted with ethyl acetate (2 × 10 mL). The combined organic solution was evaporated in vacuum, and the residue was dissolved in methanol (20 mL). A solution of PPTS (50 mg, 0.2 mmol) in 1 mL of water was added, and the mixture was bubbled with air for 2 days. The mixture was evaporated in vacuum; the residue was dissolved in ethyl acetate and washed with water (3 × 10 mL) then dried with magnesium sulfate and evaporated again. The EPR spectrum of the resulting mixture showed no trace of any nitroxide. The mixture was separated by chromatography on silica gel, eluent—ethyl acetate–hexane 1:20–1:40 to give **15**, yield: 0.09 g (23%), colorless oil. HRMS (EI/DFS) m/z [M]^+^ calcd for C_18_H_33_N 263.2608, found 263.2612; IR (neat) ν_max_: 1608 (C=N) cm^−1^; ^1^H NMR (400 MHz, CDCl_3_, δ): 0.12 (dd, J_d1_ = 4.2 Hz, J_d2_ = 4.5 Hz, 1H), 0.77 (dd, J_d1_ = 8.2 Hz, J_d2_ = 4.2 Hz, 1H), 0.88 (s, 9H), 0.89 (t, J_t_ = 7.4 Hz, 3H), 0.92 (t, J_t_ = 7.4 Hz, 3H), 0.90–1.00 (m, 1H), 1.04–1.14 (m, 1H), 1.20 (s, 9H), 1.28 (dq, J_d_ = 14.5 Hz, J_q_ = 7.4 Hz, 1H), 1.45 (dd, J_d1_ = 8.2 Hz, J_d2_ = 4.5 Hz, 1H), 1.83 (ddd, J_d1_ = 13.6 Hz, J_d2_ = 12.3 Hz, J_d3_ = 4.3 Hz, 1H), 1.95 (ddd, J_d1_ = 13.6 Hz, J_d2_ = 11.4 Hz, J_d3_ = 5.2 Hz, 1H), 1.96 (dq, J_d_ = 14.5 Hz, J_q_ = 7.4 Hz, 1H); ^13^C NMR (100 MHz, CDCl_3_, δ): 11.1, 14.4, 15.4, 19.4, 25.8, 25.9, 26.2, 28.7, 31.5, 36.8, 37.6, 38.7, 78.3, 182.2.

#### 3.2.10. Procedure for the Synthesis of 2,5-Di-tert-butyl-2-ethyl-3-(methoxycarbonyl)-3,4-dihydro-2H-pyrrole 1-oxides (**17**) and (**18**)

A solution of sodium hydroxide (1.17 g, 29.3 mmol) in water (10 mL) was added dropwise to a solution of **16** (2.5 g, 7.33 mmol) cooled to 0 °C in methanol (25 mL). The reaction was allowed to stand at room temperature for 2 days (TLC control, silica gel, hexane–ethyl acetate 4:1, UV detection, *R_f_* = 0.5 for **16**). Methanol was distilled off under reduced pressure, and the pH of the residual aqueous solution was adjusted to pH 4–5 with aqueous sodium hydrosulfate. The solution was extracted with ethyl acetate (5 × 15 mL); the extract was dried with magnesium sulfate and heated to reflux for 2 days. The solution was concentrated in vacuum, and the residue was separated via column chromatography using ethyl acetate as eluent to give the mixture of isomers **17** and **18,** ca. 2:1 (1.45 g, 5.12 mmol, 70%). Pure **17** and **18** were isolated via column chromatography on silica gel, eluent methanol–dichloromethane 1:200 mixture, to give **17** (0.98 g, 3.46 mmol, 47%) and **18** (0.47 g, 1.66 mmol, 23%).

*(2S(R),3R(S))-2,5-Di-tert-butyl-2-ethyl-3-(methoxycarbonyl)-3,4-dihydro-2H-pyrrole 1-oxide (***17***).* Colorless crystals; m.p. 63–71 °C (from hexane).; HRMS (EI/DFS) m/z [M]^+^ calcd for C_16_H_29_NO_3_ 283.2142, found 283.2145; IR (KBr) ν_max_: 1740 (C=O), 1572 (C=N) cm^−1^; ^1^H NMR (400 MHz; CDCl_3_, δ): 0.86 (t, J_t_ = 7.2 Hz, 3H), 0.98 (s, 9H), 1.24 (s, 9H), 1.69 (dq, J_d_ = 14.2 Hz, J_q_ = 7.2 Hz, 1H), 2.31 (dq, J_d_ = 14.2 Hz, J_q_ = 7.2 Hz, 1H), 2.51 (dd, J_d1_ = 18.2 Hz, J_d2_ = 8.7 Hz, 1H), 3.02 (dd, J_d1_ = 18.2 Hz, J_d2_ = 10.8 Hz, 1H), 3.28 (dd, J_d1_ = 10.8 Hz, J_d2_ = 8.7 Hz, 1H), 3.64 (s, 3H); ^13^C NMR (100 MHz; CDCl_3_, δ): 8.1, 22.8, 25.2, 27.0, 31.1, 33.5, 39.2, 41.3, 51.7, 86.8, 149.8, 172.2.

*(2S(R),3S(R))-2,5-Di-tert-butyl-2-ethyl-3-(methoxycarbonyl)-3,4-dihydro-2H-pyrrole 1-oxide (***18***).* Colorless crystals; m.p. 56–61 °C (from hexane); HRMS (EI/DFS) m/z [M]^+^ calcd for C_16_H_29_NO_3_ 283.2142, found 283.2147; IR (KBr) ν_max_: 1738 (C=O), 1579 (C=N) cm^−1^; ^1^H NMR (500 MHz; CDCl_3_, δ): 0.65 (t, J_t_ = 7.2 Hz, 3H), 1.09 (s, 9H), 1.28 (s, 9H), 1.79 (dq, J_d_ = 14.2 Hz, J_q_ = 7.2 Hz, 1H), 2.30 (dq, J_d_ = 14.2 Hz, J_q_ = 7.2 Hz, 1H), 2.73 (dd, J_d1_ = 19.1 Hz, J_d2_ = 10.7 Hz, 1H), 3.09 (dd, J_d1_ = 19.1 Hz, J_d2_ = 6.9 Hz, 1H), 3.20 (dd, J_d1_ = 10.7 Hz, J_d2_ = 6.9 Hz, 1H), 3.72 (s, 3H); ^13^C NMR (125 MHz; CDCl_3_, δ): 7.9, 21.6, 25.1, 26.0, 30.9, 33.5, 38.5, 39.8, 52.2, 86.8, 149.3, 172.3.

#### 3.2.11. Procedure for the Synthesis of 2,5-Di-tert-butyl-2-ethyl-3-(hydroxymethyl)-3,4-dihydro-2H-pyrrole 1-oxides (**20**, **21**)

A solution of **17** or **18** (0.98 g, 3.46 mmol) in dry THF (10 mL) was added dropwise to a stirred suspension of LiAlH_4_ (0.26 g, 6.92 mmol) in dry THF (10 mL). After addition was complete, the reaction mixture was stirred at reflux for 1 h, then cooled in an ice bath and carefully quenched with 1 mL of 5% aqueous sodium hydroxide and then 1 mL of water. The organic solution was separated, the inorganic mass was washed with diethyl ether (5 mL × 3 times). The combined extract was dried with magnesium sulfate and stirred vigorously with MnO_2_ for 2 days for oxidation of the hydroxylamines formed to the corresponding nitrones (TLC control, silica gel, eluent methanol–dichloromethane 1:50, UV detection, *R_f_* = 0.8 for the hydroxylamines, 0,2 for **20** or **21**). Then, the precipitate of oxidizing agent was filtered off, and the solvent was removed in vacuum. The crude product was purified via column chromatography on silica gel using methanol–dichloromethane 1:50 mixture as eluent and recrystallized from diethyl ether.

*(2S(R),3R(S))-2,5-Di-tert-butyl-2-ethyl-3-(hydroxymethyl)-3,4-dihydro-2H-pyrrole 1-oxide (***20***).* Yield: 0.59 g, (2.31 mmol, 67%). Colorless crystals; m.p. 166–167 °C (from diethyl ether); HRMS (EI/DFS) m/z [M]^+^ calcd for C_15_H_29_NO_2_ 255.2193, found 255.2188; Elemental analysis: found: C, 70.81; H, 11.46; N, 5.44; calcd for C_15_H_29_NO_2_: C, 70.54; H, 11.45; N, 5.48; IR (KBr) ν_max_: 3259 (OH) 1578 (C=N) cm^−1^; ^1^H NMR (600 MHz; CDCl_3_, δ): 0.86 (t, J_t_ = 7.2 Hz, 3H), 1.00 (s, 9H), 1.24 (s, 9H), 1.47 (dq, J_d_ = 14.2 Hz, J_q_ = 7.2 Hz, 1H), 2.16 (dq, J_d_ = 14.2 Hz, J_q_ = 7.2 Hz, 1H), 2.40 (dd, J_d1_ = 18.2 Hz, J_d2_ = 10.8 Hz, 1H), 2.71 (dddd, J_d1_ = 10.8 Hz, J_d2_ = 9.7 Hz, J_d3_ = 8.5 Hz, J_d4_ = 4.7 Hz, 1H), 2.72 (dd, J_d1_ = 18.2 Hz, J_d2_ = 8.5 Hz, 1H), 3.83 (dd, J_d1_ = 10.1 Hz, J_d2_ = 9.7 Hz, 1H), 3.92 (dd, J_d1_ = 10.1 Hz, J_d2_ = 4.7 Hz, 1H); ^13^C NMR (150 MHz; CDCl_3_, δ): 8.2, 23.2, 25.4, 27.6, 33.2, 33.6, 39.4, 41.5, 62.1, 85.4, 152.8.

*(2S(R),3S(R))-2,5-di-tert-butyl-2-ethyl-3-(hydroxymethyl)-3,4-dihydro-2H-pyrrole 1-oxide (***21***).* Yield: 0.24 g, (0.94 mmol, 57%). Colorless crystals; m.p. 123–128 °C (from diethyl ether); HRMS (EI/DFS) m/z [M]^+^ calcd for C_15_H_29_NO_2_ 255.2193, found 255.2190; Elemental analysis: found: C, 69.85; H, 11.31; N, 5.33; calcd for C_15_H_29_NO_2_: C, 70.54; H, 11.45; N, 5.48; IR (KBr) ν_max_: 3319 (OH), 1585 (C=N) cm^−1^; ^1^H NMR (400 MHz; CDCl_3_, δ): 0.76 (t, J_t_ = 7.2 Hz, 3H), 1.04 (s, 9H), 1.23 (s, 9H), 1.23 (dq, J_d_ = 14.2 Hz, J_q_ = 7.4 Hz, 1H), 2.19 (dq, J_d_ = 14.2 Hz, J_q_ = 7.2 Hz, 1H), 2.37 (dd, J_d1_ = 18.7 Hz, J_d2_ = 8.2 Hz, 1H), 2.54 (dddd, J_d1_ = 10.7 Hz, J_d2_ = 9.7 Hz, J_d3_ = 8.2 Hz, J_d4_ = 3.8 Hz, 1H), 2.87 (dd, J_d1_ = 18.7 Hz, J_d2_ = 9.7 Hz, 1H), 3.67 (dd, J_d1_ = 10.7 Hz, J_d2_ = 9.9 Hz, 1H), 3.79 (dd, J_d1_ = 9.9 Hz, J_d2_ = 3.8 Hz, 1H); ^13^C NMR (125 MHz; CDCl_3_, δ):9.7, 21.0, 25.4, 25.7, 33.5, 34.6, 38.0, 38.1, 63.6, 84.6, 152.6.

#### 3.2.12. Procedure for the Synthesis of (2S(R),3R(S))-2,5-Di-tert-butyl-2,5-diethyl-3-(hydroxymethyl)pyrrolidin-1-oxyl (**23**)

The hydroxylmethyl group in **20** was protected with 2,2-dimethoxypropane according to the procedure described in [54] under TLC control (silica gel, CHCl_3_, UV detection, *R_f_* = 0.2 for **20**, 0.7 for **22**). The crude product was purified via column chromatography on silica gel using ethyl acetate–hexane 1:4 mixture to give **22**, yield 85%. The ethyllithium solution (6 mL, 4.8 mmol) was added dropwise to a stirred solution of **22** (0.59 g, 1.8 mmol) in dry benzene (10 mL) in argon atmosphere. After the reaction was complete (ca. 30 min, TLC of aliquot quenched with water, silica gel, eluent ethyl acetate–hexane 1:5, *R_f_* = 0.2 for **22**), the reaction mixture was carefully quenched with brine, the organic layer was separated and the aqueous layer was extracted with diethyl ether (2 × 10 mL). The combined organic extract was evaporated in vacuum, and the residue was dissolved in ethanol (20 mL). A solution of PPTS (50 mg, 0.2 mmol) in 1 mL of water was added, and the mixture was bubbled with air for 1 day for deprotection and oxidation (TLC control, silica gel, CHCl_3_, *R_f_* = 0.9 for protected nitroxide, *R_f_* = 0.3 for **23**). Then, the solvent was removed in vacuum, and the residue was dissolved in ethyl acetate (20 mL), washed with water (3 × 10 mL) and dried over magnesium sulfate. After evaporation in vacuum, the crude product was purified via column chromatography on silica gel using CHCl_3_ as eluent to give **23,** yield 0.46 g (90% from **22**, 77% from **20**), orange oil. HRMS (EI/DFS) m/z [M]^+^ calcd for C_17_H_34_NO_2_ 284.2584, found 284.2581; Elemental analysis: found: C, 71.50; H, 11.96; N, 4.83; calcd for C_17_H_34_NO_2_: C, 71.78; H, 12.05; N, 4.92; IR (neat) ν_max_: 3442 (OH) cm^−1^; ^1^H NMR (400 MHz; CD_3_OD, Zn/CF_3_COOH system, δ): 1.12 (t, J_t_ = 7.4 Hz, 3H), 1.17 (s, 9H), 1.18 (s 9H), 1.22 (t, J_t_ = 7.4 Hz, 3H), 1.90 (dq, J_d_ = 15.1 Hz, J_q_ = 7.4 Hz, 1H), 1.92 (dq, J_d_ = 14.7 Hz, J_q_ = 7.4 Hz, 1H), 2.05 (dd, J_d1_ = 13.6 Hz, J_d2_ = 13.4 Hz, 1H), 2.20 (dq, J_d_ = 15.1 Hz, J_q_ = 7.4 Hz, 1H), 2.40 (dq, J_d_ = 14.7 Hz, J_q_ = 7.4 Hz, 1H), 2.42 (dd, J_d1_ = 13.4 Hz, J_d2_ = 6.1 Hz, 1H), 2.82 (dddd, J_d1_ = 13.6 Hz, J_d2_ = 9.7 Hz, J_d3_ = 6.1 Hz, J_d4_ = 4.6 Hz, 1H), 3.89 (dd, J_d1_ = 10.7 Hz, J_d2_ = 9.7 Hz, 1H), 4.08 (dd, J_d1_ = 10.7 Hz, J_d2_ = 4.6 Hz, 1H); ^13^C NMR (75 MHz; CD_3_OD, Zn/CF_3_COOH system, δ): 11.0, 11.2, 26.2, 27.0, 28.2, 32.5, 34.6, 39.2, 41.4, 50.5, 62.2, 76.1, 79.4.

#### 3.2.13. Procedure for the Synthesis of ((2S(R),3R(S),5S(R))-2,5-Di-tert-butyl-2,5-diethyl-3-((((3-(trimethylammonio)propyl)carbamoyl)oxy)methyl)pyrrolidin-1-oxyl) Monoiodide (**26**)

The nitroxide **23** (0.16 g, 0.56 mmol) was added to a cold (+5 °C) solution of 1,1′-carbonyldiimidazole (0.14 g, 0.86 mmol) in dry THF (1 mL), and the mixture was stirred for 3 h (TLC control, silica gel, ethyl acetate–hexane 1:1, *R_f_* = 0.7 for **23**, 0.5 for **24**). THF was removed under reduced pressure without heating, and the residue was purified via column chromatography on silica gel using ethyl acetate–hexane 1:2 mixture as eluent to give carbonylimidazole derivative **24** (0.18 g, 0.48 mmol, 84%). The latter was dissolved in dry THF (1 mL), and N,N-dimethylpropane-1,3-diamine (0.05 g, 0.48 mmol) was added. The mixture was stirred for 30 min (TLC control, silica gel, CHCl_3_, *R_f_* = 0.8 for **24**, *R_f_* = 0.5 for **25**). THF was removed in vacuum without heating, and the crude product was purified via column chromatography on silica gel, eluent—EtOH–CHCl_3_ 1:10 mixture, to give (*2S,3R,5S)-2,5-di-tert-butyl-3-((((3-(dimethylamino)propyl)carbamoyl)oxy)methyl)-2,5-diethylpyrrolidin-1-oxyl (***25***)*, yield 0.17 g (0.41 mmol, 87% from **24**, 73% from **23**), orange oil. IR (neat) ν_max_: 3223, 3117 (NH); 1718 (C=O) cm^−1^.

Iodomethane (0.3 g, 2.06 mmol) was added to a solution of **25** (0.17 g, 0.41 mmol) in dry diethyl ether (1 mL). The reaction mixture was kept at +5 °C for 12 h, and the product precipitated out of the solution as a viscous oily mass. The latter was triturated with dry diethyl ether (5 × 1 mL); the powder of **26** was filtered and dried in vacuum, yield 0.23 g (0.415 mmol, ~100%), highly hygroscopic glassy yellow powder. IR (neat) ν_max_: 3454, 3284, 3101 (NH), 1707 (C=O) cm^−1^. ^1^H NMR (400 MHz; CD_3_OD, Zn/CF_3_COOH system): 1.09 (t, J_t_ = 7.5 Hz, 3H), 1.17 (s, 9H), 1.20 (s, 9H), 1.23 (t, J_t_ = 7.3 Hz, 3H), 1.90 (dq, J_d_ = 15.4 Hz, J_q_ = 7.6 Hz, 1H), 1.93 (dq, J_d_ = 15.2 Hz, J_q_ = 7.4 Hz, 1H), 1.96–2.05 (m, 2H), 2.12 (dd, J_d1_ = 13.7 Hz, J_d2_ = 13.8 Hz, 1H), 2.20 (dq, J_d_ = 15.4 Hz, J_q_ = 7.4 Hz, 1H), 2.32 (dd, J_d1_ = 13.9 Hz, J_d2_ = 6.0 Hz, 1H), 2.41 (dq, J_d_ = 15.2 Hz, J_q_ = 7.3 Hz, 1H), 2.95 (dddd, J_d1_ = 13.7 Hz, J_d2_ = 10.0 Hz, J_d3_ = 6.0 Hz, J_d4_ = 4.8 Hz, 1H), 3.15 (s, 9H), 3.20–3.26 (m, 2H), 3.37–3.43 (m, 2H), 4.35 (dd, J_d1_ = 10.0 Hz, J_d2_ = 10.6 Hz, 1H), 4.59 (dd, J_d1_ = 10.6 Hz, J_d2_ = 4.8 Hz, 1H); ^13^C NMR (100 MHz; CD_3_OD, Zn/CF_3_COOH system, δ): 11.1, 11.4, 24.7, 26.1, 27.0, 28.2, 32.5, 34.6, 38.7, 39.3, 41.3, 46.8, 53.7, 53.8, 53.9, 65.0, 65.7, 76.2, 79.4, 158.4.

### 3.3. EPR Measurements and Kinetics

The EPR spectra were recorded for the 0.2 mM solutions of the nitroxides in 5 mM phosphate–citrate–borate buffer on a Bruker ER-200D-SRC spectrometer in a 50 µL glass capillary for 0.2 mM radical solutions (Figure S70). Spectrometer settings: frequency, 9.87 GHz; microwave power, 5.0 mW; modulation amplitude, 0.05 mT; time constant, 50–100 ms; and conversion time, 5.12 ms. For kinetic measurements in water, stock solutions of nitroxide, ascorbic acid and glutathione in phosphate–citrate–borate buffer (5 mM, pH 7.4) were prepared, and pH was adjusted to 7.4 with NaOH or HCl. All the solutions were deoxygenated with argon, were carefully and quickly mixed in a small tube to attain final concentrations (nitroxide, 0.2–0.4 mM; GSH, 5 mM; and ascorbate, 100–300 mM) and were placed into an EPR capillary (50 μL). The capillary was sealed on both sides and placed into the EPR resonator. The decay of amplitude of the low-field component of the EPR spectrum was followed to obtain the kinetics (Figures S68 and S71). The initial part of the decay curves (up to 20 min) was used for fitting. Kinetics of the decay were fitted to a monoexponential function to calculate the first-order rate constants. Then, these constants were plotted versus the concentration of ascorbic acid to calculate the second-order reaction constants (Figures S69 and S72).

## 4. Conclusions

This paper demonstrates the feasibility of the synthesis of highly strained nitroxides using a previously suggested strategy [16,33,34]. The three-component domino reaction of aminoacid, ketone and activated alkene allowed us to assemble the pyrrolidine with two *tert*-butyl groups and one ethyl group in positions 2 and 5 in one step. The use of *m*CPBA allowed us to oxidize this pyrrolidine into highly strained 1-pyrroline-1-oxide. Finally, the fourth substituent was successfully introduced via addition of EtLi.

This synthesis allowed us to demonstrate that introduction of bulkier substituents adjacent to the nitroxide group will not necessarily increase the resistance of the nitroxide to reduction. Obviously, reassessment of the role of molecule geometry, and of tensions and distortions produced by the bulky substituents for nitroxide stability to reduction, is necessary. This finding may help in alternative design of reduction-resistant nitroxides.

Another important finding is the new synthesis of a highly stable nitroxide **1a** (*k*_red_ = (7.0 ± 2) × 10^−5^ M^−1^s^−1^) via EtLi addition with an overall yield 37.5% in four steps from *tert*-leucine. To date, this is the simplest synthesis of a nitroxide that is highly resistant to reduction.

## Data Availability

All the data are in the text and the Supplementary Information in this article.

## Supplementary Materials

The following supporting information can be downloaded at: https://www.mdpi.com/article/10.3390/molecules29030599/s1, NMR spectra (Figures S1–S49), IR spectra (Figures S50–S64) of all new compounds, X-ray diffraction data for **20** and **21** (Figures S65 and S66), EPR spectrum of **26** (Figure S69), reduction profiles and kinetics of reduction of **1a** and **26** (Figures S67, S68, S70 and S71).

## Appendix A

### Appendix A1. Correlation Experiments ^1^H–^13^C HMBC and ^1^H–^1^H NOESY for ***8*** and ***9***

In the ^1^H–^13^C HMBC correlation spectrum (Figure S35) of **8** in acidified solution, the cross-peaks were observed between the nodal carbon atom at 78.5 ppm and methine hydrogens in positions 3 and 4 at 3.89 and 3.93 ppm and methylene protons of the ethyl group at 2.11 and 2.33 ppm and of *tert*-butyl group at 1.27 ppm. The signal of the 5-CH carbon atom at 70.8 ppm showed cross-peaks with methine hydrogens in positions 3 and 4 and with the signal of protons of the *tert*-butyl group at 1.18 ppm. In the ^1^H–^1^H NOESY correlation spectrum (Figure S36), there were cross-peaks between methine hydrogen in position 5 of the heterocycle at 4.13 ppm and the signals of CH_3_ protons of the ethyl group at 1.15 ppm, protons of *tert* butyl group at 1.18 ppm and 4-CH. Thus, 5-CH, 4-CH and the ethyl group are in cis-positions to each other. In the spectrum of **9** (Figure S37), the cross-peaks between methyl hydrogens of the ethyl group at 1.03 ppm and the methyne protons at positions 5 and 3 at 3.40 and 3.02 ppm of the ring unambiguously indicate their cys-position relative to each other. This is possible only if the *tert*-butyl groups are cis to each other.

### Appendix A2. Correlation Experiments ^1^H–^13^C HMBC and ^1^H–^1^H NOESY for ***12***

In ^1^H–^13^C HMBC correlation (Figure S38), the strong cross-peaks were observed between nitrone carbon at 152.0 ppm with the signals of 3-CH at 2.81 ppm and the *tert*-butyl group at 1.28 ppm, while the nodal carbon signal at 85.0 ppm showed cross-peaks with the signals of 4-CH at 2.32 ppm, the *tert*-butyl group at 1.05 ppm and the signals of protons of the ethyl group.

In the ^1^H–^1^H NOESY correlation (Figure S39) for **12**, the signal of CH_3_ hydrogens of the ethyl group at 0.69 ppm gave the cross-peak with the methine hydrogen at position 3 at 2.81 ppm, while the signal 5-*tert*-butyl hydrogens at 1.05 ppm gave the cross-peak with 4-CH signal at 2.32 ppm.

### Appendix A3. ^1^H–^1^H COSY, ^1^H–^13^C HSQC, ^1^H–^13^C HMBC and ^1^H–^1^H NOESY Correlation Experiments for ***15***

Assignment of the signals was carried out on the basis of ^1^H–^1^H COSY, ^1^H–^13^C HSQC, ^1^H–^13^C HMBC and ^1^H–^1^H NOESY correlations (Figures S40–S43). The ^1^H–^13^C HMBC correlation experiment (Figure S42) showed cross-peaks between the imine carbon at 182.2 ppm and the signal of protons of the *tert*-butyl group at 1.20 ppm, all protons of cyclopropane ring and α-hydrogens of the *n*-propyl group at 1.82 and 1.95 ppm. The signal of the nodal carbon atom at 78.3 had cross-peaks with the protons of the *tert*-butyl group at 0.88 ppm and with all protons of the cyclopropane ring and the methylene hydrogens of the ethyl group at 1.96 and 1.28 ppm. These data support 2,4-di-*tert*-butyl-4-ethyl-1-propyl-3-azabicyclo[3.1.0]hex-2-ene formation.

The ^1^H–^1^H NOESY correlation experiment (Figure S43) showed the cross-peaks between the protons of the *tert*-butyl group at 0.88 ppm (at the nodal carbon neighboring to the nitrogen atom), with the methine proton, with methylene hydrogens of the ethyl group and with α-hydrogens of *n*-propyl group at 1.82 and 1.95 ppm, supporting cis-configuration of *n*-Pr, 4-*t*-Bu and 5-H.

### Appendix A4. ^1^H–^1^H COSY, ^1^H–^13^C HSQC, ^1^H–^13^C HMBC and ^1^H–^1^H NOESY Correlation Experiments for ***26*** after Reduction with Zn/CF_3_COOH

Assignment of the signals was carried out on the basis of ^1^H–^1^H COSY, ^1^H–^13^C HSQC, ^1^H–^13^C HMBC and ^1^H–^1^H NOESY correlations (Figures S44–S47). The ^1^H–^13^C HMBC (Figure S46) correlation experiment showed the cross-peaks between the nodal carbon atom at 76.2 ppm (neighboring to the nitrogen atom) with the signal of *tert*-butyl group protons at 1.17 ppm and the ethyl group protons at 1.09, 1.90 and 2.20 ppm. A signal of another nodal carbon at 79.3 ppm showed cross-peaks with the signals of *tert*-butyl protons at 1.20 ppm, of ethyl hydrogens at 1.23, 1.93 and 2.41 ppm and of methylene hydrogens of the oxymethyl group at 4.35 and 4.59 ppm.

In the ^1^H–^1^H NOESY spectrum (Figure S47), there were cross-peaks between the signals of CH_3_ hydrogens of both ethyl groups with a 3-CH signal at 2.95 ppm. Cross-peaks between the signal of methine proton at position 4 at 2.12 ppm and the signals of both *tert*-butyl groups were also observed. In addition, the spin–spin interaction constant between the methine hydrogens at 2.12 and 2.95 ppm obtained from line shape simulation, J = 13.7 Hz (Figure S49, Table S2), clearly indicates their trans-position relative to each other.
