# Supplementary material for: 2,5-Di-tert-butyl-2,5-diethylpyrrolidine-1-oxyls: Where Is a Reasonable Limit of Sterical Loading for Higher Resistance to Reduction?
Source: Molecules. 2024 Jan 25;29(3):599. doi: 10.3390/molecules29030599 (PMC10856307; doi:10.3390/molecules29030599)

Supporting Information

Table of content

NMR Spectra 5
Figure S1. ^{1}H NMR (400 MHz; $CDCl_3$) of 2,2-dimethylpentan-3-ol 5
Figure S2. ^{1}H NMR (400 MHz; $CDCl_3$) of 2,2-dimethylpentan-3-one 5
Figure S3. ^{1}H NMR (500 MHz; $CDCl_3$) of (2R(S),3R(S),4R(S),5S(R))-2,5-di-*tert*-butyl-3,4-bis(methoxycarbonyl)-2-ethylpyrrolidine (8) 6
Figure S4. ^{13}C NMR (125 MHz; $CDCl_3$) of (2R(S),3R(S),4R(S),5S(R))-2,5-di-*tert*-butyl-3,4-bis(methoxycarbonyl)-2-ethylpyrrolidine (8) 6
Figure S5. ^{1}H NMR (400 MHz; $(CD_3)_2CO$; CF_3COOH) of (2R(S),3R(S),4R(S),5S(R))-2,5-di-*tert*-butyl-3,4-bis(methoxycarbonyl)-2-ethylpyrrolidine (8) 7
Figure S6. ^{13}C NMR (100 MHz; $(CD_3)_2CO$; CF_3COOH) of (2R(S),3R(S),4R(S),5S(R))-2,5-di-tert-butyl-3,4-bis(methoxycarbonyl)-2-ethylpyrrolidine (8) 7
Figure S7. ^{1}H NMR (500 MHz; $CDCl_3$) of (2S(R),3R(S),4R(S),5R(S))-2,5-di-*tert*-butyl-3,4-bis(methoxycarbonyl)-2-ethylpyrrolidine (9) 8
Figure S8. ^{13}C NMR (125 MHz; $CDCl_3$) of (2S(R),3R(S),4R(S),5R(S))-2,5-di-*tert*-butyl-3,4-bis(methoxycarbonyl)-2-ethylpyrrolidine (9) 8
Figure S9. ^{1}H NMR (500 MHz; $CDCl_3$) of dimethyl 2-(1-((2,2-dimethylpentan-3-ylidene)amino)-2,2-dimethylpropyl)succinate (10) 9
Figure S10. ^{13}C NMR (125 MHz; $CDCl_3$) of dimethyl 2-(1-((2,2-dimethylpentan-3-ylidene)amino)-2,2-dimethylpropyl)succinate (10) 9
Figure S11. ^{1}H NMR (500 MHz; $CDCl_3$) of (2R(S),3R(S),4R(S),5S(R))-2,5-di-*tert*-butyl-3,4-bis(hydroxymethyl)-2-ethylpyrrolidine (11) 10
Figure S12. ^{13}C NMR (125 MHz; $CDCl_3$) of (2R(S),3R(S),4R(S),5S(R))-2,5-di-tert-butyl-3,4-bis(hydroxymethyl)-2-ethylpyrrolidine (11) 10
Figure S13. ^{1}H NMR (400 MHz, $CDCl_3$) of (2R(S),3R(S),4R(S))-2,5-di-*tert*-butyl-2-ethyl-3,4-bis(hydroxymethyl)-3,4-dihydro-2*H*-pyrrole 1-oxide (12) 11
Figure S14. ^{13}C NMR (100 MHz, $CDCl_3$) of (2R(S),3R(S),4R(S))-2,5-di-*tert*-butyl-2-ethyl-3,4-bis(hydroxymethyl)-3,4-dihydro-2*H*-pyrrole 1-oxide (12) 11
Figure S15. ^{1}H NMR (500 MHz, $CDCl_3$) of (2R(S),3R(S),4R(S))-2,5-di-*tert*-butyl-2-ethyl-3,4-bis(((2-methoxypropan-2-yl)oxy)methyl)-3,4-dihydro-2*H*-pyrrole 1-oxide (14) 12
Figure S16. ^{13}C NMR (125 MHz, $CDCl_3$) of (2R(S),3R(S),4R(S))-2,5-di-tert-butyl-2-ethyl-3,4-bis(((2-methoxypropan-2-yl)oxy)methyl)-3,4-dihydro-2*H*-pyrrole 1-oxide (14) 12
Figure S17. ^{1}H NMR (400 MHz; CD_3OD, Zn/CF_3COOH system) of (2R(S),3R(S),4R(S),5S(R))-2,2,5-triethyl-5-*tert*-butyl-3,4-bis(hydroxymethyl)-pyrrolidine-1-oxyl (1a) 13

Figure S18. ^{13}C NMR (100 MHz; CD_3OD, Zn/CF_3COOH system) of (2R(S),3R(S),4R(S),5S(R))-2,2,5-triethyl-5-*tert*-butyl-3,4-bis(hydroxymethyl)-pyrrolidine-1-oxyl (1a) 13
Figure S19. ^{1}H NMR (400 MHz; $CDCl_3$) of (1S(R),4R(S),5R(S))-2,4-di-*tert*-butyl-4-ethyl-1-propyl-3-azabicyclo[3.1.0]hex-2-ene (15) 14
Figure S20. ^{13}C NMR (100 MHz; $CDCl_3$) of (1S(R),4R(S),5R(S))-2,4-di-*tert*-butyl-4-ethyl-1-propyl-3-azabicyclo[3.1.0]hex-2-ene (15) 14
Figure S21. ^{1}H NMR (500 MHz; $CDCl_3$) of (2S(R),3R(S),4R(S))-2,5-di-*tert*-butyl-2-ethyl-3,4-bis(methoxycarbonyl)-3,4-dihydro-2H-pyrrole 1-oxide (16) 15
Figure S22. ^{13}C NMR (125 MHz; $CDCl_3$) of (2S(R),3R(S),4R(S))-2,5-di-*tert*-butyl-2-ethyl-3,4-bis(methoxycarbonyl)-3,4-dihydro-2H-pyrrole 1-oxide (16) 15
Figure S23. ^{1}H NMR (400 MHz; $CDCl_3$) of (2S(R),3R(S))-2,5-di-*tert*-butyl-2-ethyl-3-(methoxycarbonyl)-3,4-dihydro-2H-pyrrole 1-oxide (17) 16
Figure S24. ^{13}C NMR (100 MHz; $CDCl_3$) of (2S(R),3R(S))-2,5-di-*tert*-butyl-2-ethyl-3-(methoxycarbonyl)-3,4-dihydro-2H-pyrrole 1-oxide (17) 16
Figure S25. ^{1}H NMR (500 MHz; $CDCl_3$) of (2S(R),3S(R))-2,5-di-*tert*-butyl-2-ethyl-3-(methoxycarbonyl)-3,4-dihydro-2H-pyrrole 1-oxide (18) 17
Figure S26. ^{13}C NMR (125 MHz; $CDCl_3$) of (2S(R),3S(R))-2,5-di-*tert*-butyl-2-ethyl-3-(methoxycarbonyl)-3,4-dihydro-2H-pyrrole 1-oxide (18) 17
Figure S27. ^{1}H NMR (600 MHz; $CDCl_3$) of (2S(R),3R(S))-2,5-di-*tert*-butyl-2-ethyl-3-(hydroxymethyl)-3,4-dihydro-2H-pyrrole 1-oxide (20) 18
Figure S28. ^{13}C NMR (150 MHz; $CDCl_3$) of (2S(R),3R(S))-2,5-di-*tert*-butyl-2-ethyl-3-(hydroxymethyl)-3,4-dihydro-2H-pyrrole 1-oxide (20) 18
Figure S29. ^{1}H NMR (400 MHz; $CDCl_3$) of (2S(R),3S(R))-2,5-di-*tert*-butyl-2-ethyl-3-(hydroxymethyl)-3,4-dihydro-2H-pyrrole 1-oxide (21) 19
Figure S30. ^{13}C NMR (125 MHz; $CDCl_3$) of (2S(R),3S(R))-2,5-di-*tert*-butyl-2-ethyl-3-(hydroxymethyl)-3,4-dihydro-2H-pyrrole 1-oxide (21) 19
Figure S31. ^{1}H NMR (400 MHz; CD_3OD, Zn/CF_3COOH system) of (2S(R),3R(S))-2,5-di-tert-butyl-2,5-diethyl-3-(hydroxymethyl)pyrrolidin-1-oxyl (23) 20
Figure S32. ^{13}C NMR (75 MHz; CD_3OD, Zn/CF_3COOH system) of (2S(R),3R(S))-2,5-di-tert-butyl-2,5-diethyl-3-(hydroxymethyl)pyrrolidin-1-oxyl (23) 20
Figure S33. ^{1}H NMR (400 MHz; CD_3OD, Zn/CF_3COOH system) of ((2S(R),3R(S),5S(R))-2,5-di-*tert*-butyl-2,5-diethyl-3-((((3-(trimethylammonio)propyl)carbamoyl)oxy)methyl)pyrrolidin-1-oxyl) monoiodide (26) 21
Figure S34. ^{13}C NMR (100 MHz; CD_3OD, Zn/CF_3COOH system) of ((2S(R),3R(S),5S(R))-2,5-di-*tert*-butyl-2,5-diethyl-3-((((3-(trimethylammonio)propyl)carbamoyl)oxy)methyl)pyrrolidin-1-oxyl) monoiodide (26) 21
2D NMR Spectra 22
Figure S35. HMBC (400 MHz; $(CD_3)_2CO$; CF_3COOH) of (2R(S),3R(S),4R(S),5S(R))-2,5-di-*tert*-butyl-3,4-bis(methoxycarbonyl)-2-ethylpyrrolidine (8) 22
Figure S36. NOESY (400 MHz; $(CD_3)_2CO$; CF_3COOH) of (2R(S),3R(S),4R(S),5S(R))-2,5-di-*tert*-butyl-3,4-bis(methoxycarbonyl)-2-ethylpyrrolidine (8) 22
Figure S37. NOESY (600 MHz; $CDCl_3$) of (2S(R),3R(S),4R(S),5R(S))-2,5-di-*tert*-butyl-3,4-bis(methoxycarbonyl)-2-ethylpyrrolidine (9) 23
Figure S38. HMBC (400 MHz; $CDCl_3$) of (2R(S),3R(S),4R(S))-2,5-di-*tert*-butyl-2-ethyl-3,4-bis(hydroxymethyl)-3,4-dihydro-2*H*-pyrrole 1-oxide (12) 23
Figure S39. NOESY (400 MHz; $CDCl_3$) of (2R(S),3R(S),4R(S))-2,5-di-*tert*-butyl-2-ethyl-3,4-bis(hydroxymethyl)-3,4-dihydro-2*H*-pyrrole 1-oxide (12) 24
Figure S40. COSY (400 MHz, $CDCl_3$) of (1S(R),4R(S),5R(S))-2,4-di-*tert*-butyl-4-ethyl-1-propyl-3-azabicyclo[3.1.0]hex-2-ene (15) 24
Figure S41. HSQC (400 MHz, $CDCl_3$) of (1S(R),4R(S),5R(S))-2,4-di-*tert*-butyl-4-ethyl-1-propyl-3-azabicyclo[3.1.0]hex-2-ene (15) 25
Figure S42. HMBC (400 MHz, $CDCl_3$) of (1S(R),4R(S),5R(S))-2,4-di-*tert*-butyl-4-ethyl-1-propyl-3-azabicyclo[3.1.0]hex-2-ene (15) 25

Figure S43. NOESY (400 MHz, $CDCl_3$) of (1S(R),4R(S),5R(S))-2,4-di-*tert*-butyl-4-ethyl-1-propyl-3-azabicyclo[3.1.0]hex-2-ene (15) 26
Figure S44. COSY (400 MHz; CD_3OD, Zn/CF_3COOH system) of ((2S(R),3R(S),5S(R))-2,5-di-*tert*-butyl-2,5-diethyl-3-((((3-(trimethylammonio)propyl)carbamoyl)oxy)methyl)pyrrolidin-1-oxyl) monoiodide (26) 26
Figure S45. HSQC (400 MHz; CD_3OD, Zn/CF_3COOH system) of ((2S(R),3R(S),5S(R))-2,5-di-*tert*-butyl-2,5-diethyl-3-((((3-(trimethylammonio)propyl)carbamoyl)oxy)methyl)pyrrolidin-1-oxyl) monoiodide (26) 27
Figure S46. HMBC (400 MHz; CD_3OD, Zn/CF_3COOH system) of ((2S(R),3R(S),5S(R))-2,5-di-*tert*-butyl-2,5-diethyl-3-((((3-(trimethylammonio)propyl)carbamoyl)oxy)methyl)pyrrolidin-1-oxyl) monoiodide (26) 27
Figure S47. NOESY (400 MHz; CD_3OD, Zn/CF_3COOH system) of ((2S(R),3R(S),5S(R))-2,5-di-*tert*-butyl-2,5-diethyl-3-((((3-(trimethylammonio)propyl)carbamoyl)oxy)methyl)pyrrolidin-1-oxyl) monoiodide (26) 28
Line shape analysis 29
Figure S48.Simulated spectrum for (1S(R),4R(S),5R(S))-2,4-di-tert-butyl-4-ethyl-1-propyl-3-azabicyclo[3.1.0]hex-2-ene (15) 29
Table S1. Parameters of spins system (1S(R),4R(S),5R(S))-2,4-di-tert-butyl-4-ethyl-1-propyl-3-azabicyclo[3.1.0]hex-2-ene (15) 29
Figure S49. Simulated spectrum for mono((2S(R),3R(S),5S(R))-2,5-di-*tert*-butyl-2,5-diethyl-3-((((3-(trimethylammonio)propyl)carbamoyl)oxy)methyl)pyrrolidin-1-oxyl) monoiodide (26) 30
Table S2. Parameters of spins system for mono((2S(R),3R(S),5S(R))-2,5-di-*tert*-butyl-2,5-diethyl-3-((((3-(trimethylammonio)propyl)carbamoyl)oxy)methyl)pyrrolidin-1-oxyl) monoiodide (26) 30
IR Spectra 31
Figure S50. IR (neat) of (2R(S),3R(S),4R(S),5S(R))-2,5-di-*tert*-butyl-3,4-bis(methoxycarbonyl)-2-ethylpyrrolidine (8) 31
Figure S51. IR (neat) of (2S(R),3R(S),4R(S),5R(S))-2,5-di-*tert*-butyl-3,4-bis(methoxycarbonyl)-2-ethylpyrrolidine (9) 31
Figure S52. IR (neat) of dimethyl 2-(1-((2,2-dimethylpentan-3-ylidene)amino)-2,2-dimethylpropyl)succinate (10) 31
Figure S53. IR (KBr) of (2R(S),3R(S),4R(S),5S(R))-2,5-di-tert-butyl-3,4-bis(hydroxymethyl)-2-ethylpyrrolidine (11) 32
Figure S54. IR (KBr) of (2R(S),3R(S),4R(S))-2,5-di-tert-butyl-2-ethyl-3,4-bis(hydroxymethyl)-3,4-dihydro-2H-pyrrole 1-oxide (12) 32
Figure S55. IR (KBr) of 2,2,5-Triethyl-5-tert-butyl-3,4-bis(hydroxymethyl)-pyrrolidine-1-oxyl (1a) .. 32
Figure S56. IR(KBr) of (2R(S),3R(S),4R(S))-2,5-di-tert-butyl-2-ethyl-3,4-bis(((2-methoxypropan-2-yl)oxy)methyl)-3,4-dihydro-2H-pyrrole 1-oxide (14) 33
Figure S57. IR (neat) of (1S(R),4R(S),5R(S))-2,4-di-*tert*-butyl-4-ethyl-1-propyl-3-azabicyclo[3.1.0]hex-2-ene (15) 33
Figure S58. IR (neat) of (2S(R),3R(S),4R(S))-2,5-di-*tert*-butyl-2-ethyl-3,4-bis(methoxycarbonyl)-3,4-dihydro-2*H*-pyrrole 1-oxide (16) 33
Figure S59. IR (KBr) of (2S(R),3R(S))-2,5-di-tert-butyl-2-ethyl-3-(methoxycarbonyl)-3,4-dihydro-2H-pyrrole 1-oxide (17) 34
Figure S60. IR (KBr) of (2S(R),3S(R))-2,5-di-tert-butyl-2-ethyl-3-(methoxycarbonyl)-3,4-dihydro-2H-pyrrole 1-oxide (18) 34
Figure S61. IR (KBr) of (2S(R),3R(S))-2,5-di-tert-butyl-2-ethyl-3-(hydroxymethyl)-3,4-dihydro-2H-pyrrole 1-oxide (20) 34
Figure S62. IR (KBr) of (2S(R),3S(R))-2,5-di-tert-butyl-2-ethyl-3-(hydroxymethyl)-3,4-dihydro-2H-pyrrole 1-oxide (21) 35
Figure S63. IR (neat) of (2S(R),3R(S))-2,5-di-tert-butyl-2,5-diethyl-3-(hydroxymethyl)pyrrolidin-1-oxyl (23) 35
Figure S64. IR (neat) of (2S(R),3R(S),5S(R))-2,5-di-*tert*-butyl-3-((((3-(dimethylamino)propyl)carbamoyl)oxy)methyl)-2,5-diethylpyrrolidin-1-oxyl (25) 35

Figure S65. IR (neat) of mono((2S(R),3R(S),5S(R))-2,5-di-*tert*-butyl-2,5-diethyl-3-((((3-(trimethylammonio)propyl)carbamoyl)oxy)methyl)pyrrolidin-1-oxyl) monoiodide (26) 36
XRD Data....37
Figure S66. Crystal structure for (2S(R),3R(S))-2,5-di-tert-butyl-2-ethyl-3-(hydroxymethyl)-3,4-dihydro-2H-pyrrole 1-oxide (20) 37
Figure S67. Crystal structure for (2S(R),3S(R))-2,5-di-tert-butyl-2-ethyl-3-(hydroxymethyl)-3,4-dihydro-2H-pyrrole 1-oxide (21) 37
EPR Data 38
Figure S68. Reduction profiles of 1a (0.2 mM) in anaerobic conditions in phosphate-citrate-borate buffer (5 mM, pH 7.4) in presence of GSH (5 mM) and ascorbate 38
Figure S69. The plot of the nitroxide 1a first order decay constant k1 versus ascorbate concentration and the linear fit 38
Figure S70. EPR spectrum of 26 (0.2 mM) in phosphate-citrate-borate buffer (5 mM, pH 7.4). 39
Figure S71. Reduction profiles of 26 (0.2 mM) in anaerobic conditions in phosphate-citrate-borate buffer (5 mM, pH 7.4) in presence of GSH (5 mM) and ascorbate 39
Figure S72. The plot of the nitroxide 26 first order decay constant k_1 versus ascorbate concentration and the linear fit 40

NMR Spectra

Figure S1. ^{1}H NMR (400 MHz; $CDCl_3$) of 2,2-dimethylpentan-3-ol

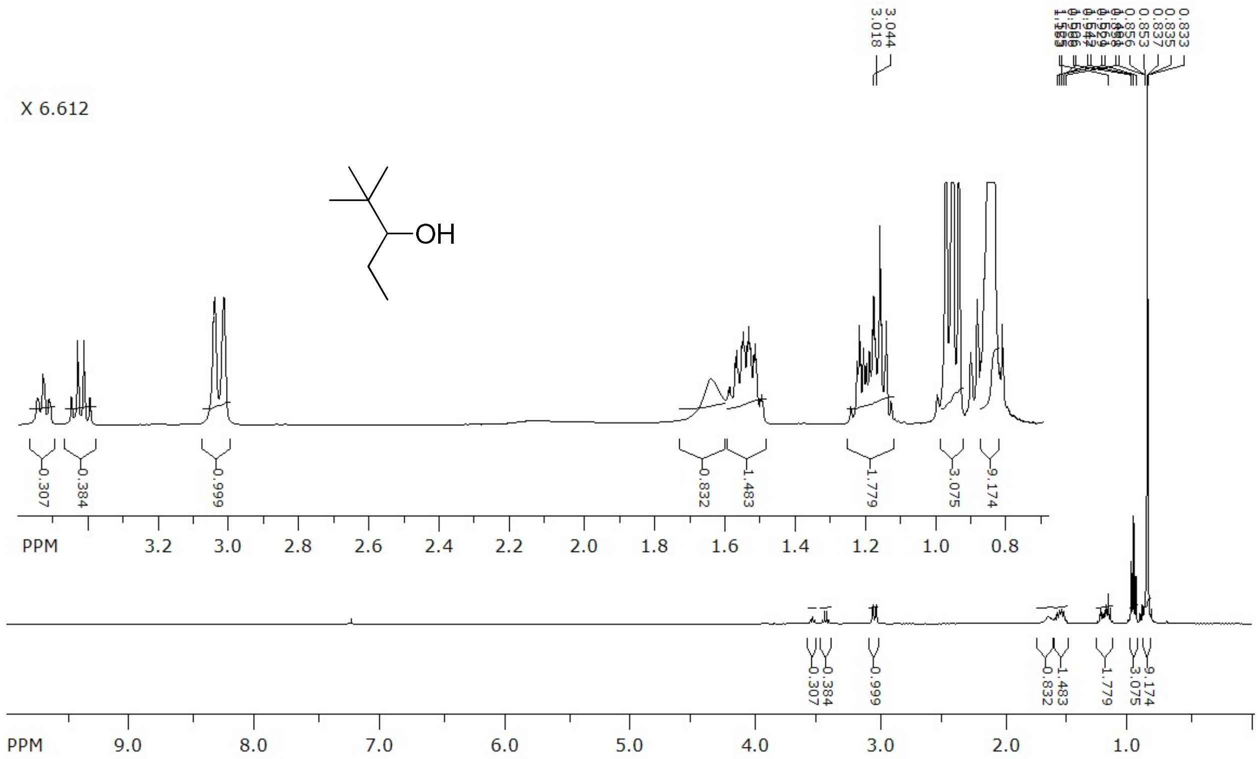

Figure S2. ^{1}H NMR (400 MHz; $CDCl_3$) of 2,2-dimethylpentan-3-one

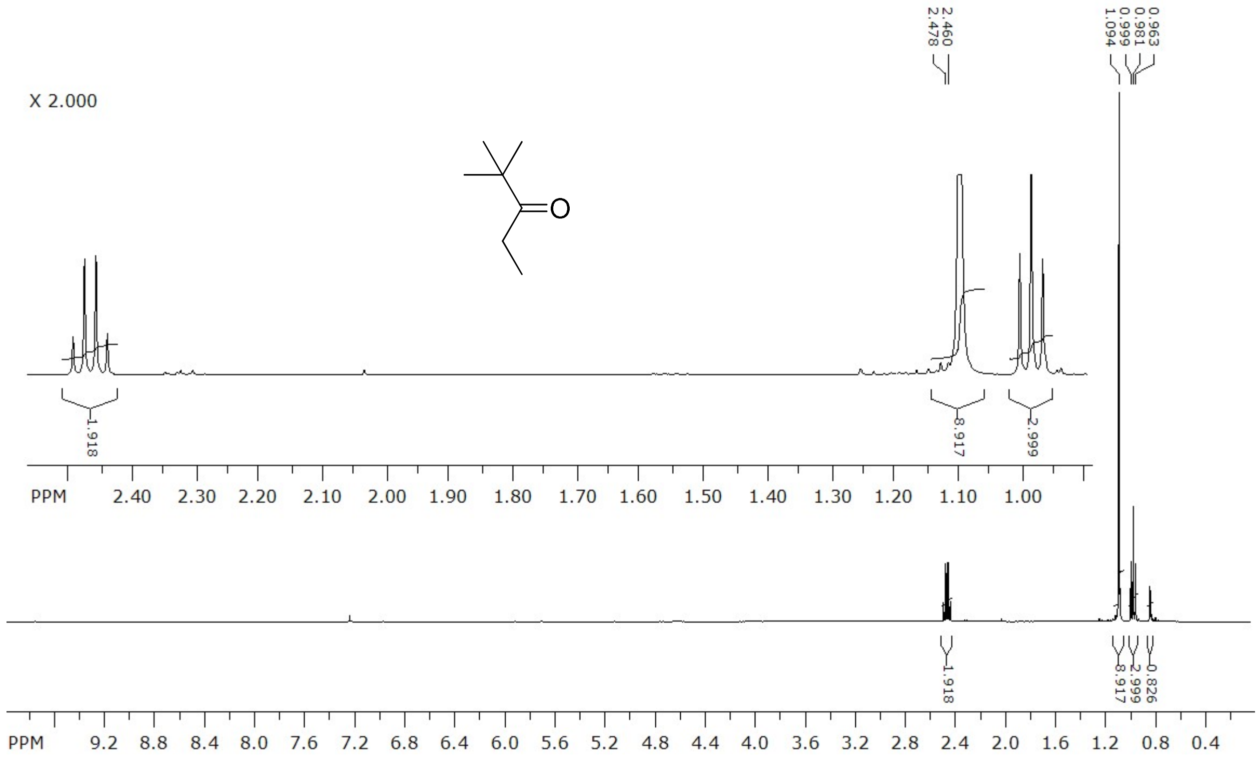

Figure S3. ^{1}H NMR (500 MHz; $CDCl_3$) of (2R(S),3R(S),4R(S),5S(R))-2,5-di-*tert*-butyl-3,4-bis(methoxycarbonyl)-2-ethylpyrrolidine (**8**)

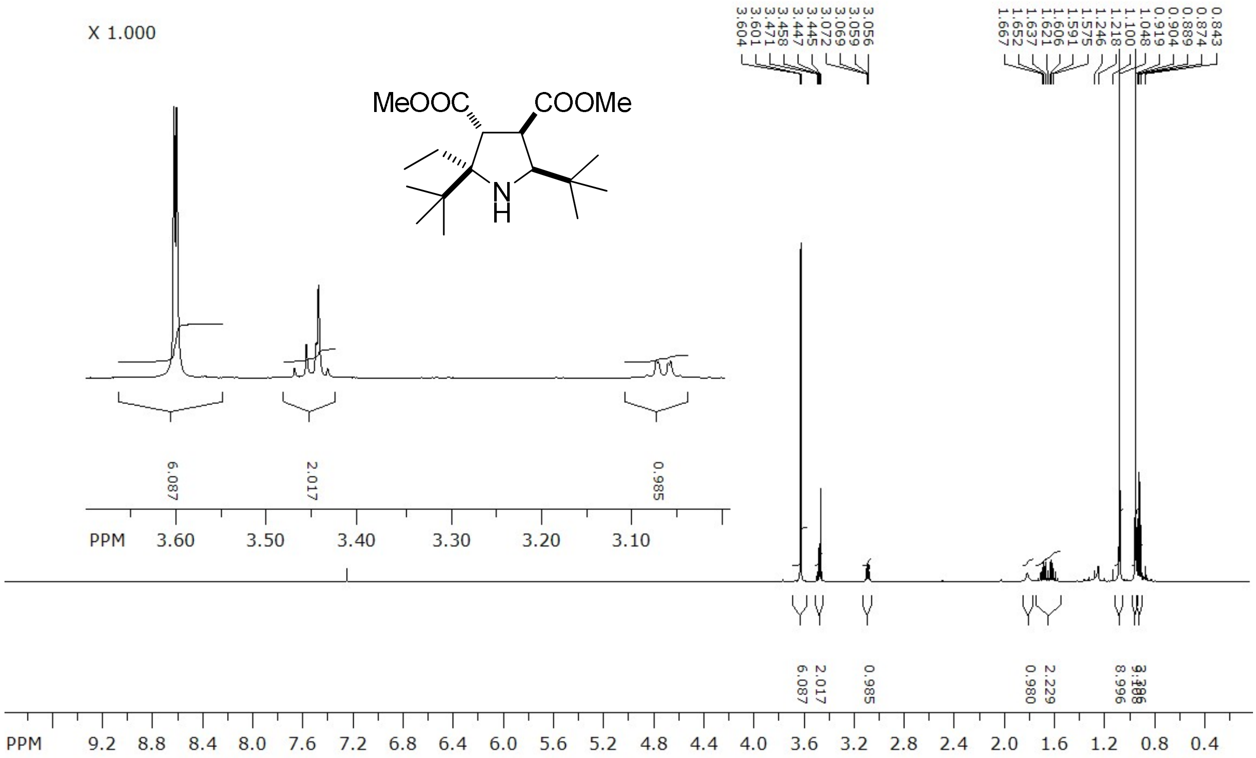

Figure S4. ^{13}C NMR (125 MHz; $CDCl_3$) of (2R(S),3R(S),4R(S),5S(R))-2,5-di-*tert*-butyl-3,4-bis(methoxycarbonyl)-2-ethylpyrrolidine (**8**)

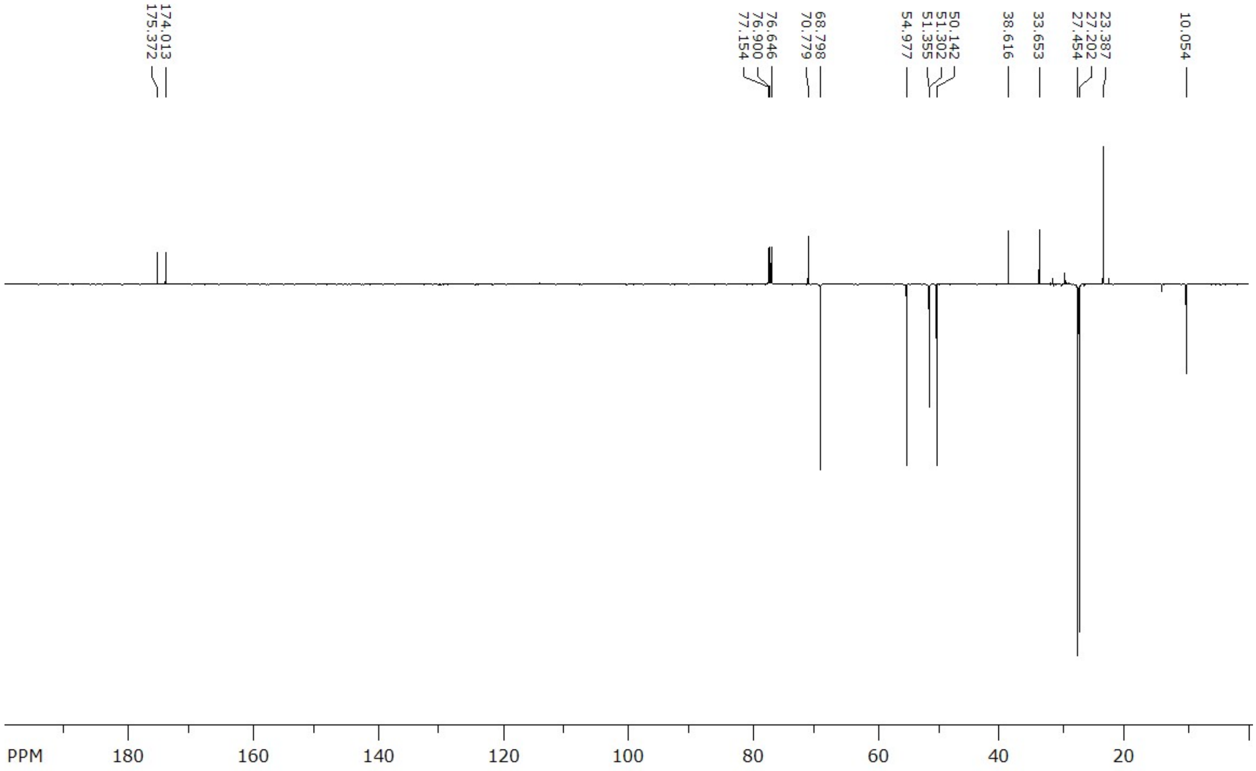

Figure S5. ^{1}H NMR (400 MHz; $(CD_3)_2CO$; CF_3COOH) of (2R(S),3R(S),4R(S),5S(R))-2,5-di-*tert*-butyl-3,4-bis(methoxycarbonyl)-2-ethylpyrrolidine (**8**)

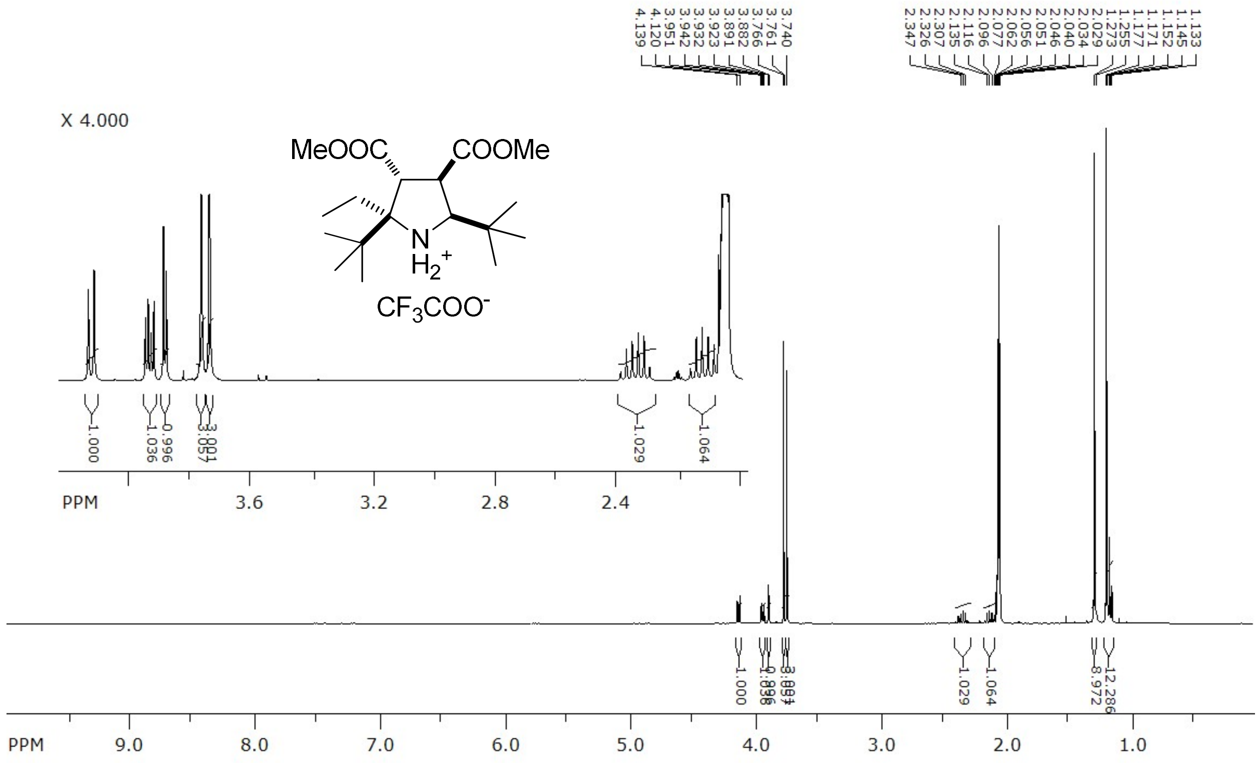

Figure S6. ^{13}C NMR (100 MHz; $(CD_3)_2CO$; CF_3COOH) of (2R(S),3R(S),4R(S),5S(R))-2,5-di-tert-butyl-3,4-bis(methoxycarbonyl)-2-ethylpyrrolidine (**8**)

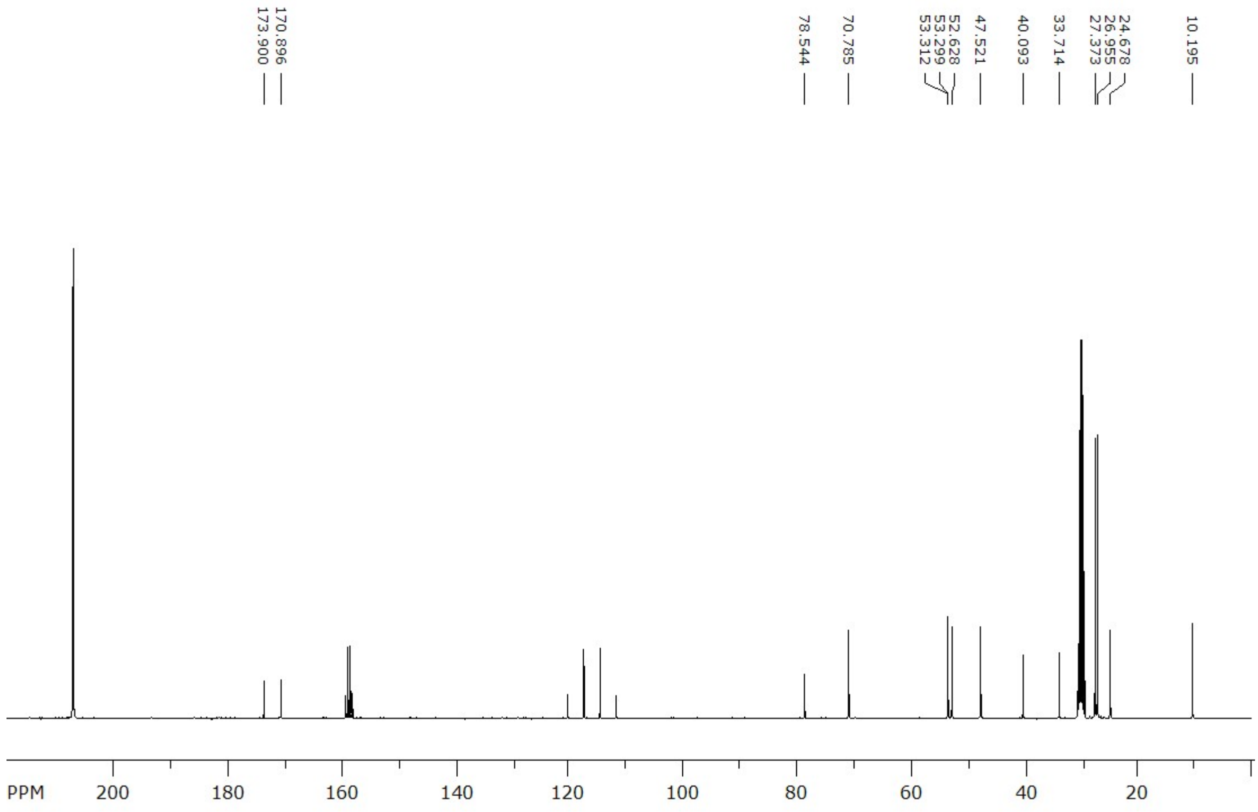

Figure S7. ^{1}H NMR (500 MHz; $CDCl_3$) of (2S(R),3R(S),4R(S),5R(S))-2,5-di-*tert*-butyl-3,4-bis(methoxycarbonyl)-2-ethylpyrrolidine (**9**)

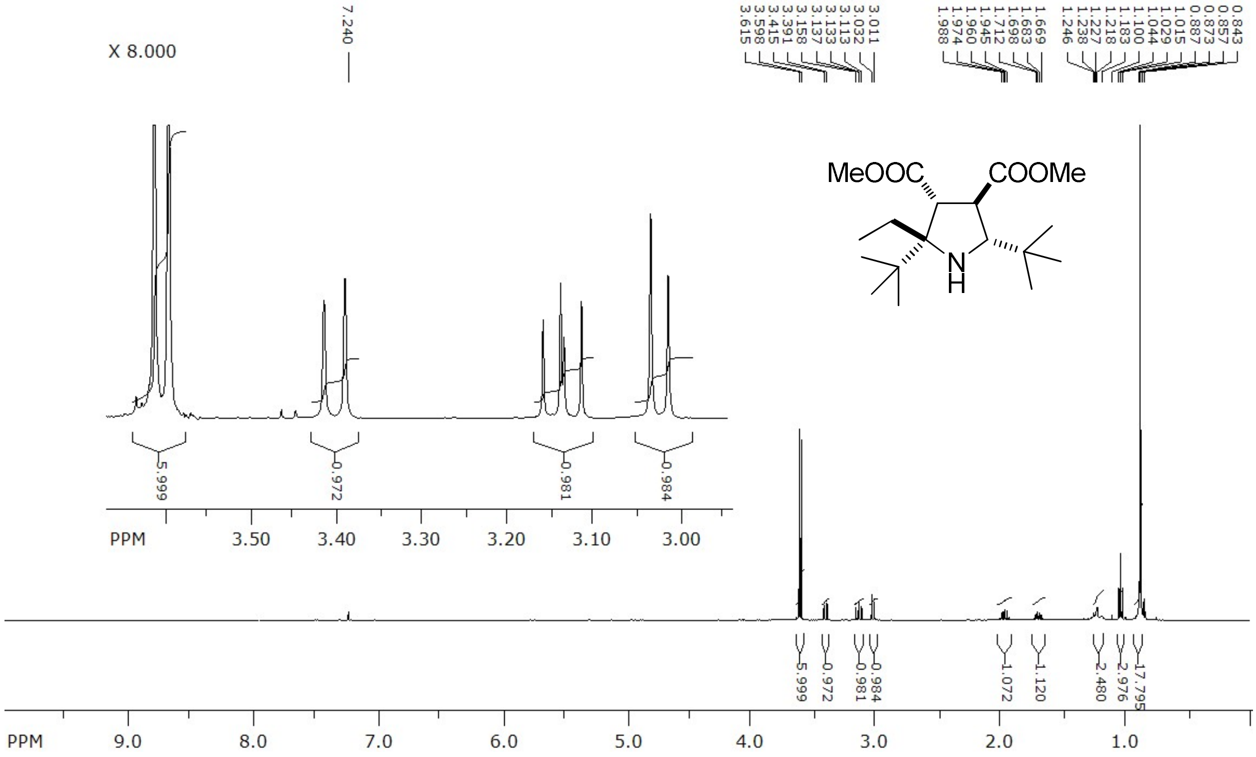

Figure S8. ^{13}C NMR (125 MHz; $CDCl_3$) of (2S(R),3R(S),4R(S),5R(S))-2,5-di-*tert*-butyl-3,4-bis(methoxycarbonyl)-2-ethylpyrrolidine (**9**)

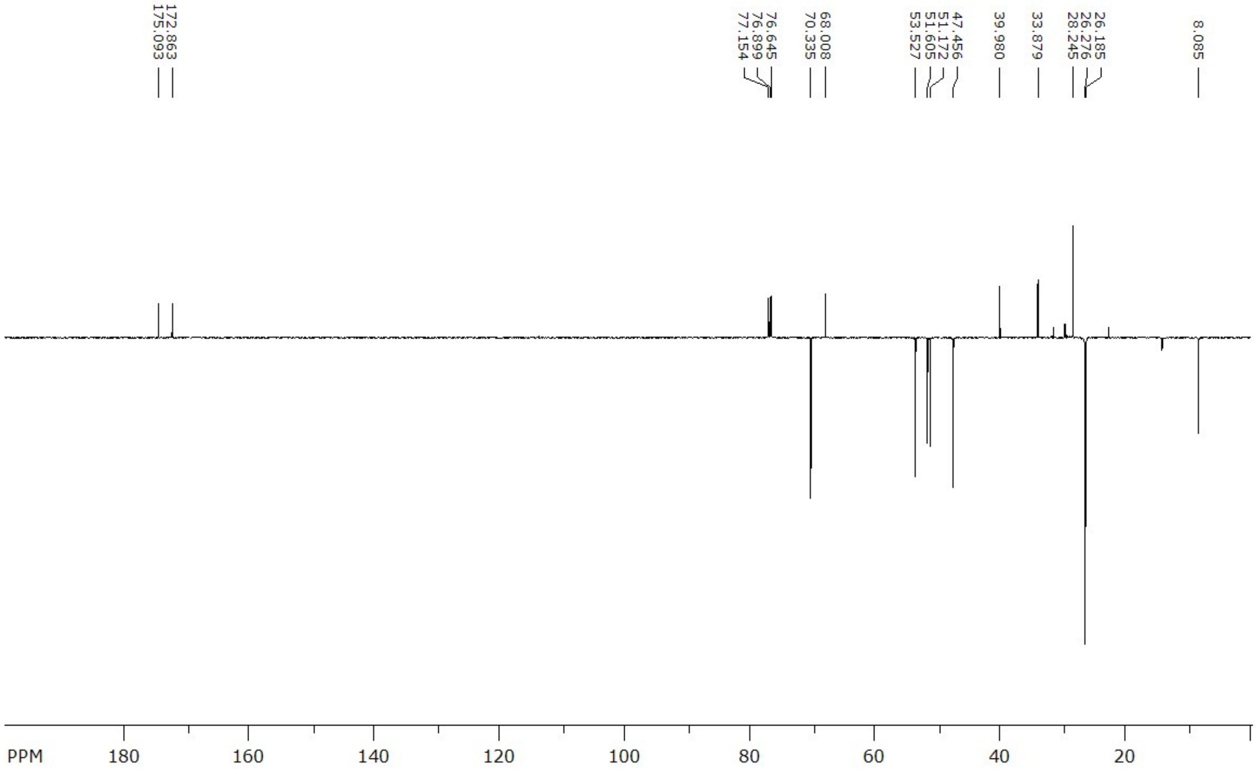

Figure S9. ^{1}H NMR (500 MHz; $CDCl_3$) of dimethyl 2-(1-((2,2-dimethylpentan-3-ylidene)amino)-2,2-dimethylpropyl)succinate (**10**)

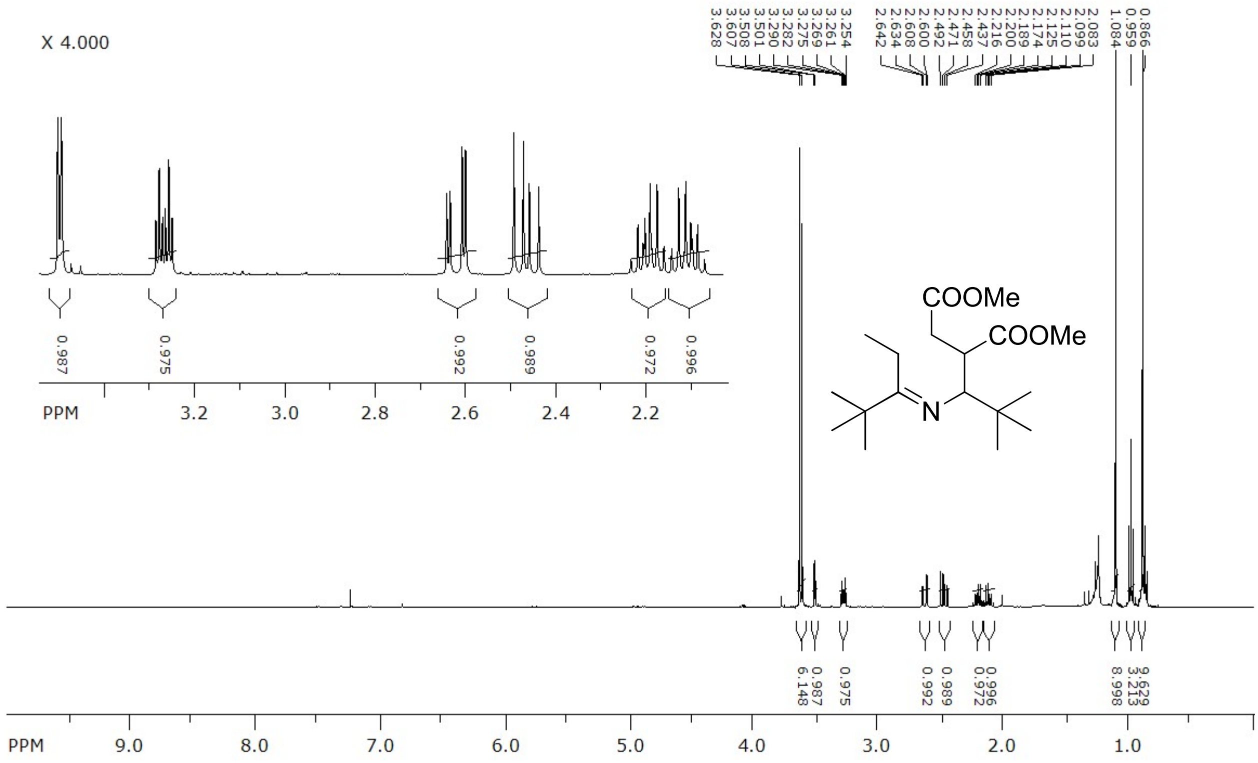

Figure S10. ^{13}C NMR (125 MHz; $CDCl_3$) of dimethyl 2-(1-((2,2-dimethylpentan-3-ylidene)amino)-2,2-dimethylpropyl)succinate (**10**)

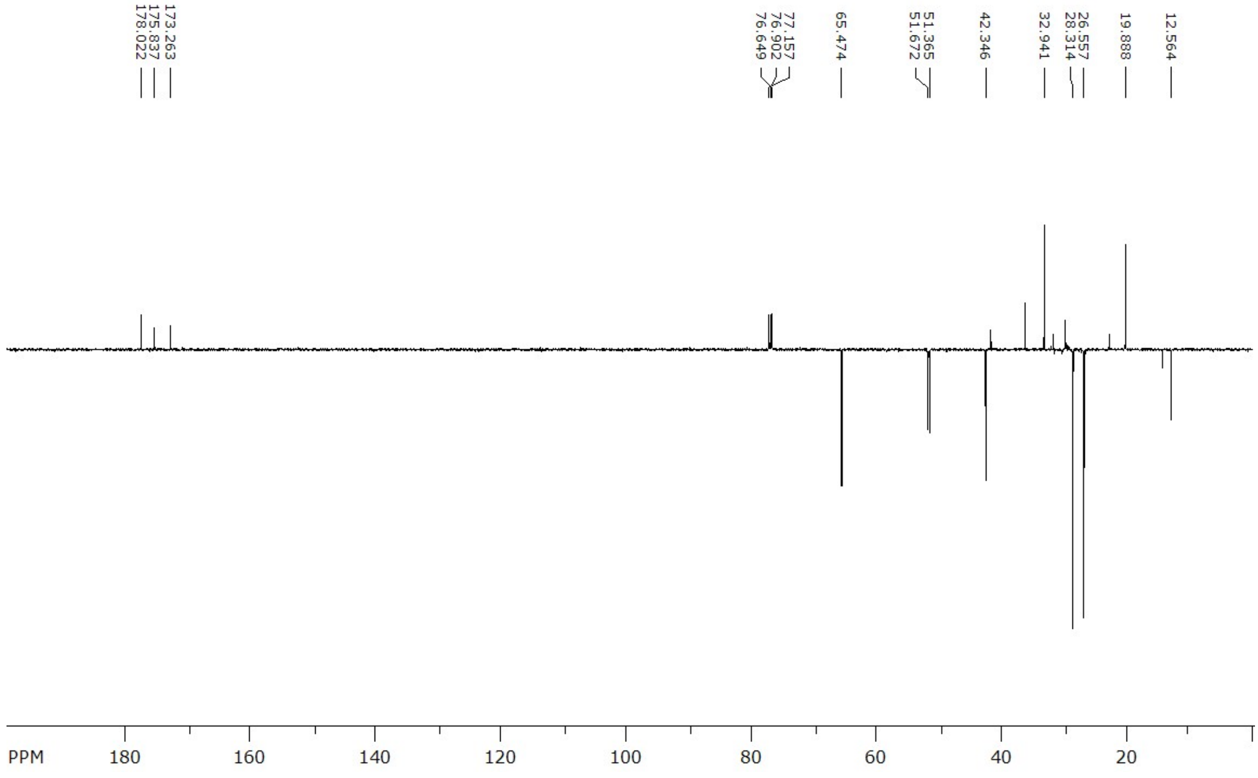

Figure S11. ^{1}H NMR (500 MHz; $CDCl_3$) of (2R(S),3R(S),4R(S),5S(R))-2,5-di-*tert*-butyl-3,4-bis(hydroxymethyl)-2-ethylpyrrolidine (**11**)

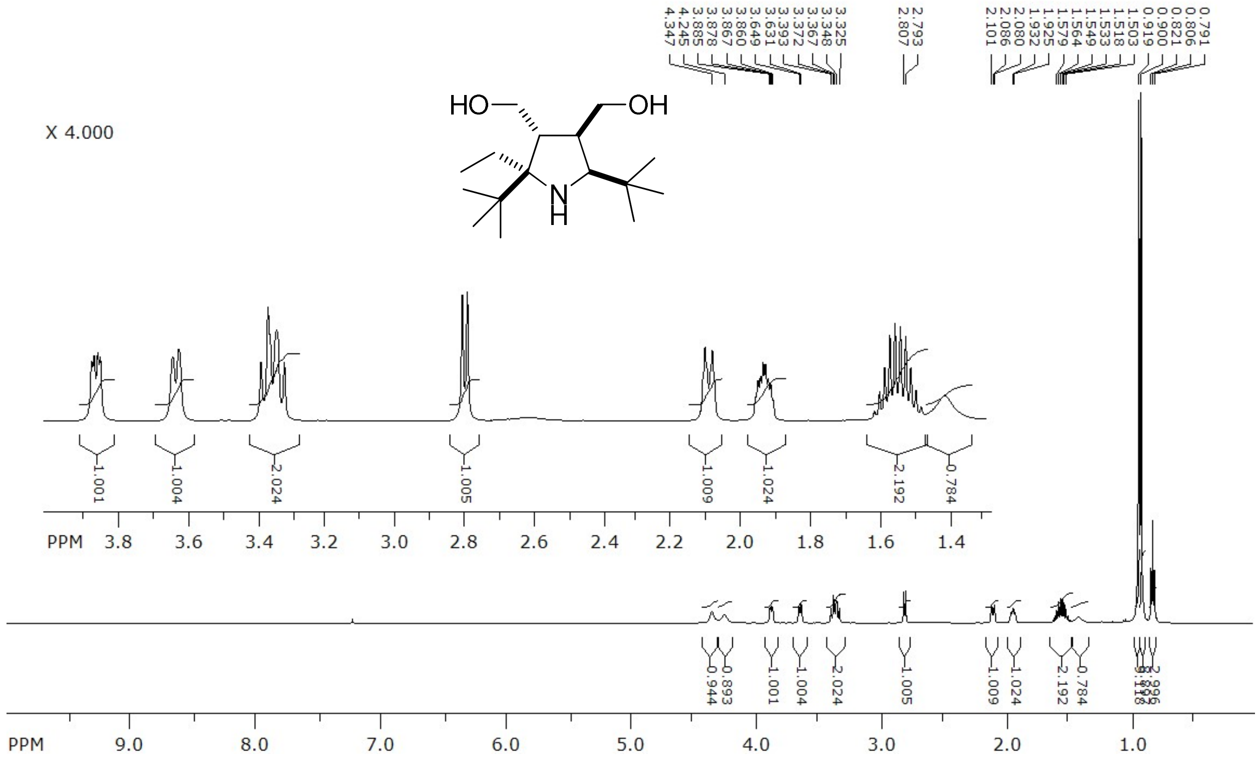

Figure S12. ^{13}C NMR (125 MHz; $CDCl_3$) of (2R(S),3R(S),4R(S),5S(R))-2,5-di-tert-butyl-3,4-bis(hydroxymethyl)-2-ethylpyrrolidine (**11**)

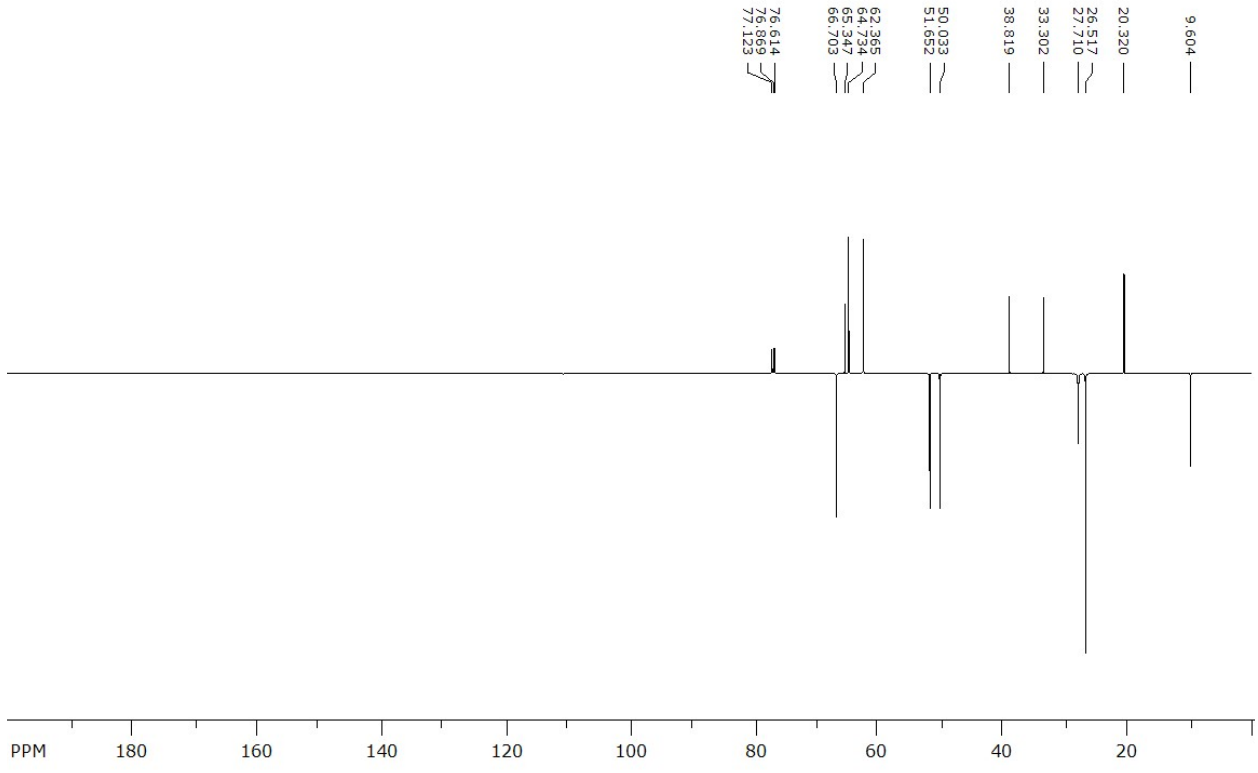

Figure S13. ^{1}H NMR (400 MHz, $CDCl_3$) of (2R(S),3R(S),4R(S))-2,5-di-*tert*-butyl-2-ethyl-3,4-bis(hydroxymethyl)-3,4-dihydro-2*H*-pyrrole 1-oxide (**12**)

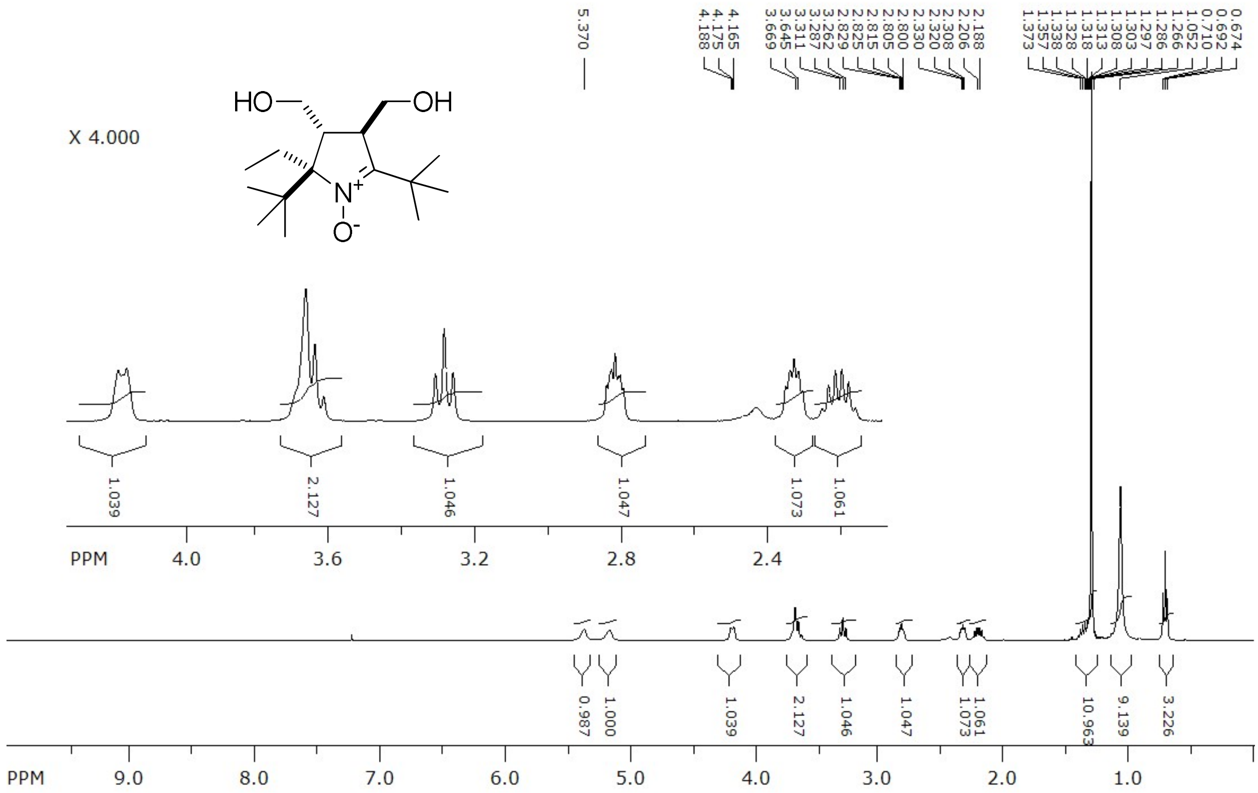

Figure S14. ^{13}C NMR (100 MHz, $CDCl_3$) of (2R(S),3R(S),4R(S))-2,5-di-*tert*-butyl-2-ethyl-3,4-bis(hydroxymethyl)-3,4-dihydro-2*H*-pyrrole 1-oxide (**12**)

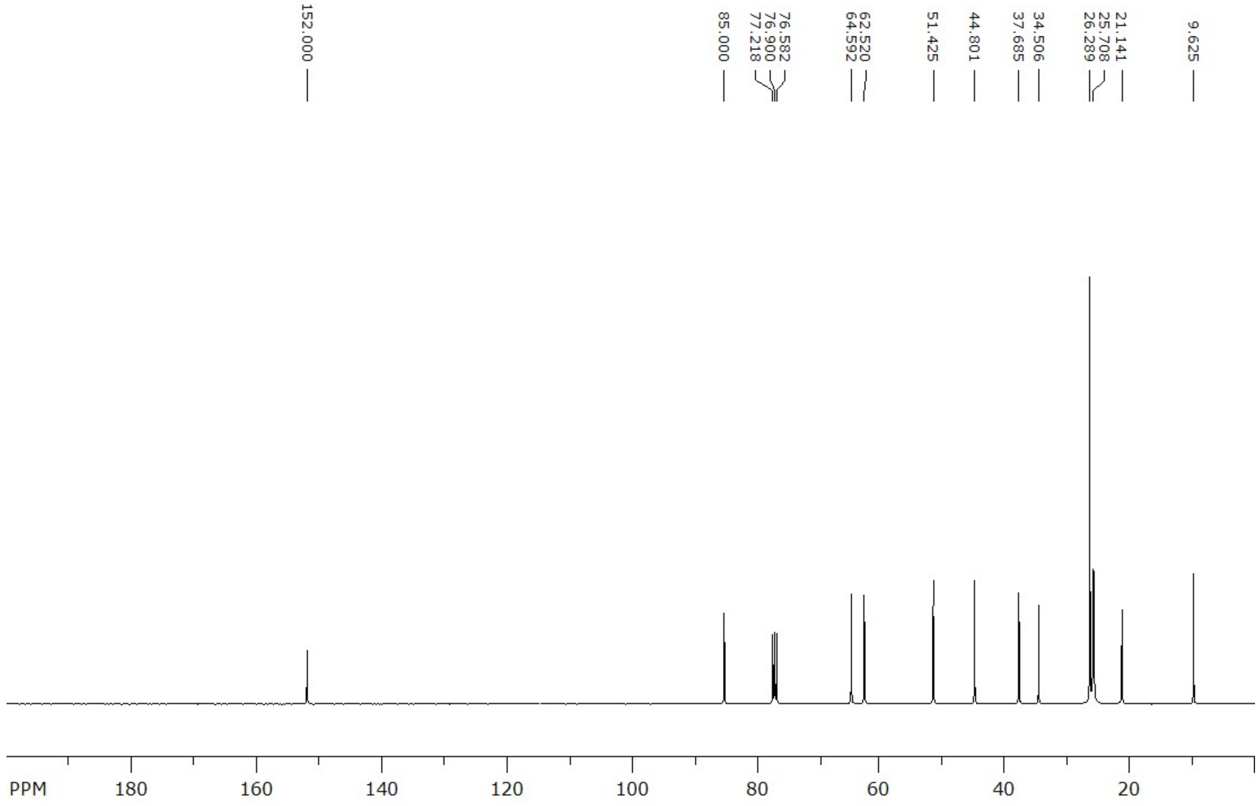

Figure S15. ^{1}H NMR (500 MHz, $CDCl_3$) of (2R(S),3R(S),4R(S))-2,5-di-*tert*-butyl-2-ethyl-3,4-bis(((2-methoxypropan-2-yl)oxy)methyl)-3,4-dihydro-2*H*-pyrrole 1-oxide (**14**)

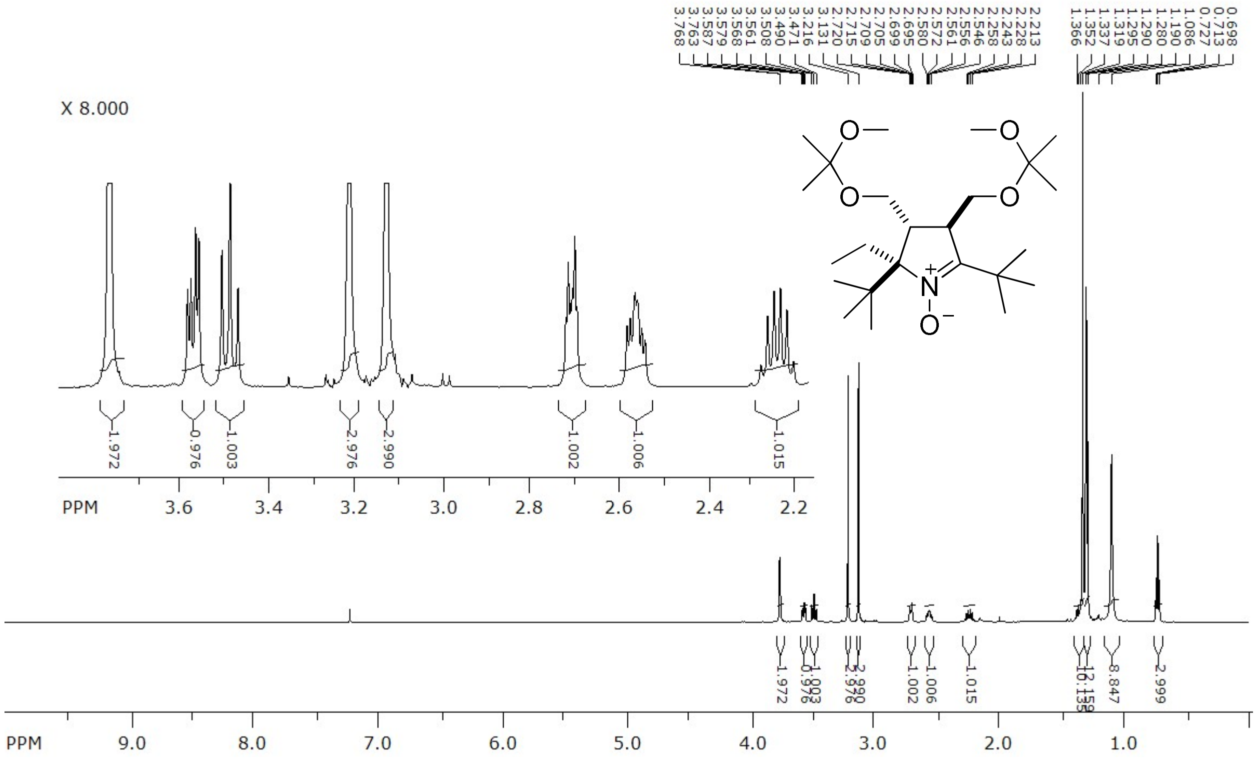

Figure S16. ^{13}C NMR (125 MHz, $CDCl_3$) of (2R(S),3R(S),4R(S))-2,5-di-tert-butyl-2-ethyl-3,4-bis(((2-methoxypropan-2-yl)oxy)methyl)-3,4-dihydro-2*H*-pyrrole 1-oxide (**14**)

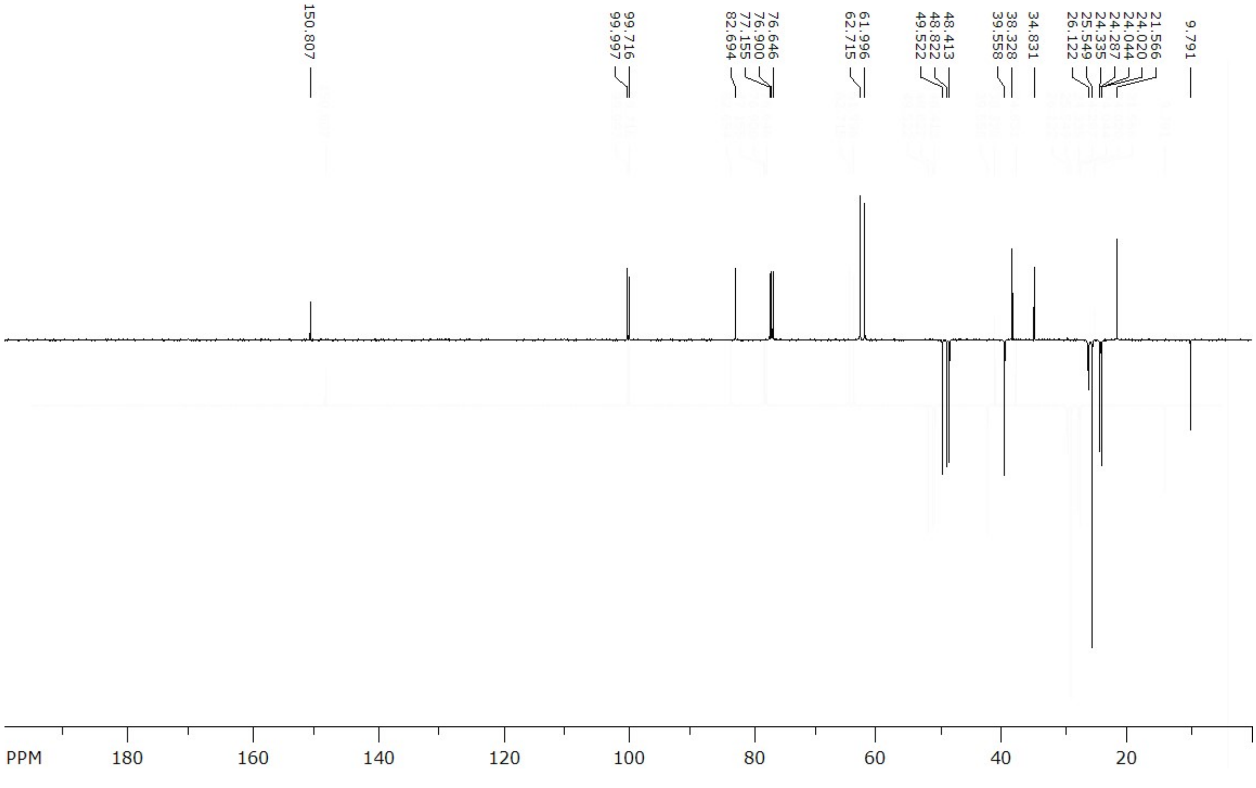

Figure S17. ^{1}H NMR (400 MHz; CD_3OD, Zn/CF_3COOH system) of (2R(S),3R(S),4R(S),5S(R))-2,2,5-triethyl-5-*tert*-butyl-3,4-bis(hydroxymethyl)-pyrrolidine-1-oxyl (

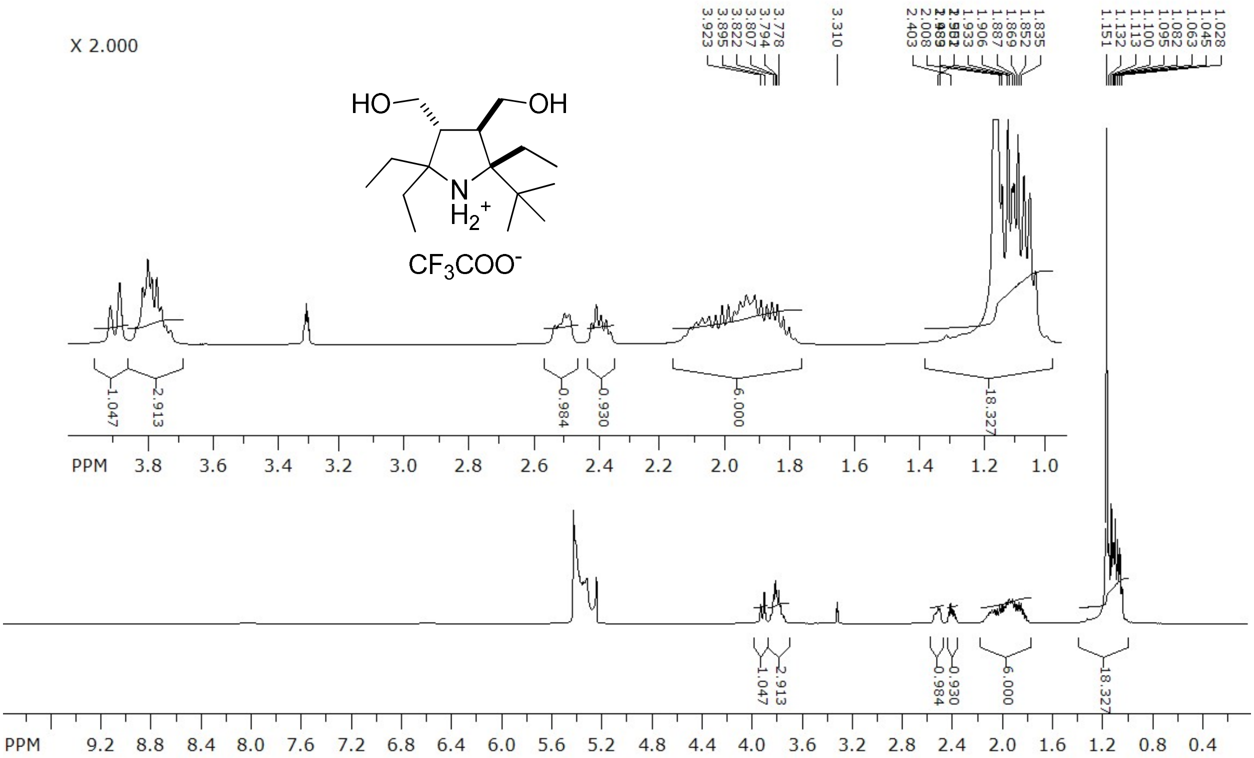

Figure S18. ^{13}C NMR (100 MHz; CD_3OD, Zn/CF_3COOH system) of (2R(S),3R(S),4R(S),5S(R))-2,2,5-triethyl-5-*tert*-butyl-3,4-bis(hydroxymethyl)-pyrrolidine-1-oxyl (

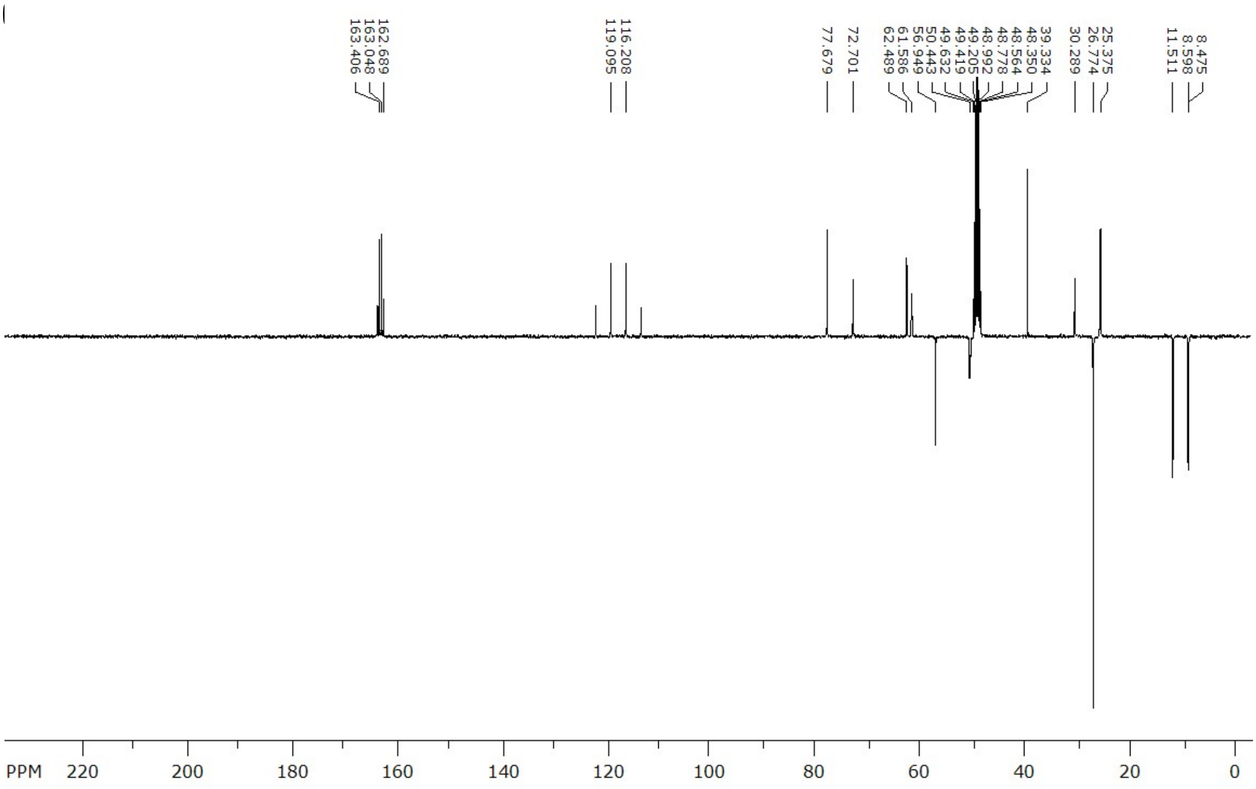

Figure S19. ^{1}H NMR (400 MHz; $CDCl_3$) of (1S(R),4R(S),5R(S))-2,4-di-*tert*-butyl-4-ethyl-1-propyl-3-azabicyclo[3.1.0]hex-2-ene (**15**)

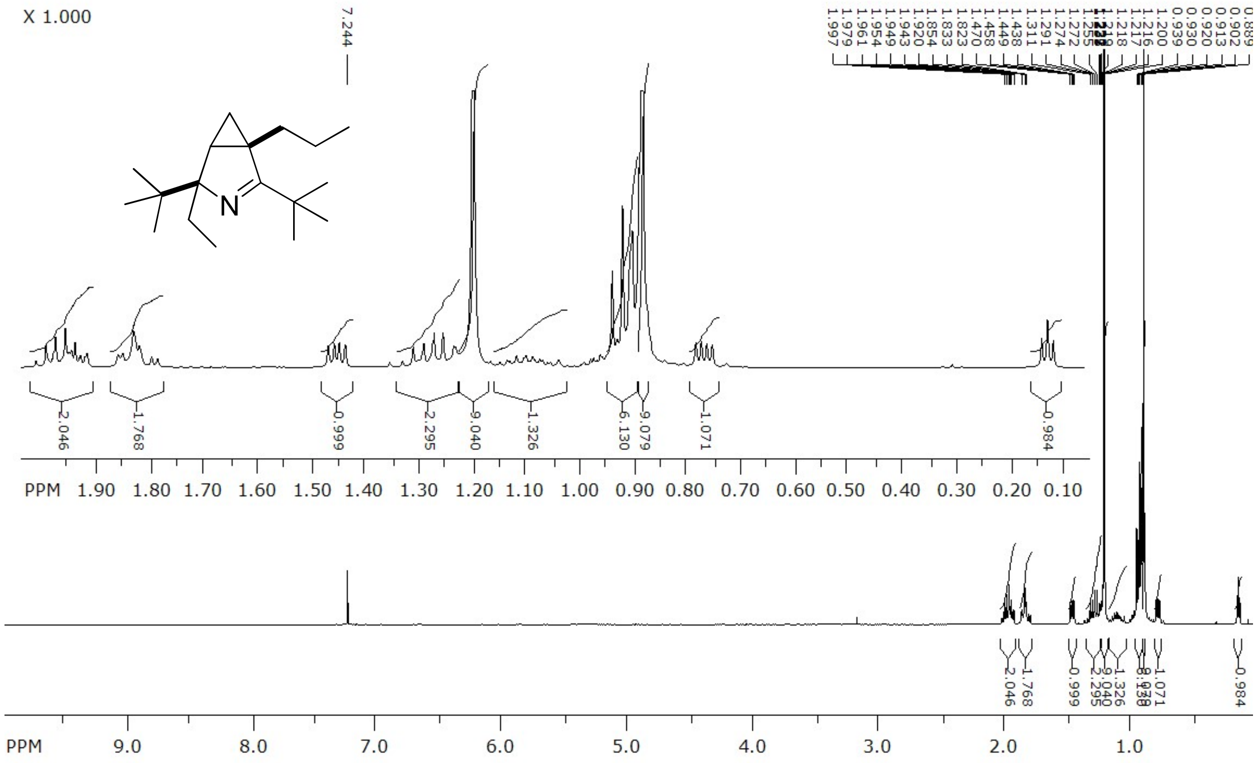

Figure S20. ^{13}C NMR (100 MHz; $CDCl_3$) of (1S(R),4R(S),5R(S))-2,4-di-*tert*-butyl-4-ethyl-1-propyl-3-azabicyclo[3.1.0]hex-2-ene (**15**)

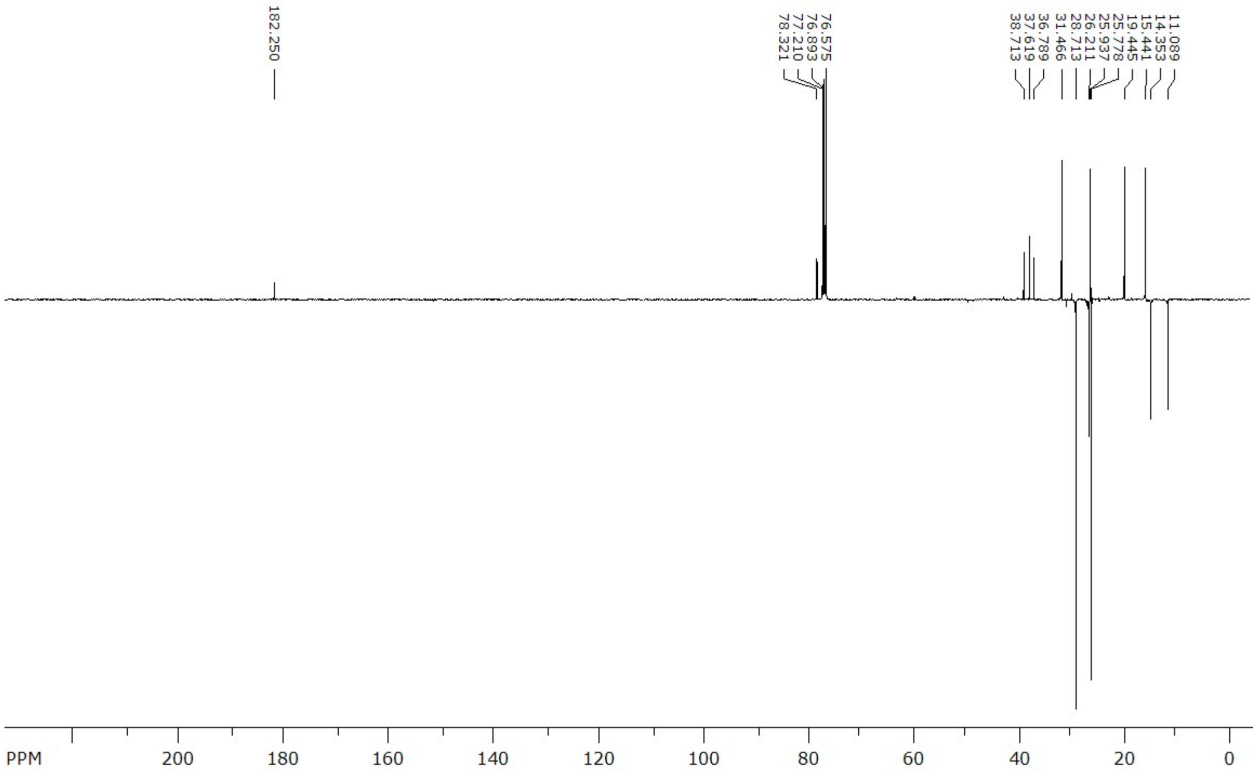

Figure S21. ^{1}H NMR (500 MHz; $CDCl_3$) of (2S(R),3R(S),4R(S))-2,5-di-*tert*-butyl-2-ethyl-3,4-bis(methoxycarbonyl)-3,4-dihydro-2H-pyrrole 1-oxide (**16**)

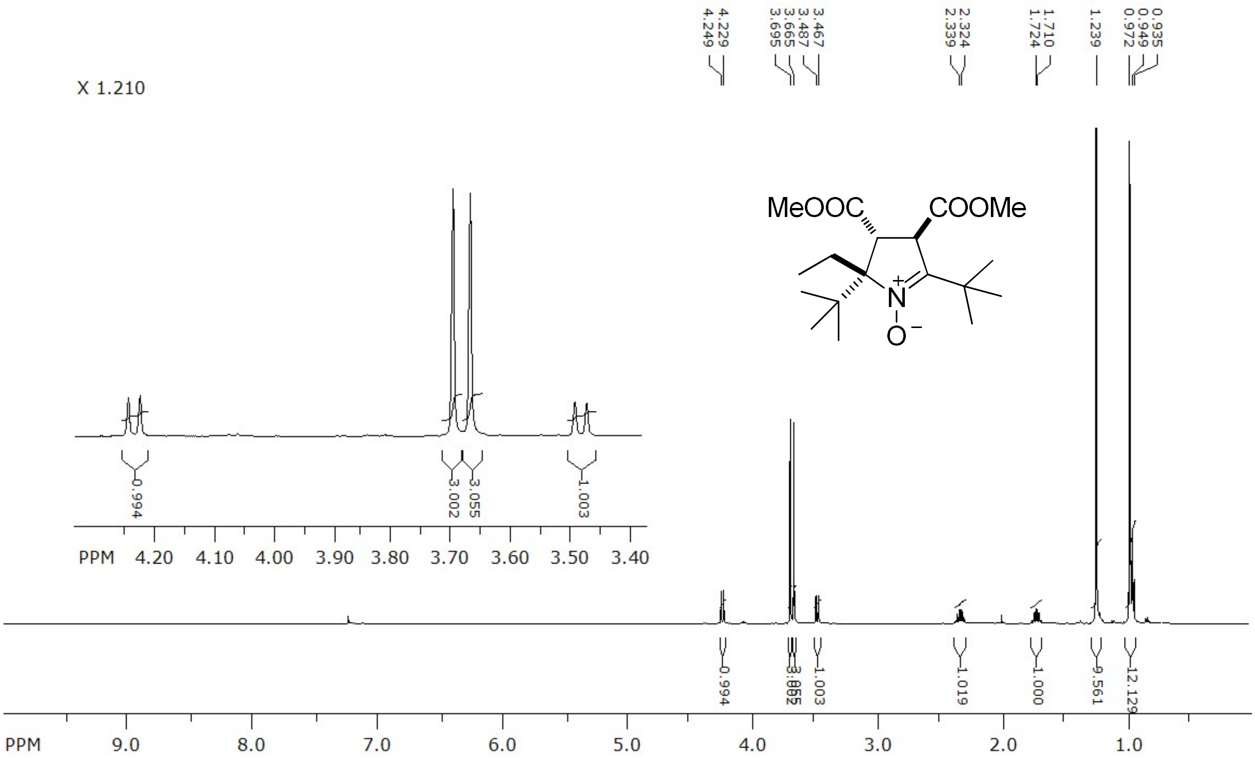

Figure S22. ^{13}C NMR (125 MHz; $CDCl_3$) of (2S(R),3R(S),4R(S))-2,5-di-*tert*-butyl-2-ethyl-3,4-bis(methoxycarbonyl)-3,4-dihydro-2H-pyrrole 1-oxide (**16**)

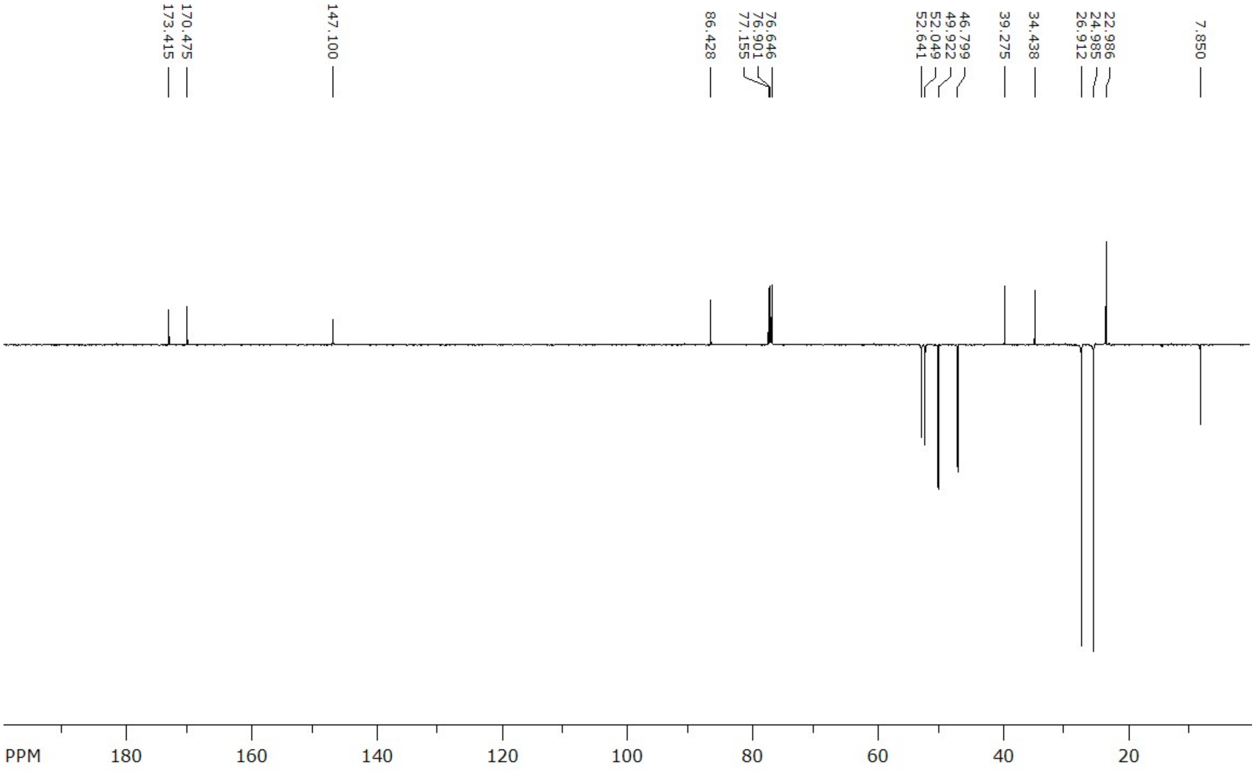

Figure S23. ^{1}H NMR (400 MHz; $CDCl_3$) of (2S(R),3R(S))-2,5-di-*tert*-butyl-2-ethyl-3-(methoxycarbonyl)-3,4-dihydro-2H-pyrrole 1-oxide (**17**)

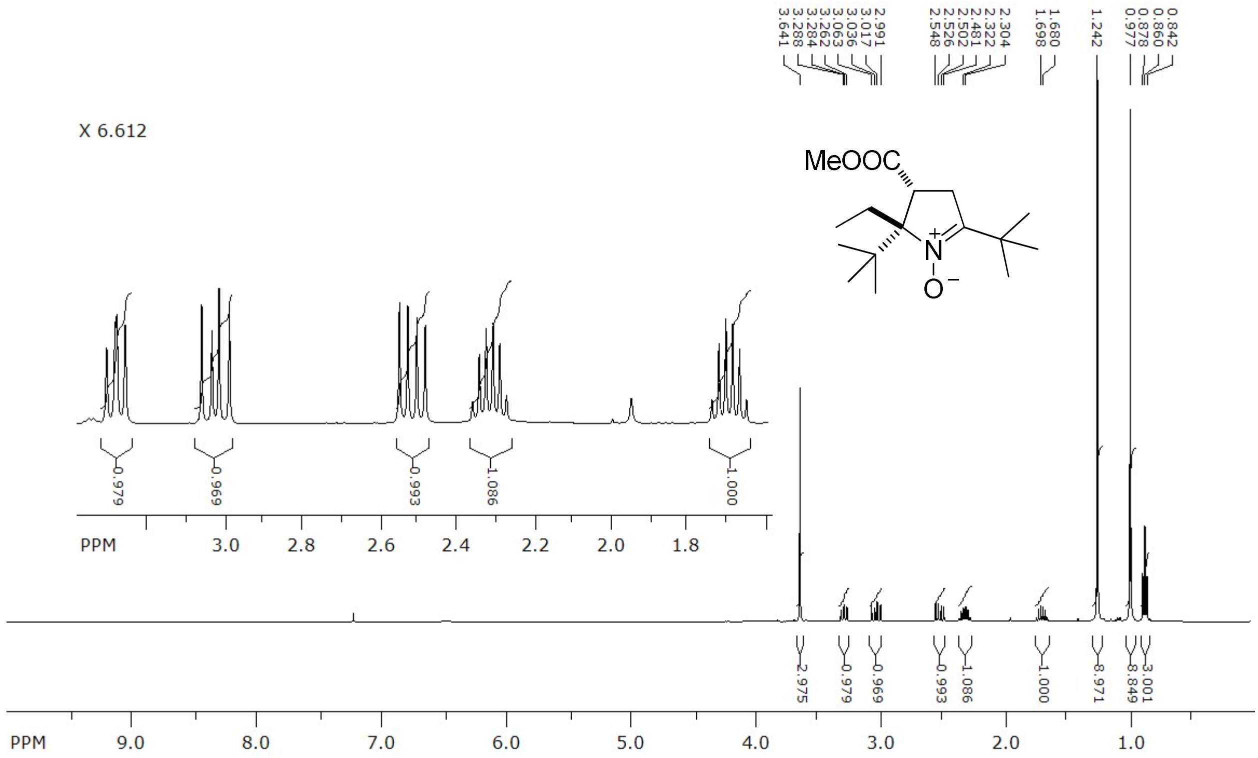

Figure S24. ^{13}C NMR (100 MHz; $CDCl_3$) of (2S(R),3R(S))-2,5-di-*tert*-butyl-2-ethyl-3-(methoxycarbonyl)-3,4-dihydro-2H-pyrrole 1-oxide (**17**)

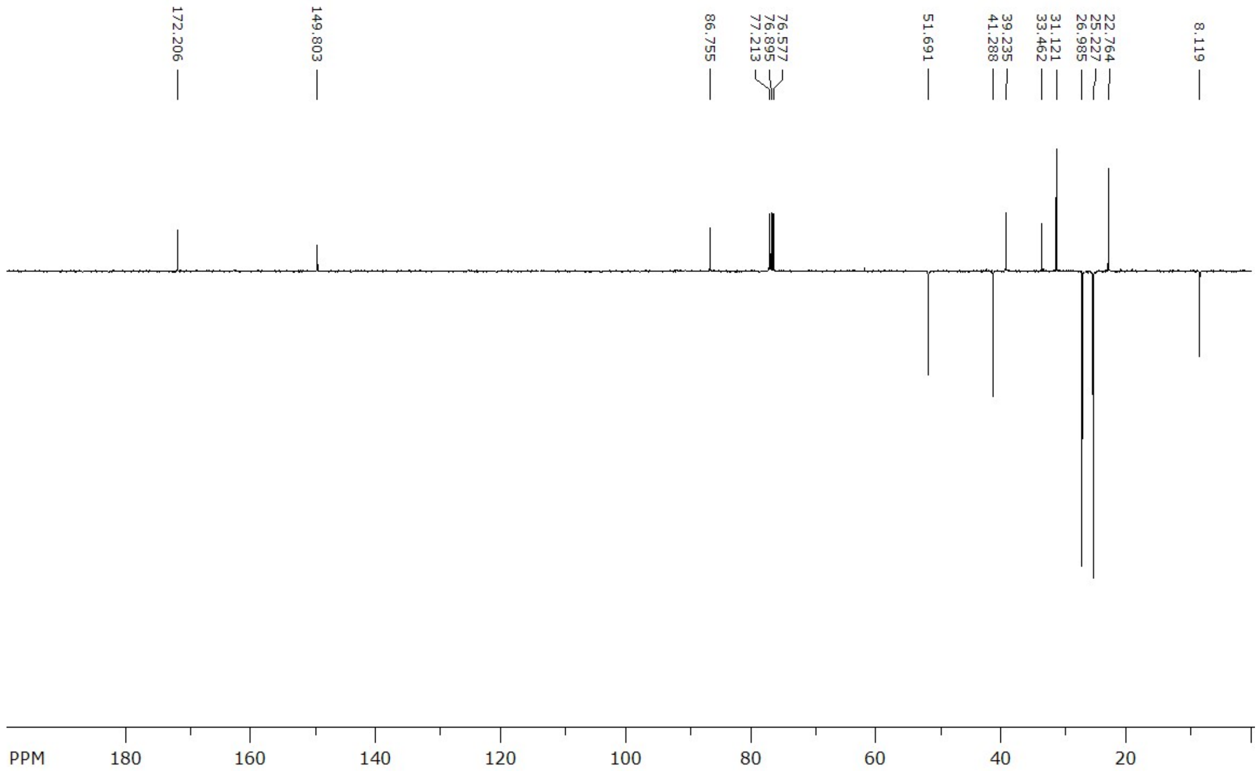

Figure S25. ^{1}H NMR (500 MHz; $CDCl_3$) of (2S(R),3S(R))-2,5-di-*tert*-butyl-2-ethyl-3-(methoxycarbonyl)-3,4-dihydro-2H-pyrrole 1-oxide (**18**)

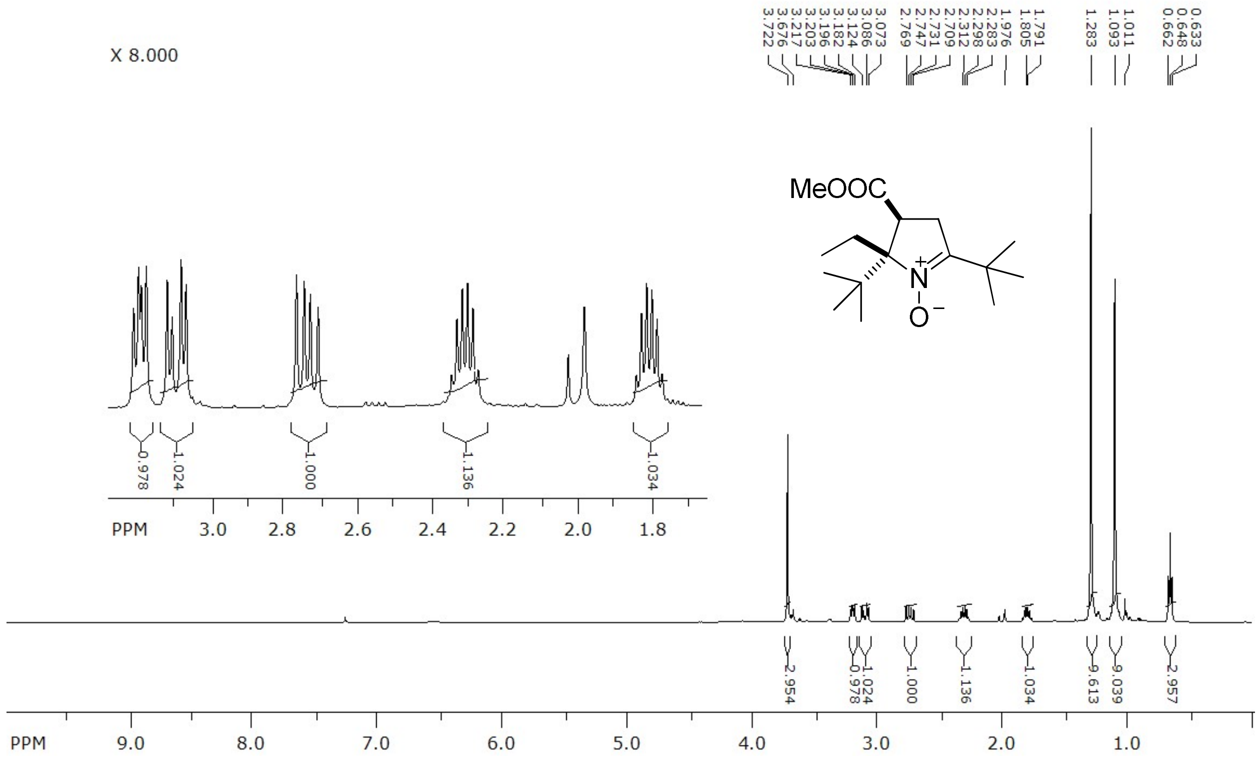

Figure S26. ^{13}C NMR (125 MHz; $CDCl_3$) of (2S(R),3S(R))-2,5-di-*tert*-butyl-2-ethyl-3-(methoxycarbonyl)-3,4-dihydro-2H-pyrrole 1-oxide (**18**)

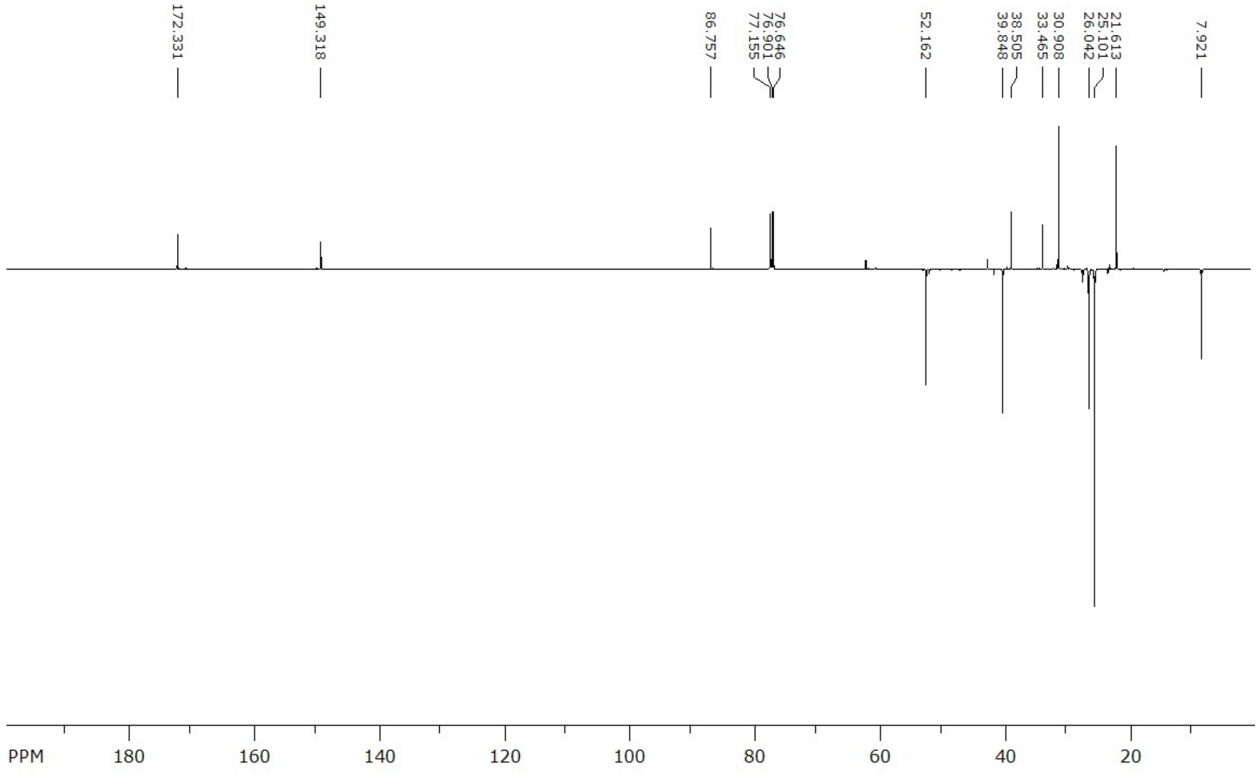

Figure S27. ^{1}H NMR (600 MHz; $CDCl_3$) of (2S(R),3R(S))-2,5-di-*tert*-butyl-2-ethyl-3-(hydroxymethyl)-3,4-dihydro-2H-pyrrole 1-oxide (**20**)

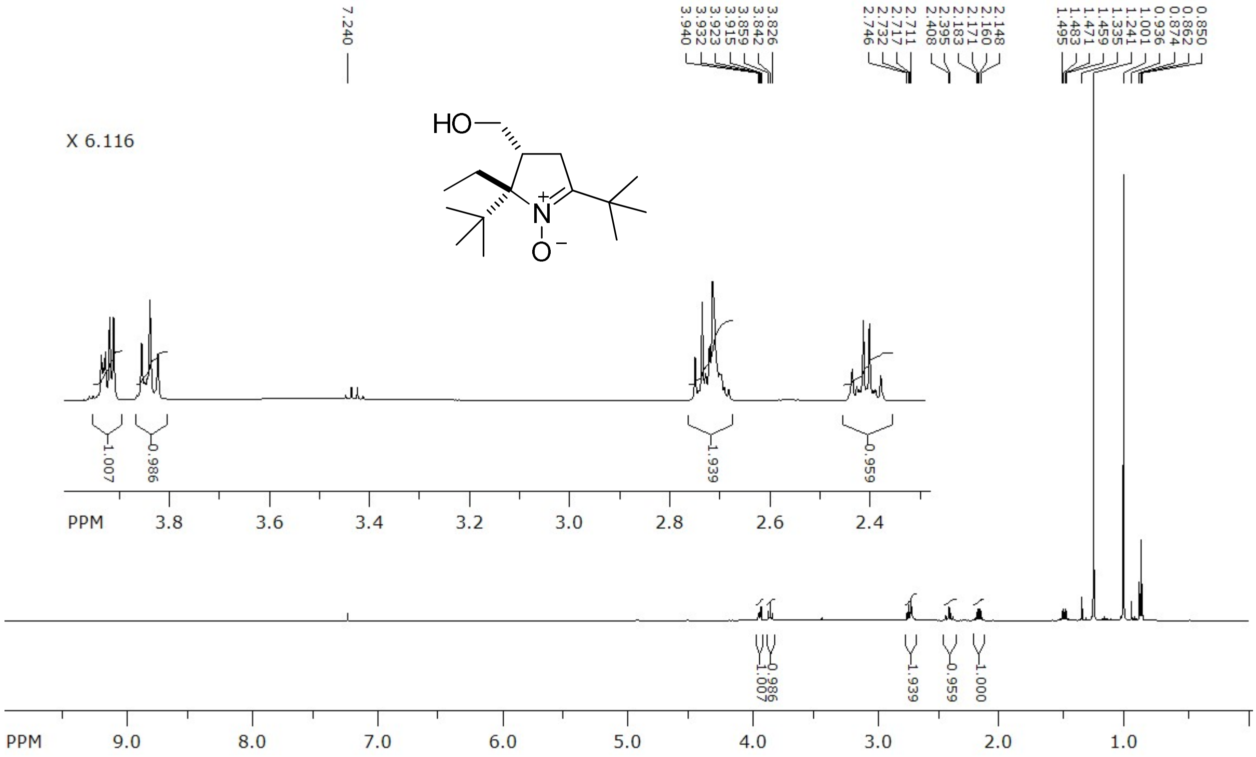

Figure S28. ^{13}C NMR (150 MHz; $CDCl_3$) of (2S(R),3R(S))-2,5-di-*tert*-butyl-2-ethyl-3-(hydroxymethyl)-3,4-dihydro-2H-pyrrole 1-oxide (**20**)

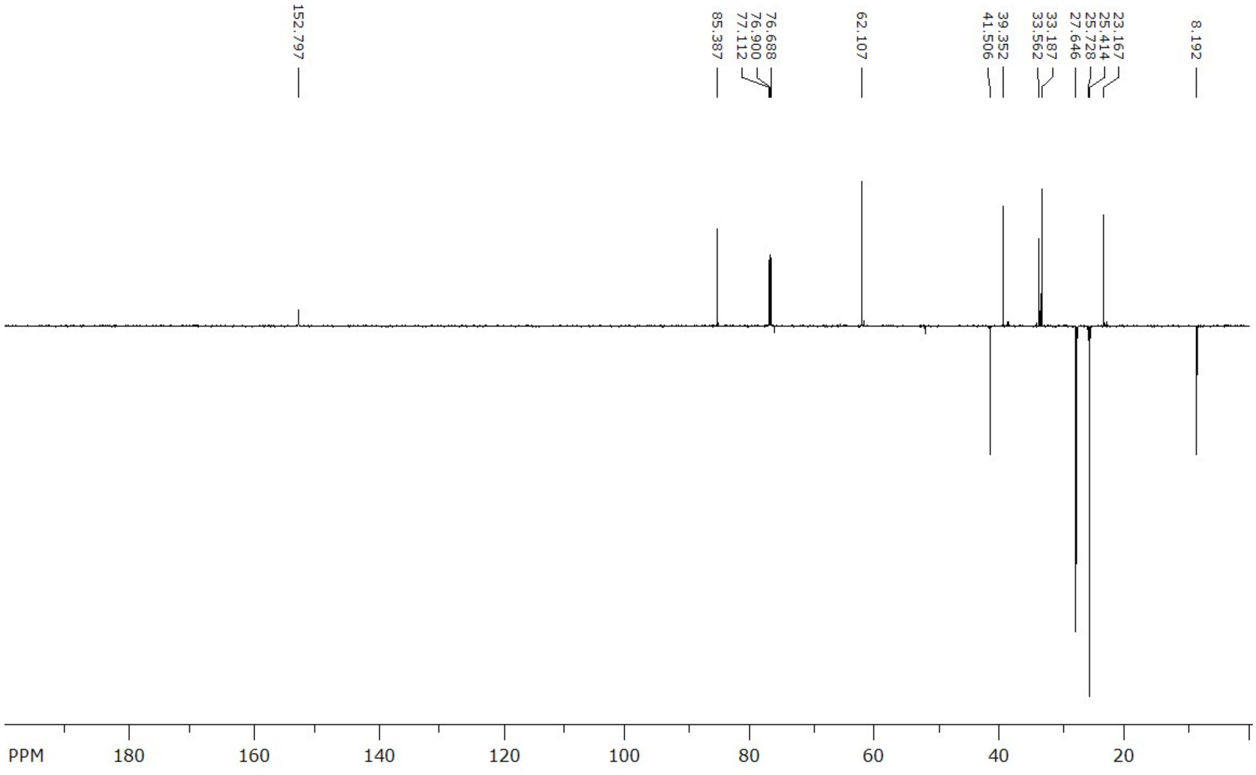

Figure S29. ^{1}H NMR (400 MHz; $CDCl_3$) of (2S(R),3S(R))-2,5-di-*tert*-butyl-2-ethyl-3-(hydroxymethyl)-3,4-dihydro-2H-pyrrole 1-oxide (**21**)

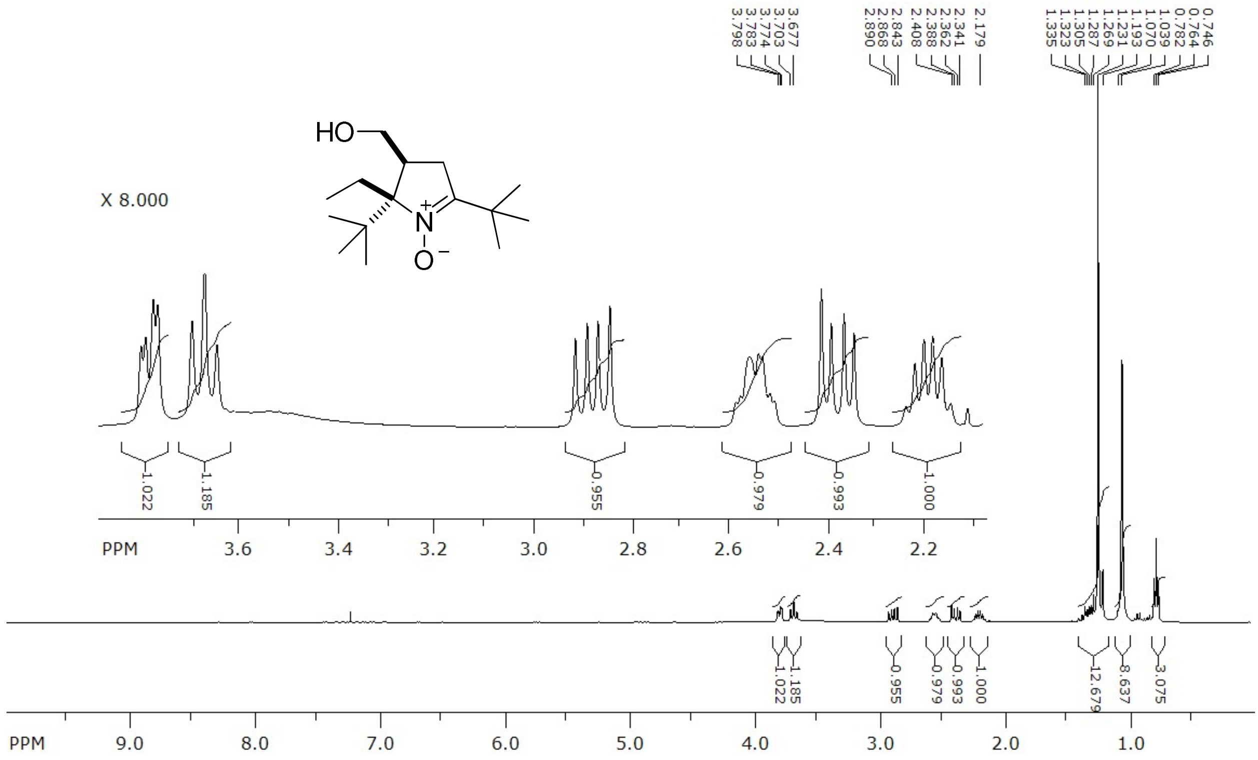

Figure S30. ^{13}C NMR (125 MHz; $CDCl_3$) of (2S(R),3S(R))-2,5-di-*tert*-butyl-2-ethyl-3-(hydroxymethyl)-3,4-dihydro-2H-pyrrole 1-oxide (**21**)

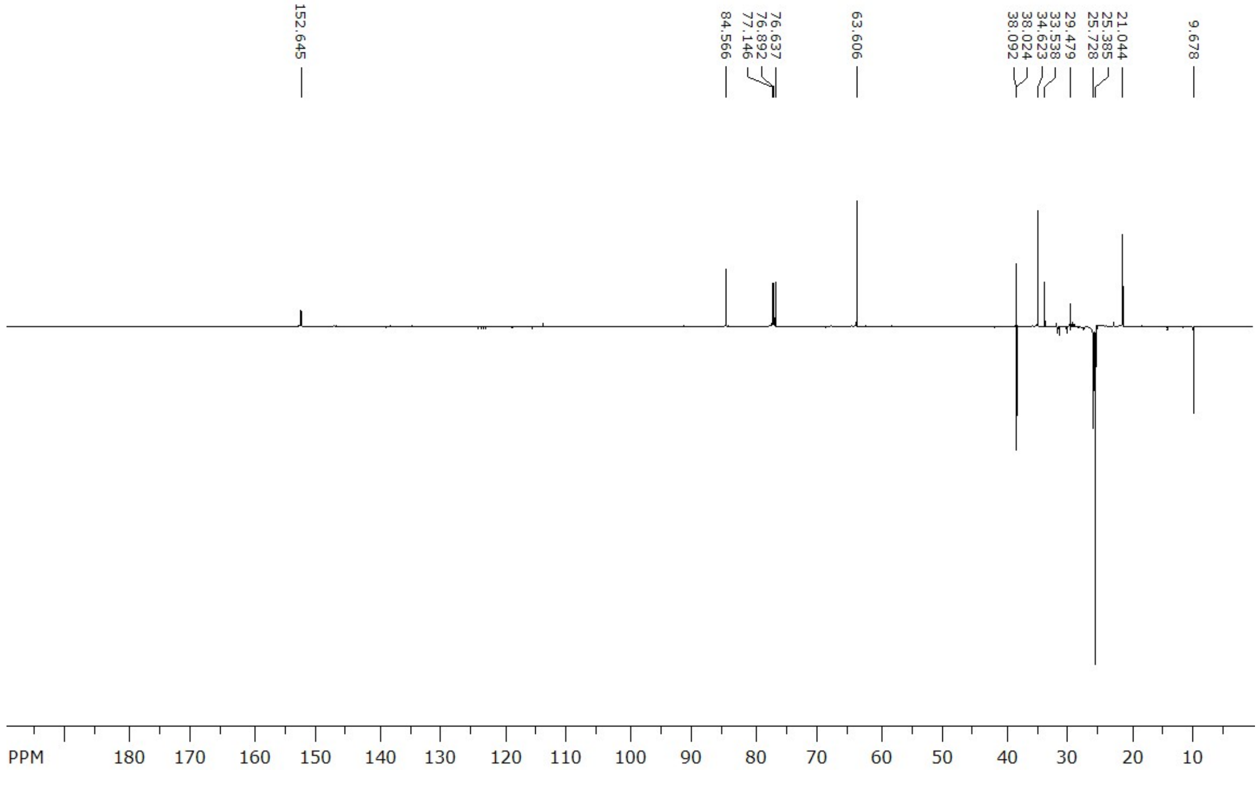

Figure S31. ^{1}H NMR (400 MHz; CD_3OD, Zn/CF_3COOH system) of (2S(R),3R(S))-2,5-di-tert-butyl-2,5-diethyl-3-(hydroxymethyl)pyrrolidin-1-oxyl (**23**)

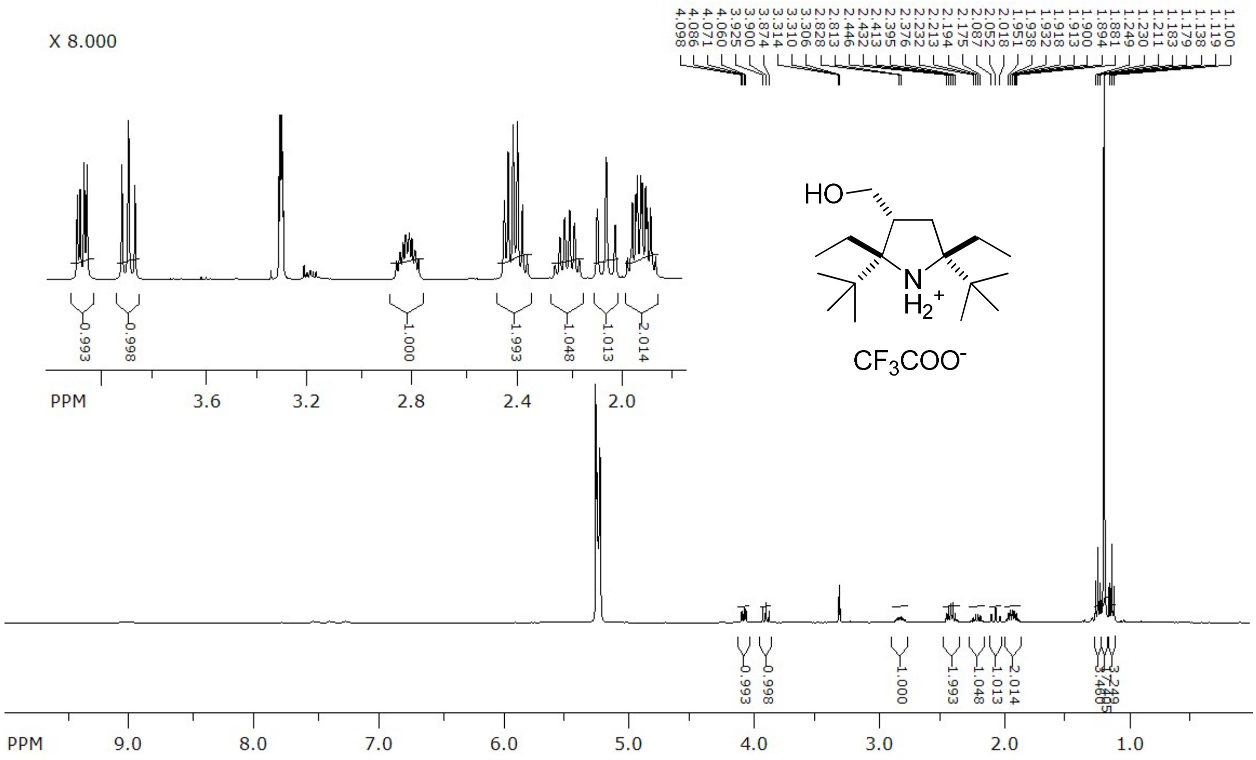

Figure S32. ^{13}C NMR (75 MHz; CD_3OD, Zn/CF_3COOH system) of (2S(R),3R(S))-2,5-di-tert-butyl-2,5-diethyl-3-(hydroxymethyl)pyrrolidin-1-oxyl (**23**)

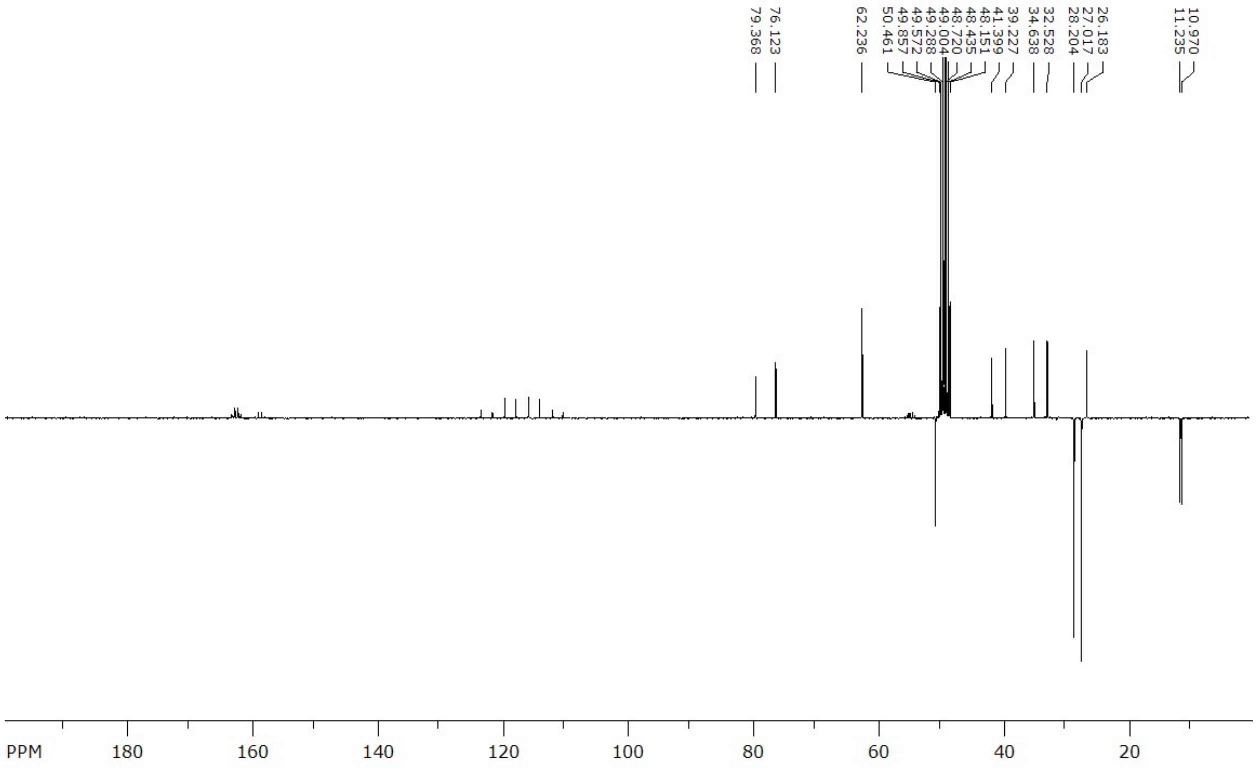

Figure S33. ^{1}H NMR (400 MHz; CD_3OD, Zn/CF_3COOH system) of ((2S(R),3R(S),5S(R))-2,5-di-*tert*-butyl-2,5-diethyl-3-((((3-(trimethylammonio)propyl)carbamoyl)oxy)methyl)pyrrolidin-1-oxyl) monoiodide (**26**)

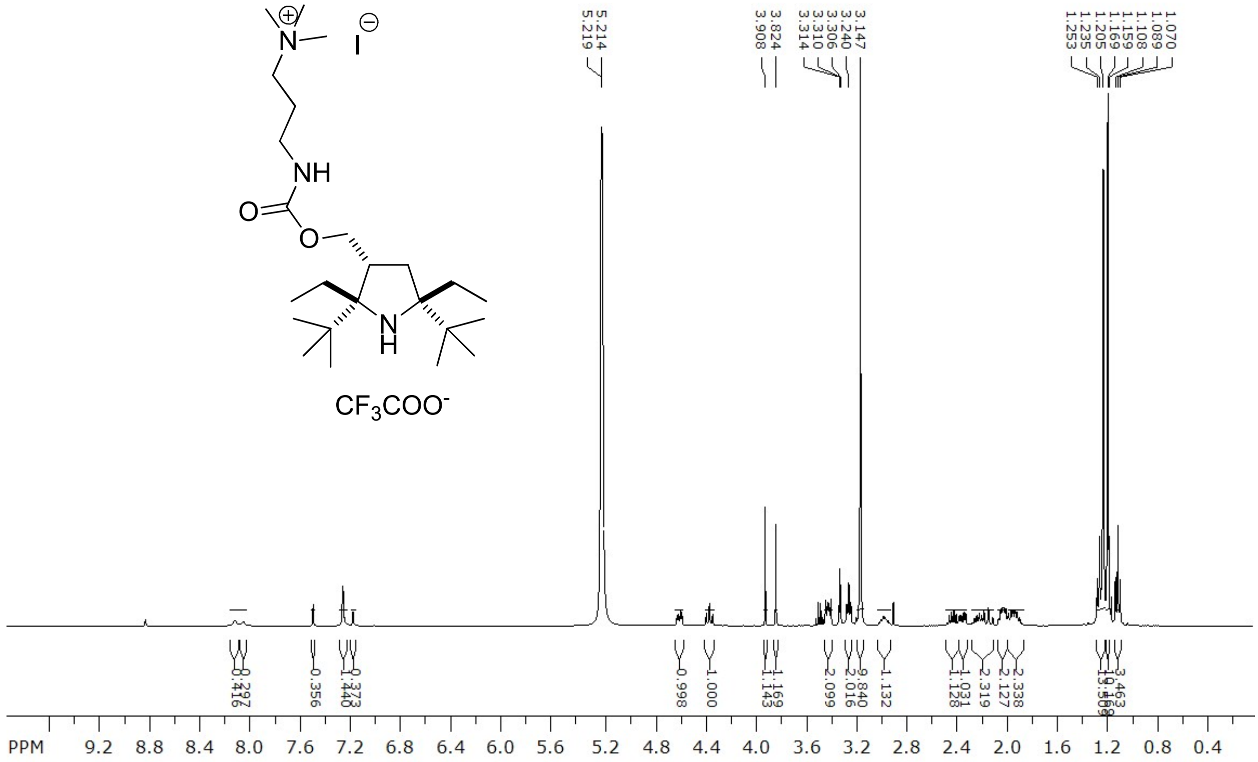

Figure S34. ^{13}C NMR (100 MHz; CD_3OD, Zn/CF_3COOH system) of ((2S(R),3R(S),5S(R))-2,5-di-*tert*-butyl-2,5-diethyl-3-((((3-(trimethylammonio)propyl)carbamoyl)oxy)methyl)pyrrolidin-1-oxyl) monoiodide (**26**)

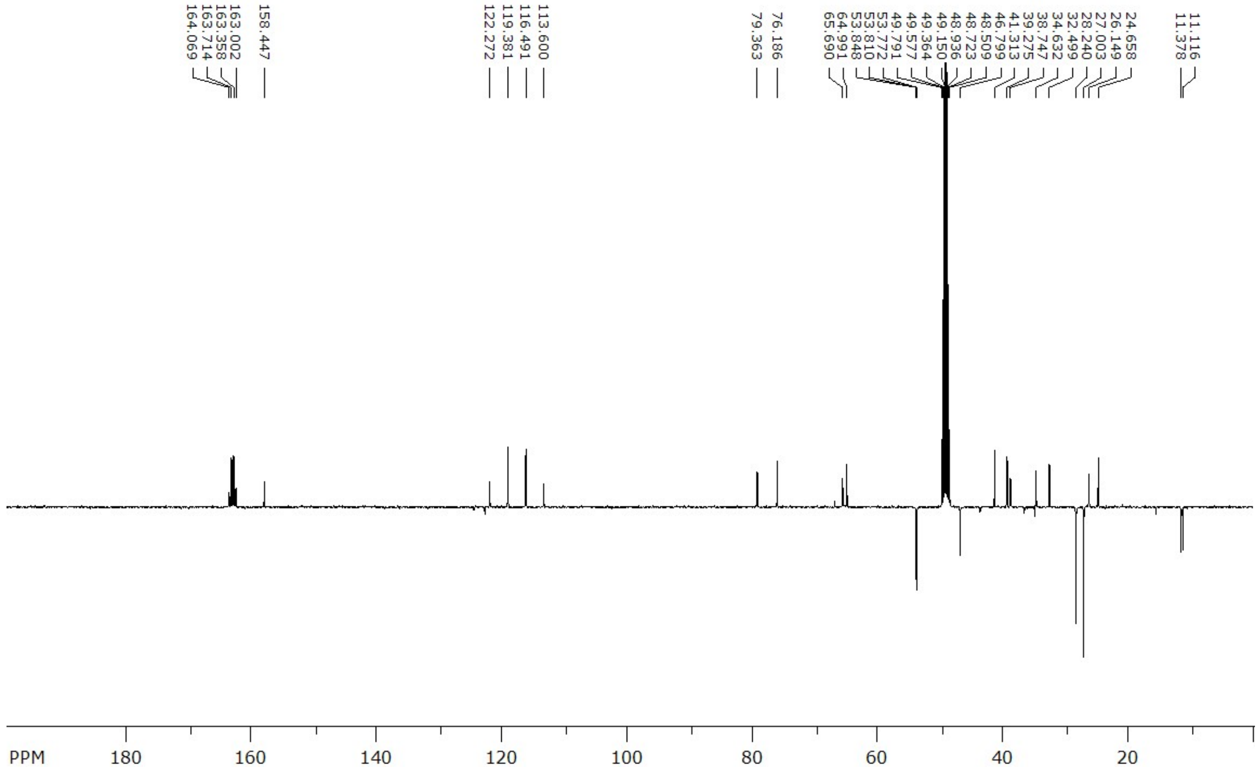

2D NMR Spectra

Figure S35. HMBC (400 MHz; $(CD_3)_2CO$; CF_3COOH) of (2R(S),3R(S),4R(S),5S(R))-2,5-di-*tert*-butyl-3,4-bis(methoxycarbonyl)-2-ethylpyrrolidine (**8**)

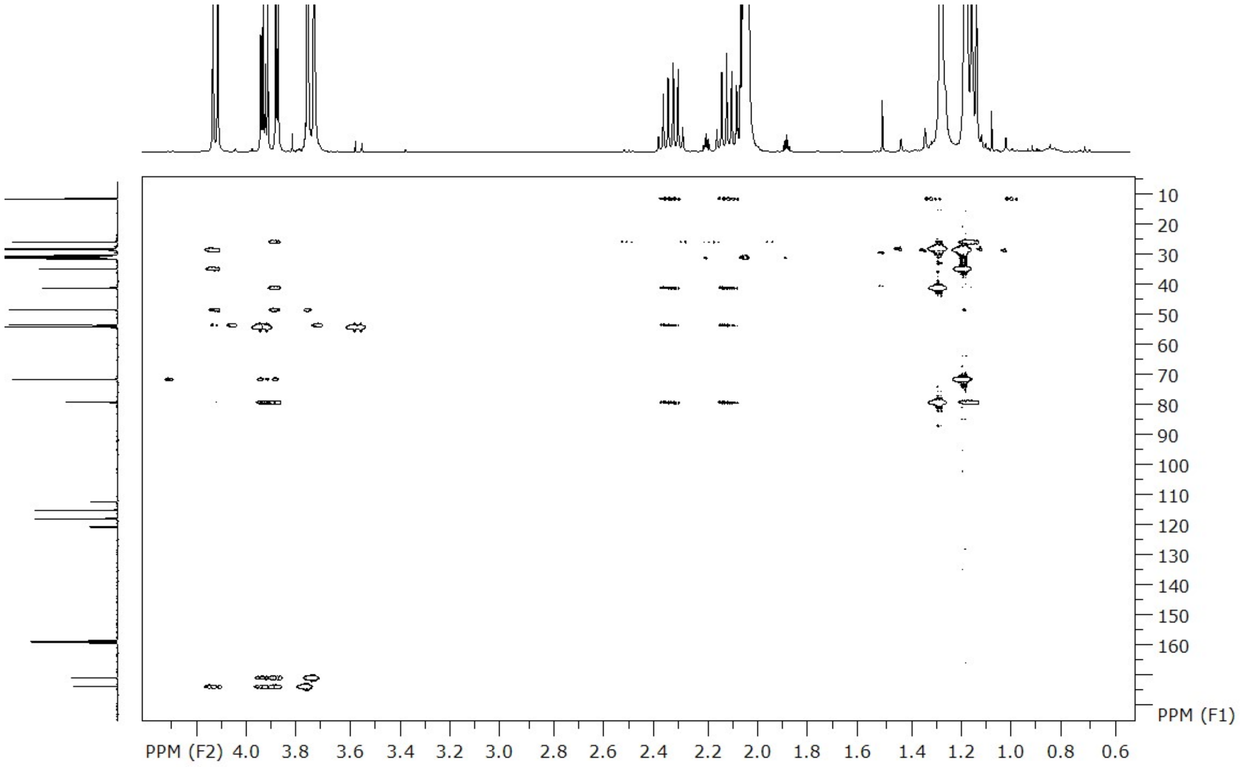

Figure S36. NOESY (400 MHz; $(CD_3)_2CO$; CF_3COOH) of (2R(S),3R(S),4R(S),5S(R))-2,5-di-*tert*-butyl-3,4-bis(methoxycarbonyl)-2-ethylpyrrolidine (**8**)

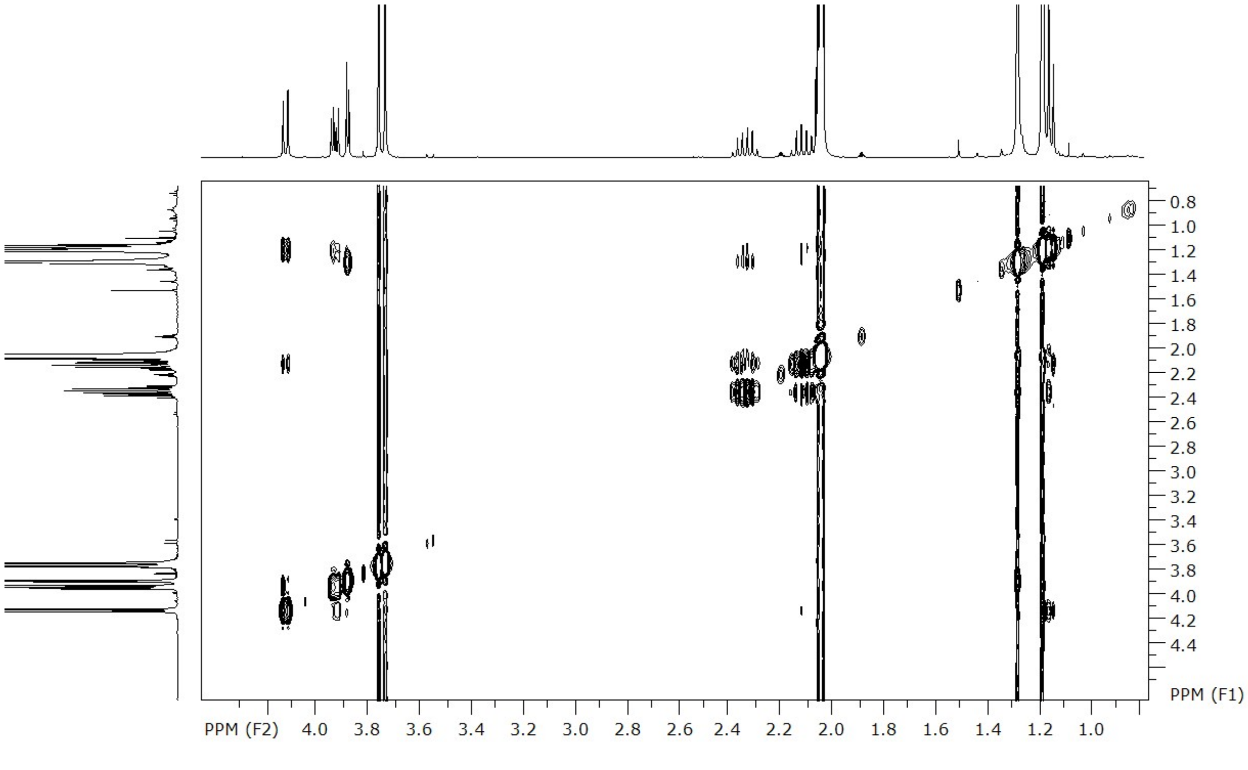

Figure S37. NOESY (600 MHz; $CDCl_3$) of (2S(R),3R(S),4R(S),5R(S))-2,5-di-*tert*-butyl-3,4-bis(methoxycarbonyl)-2-ethylpyrrolidine (**9**)

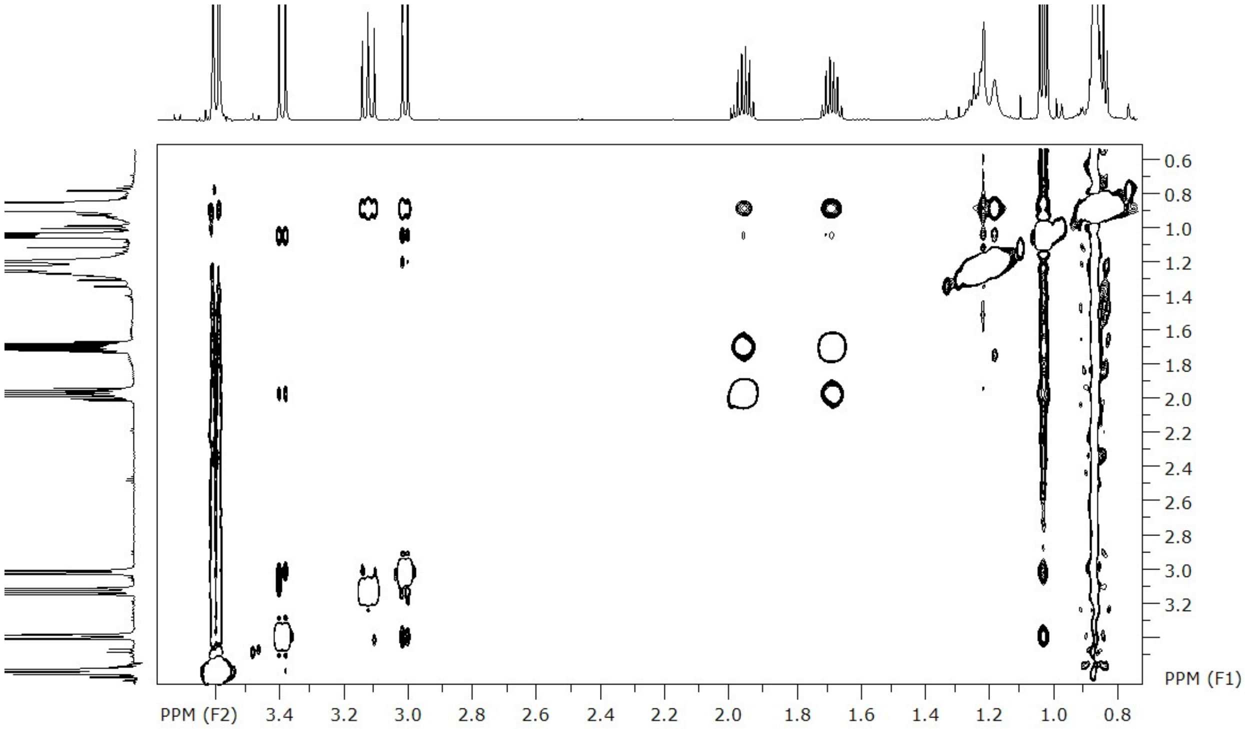

Figure S38. HMBC (400 MHz; $CDCl_3$) of (2R(S),3R(S),4R(S))-2,5-di-*tert*-butyl-2-ethyl-3,4-bis(hydroxymethyl)-3,4-dihydro-2*H*-pyrrole 1-oxide (**12**)

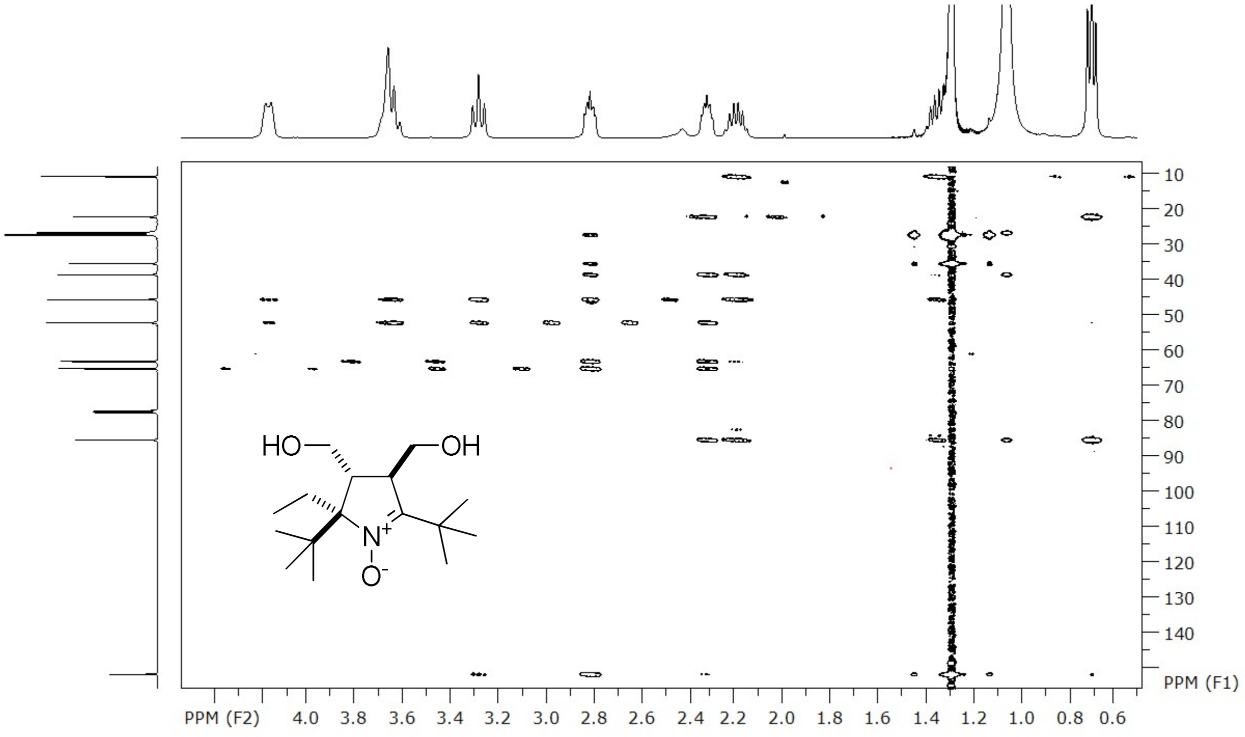

Figure S39. NOESY (400 MHz; $CDCl_3$) of (2R(S),3R(S),4R(S))-2,5-di-*tert*-butyl-2-ethyl-3,4-bis(hydroxymethyl)-3,4-dihydro-2*H*-pyrrole 1-oxide (**12**)

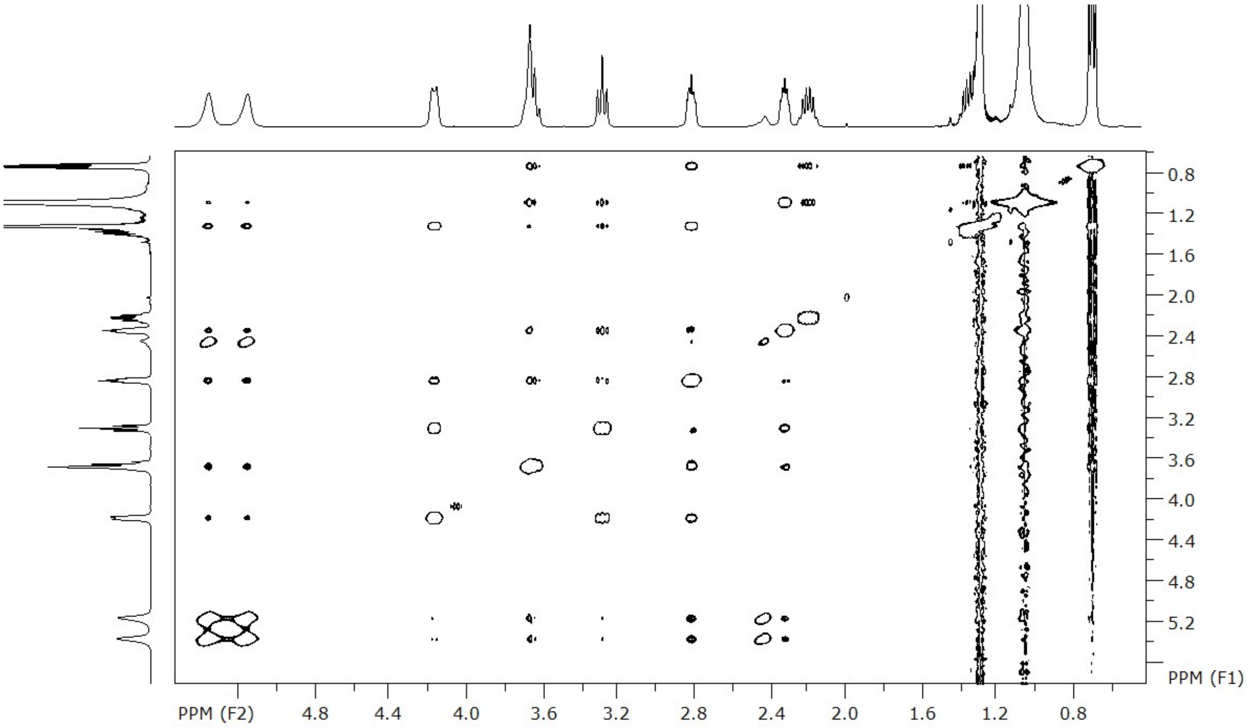

Figure S40. COSY (400 MHz, $CDCl_3$) of (1S(R),4R(S),5R(S))-2,4-di-*tert*-butyl-4-ethyl-1-propyl-3-azabicyclo[3.1.0]hex-2-ene (**15**)

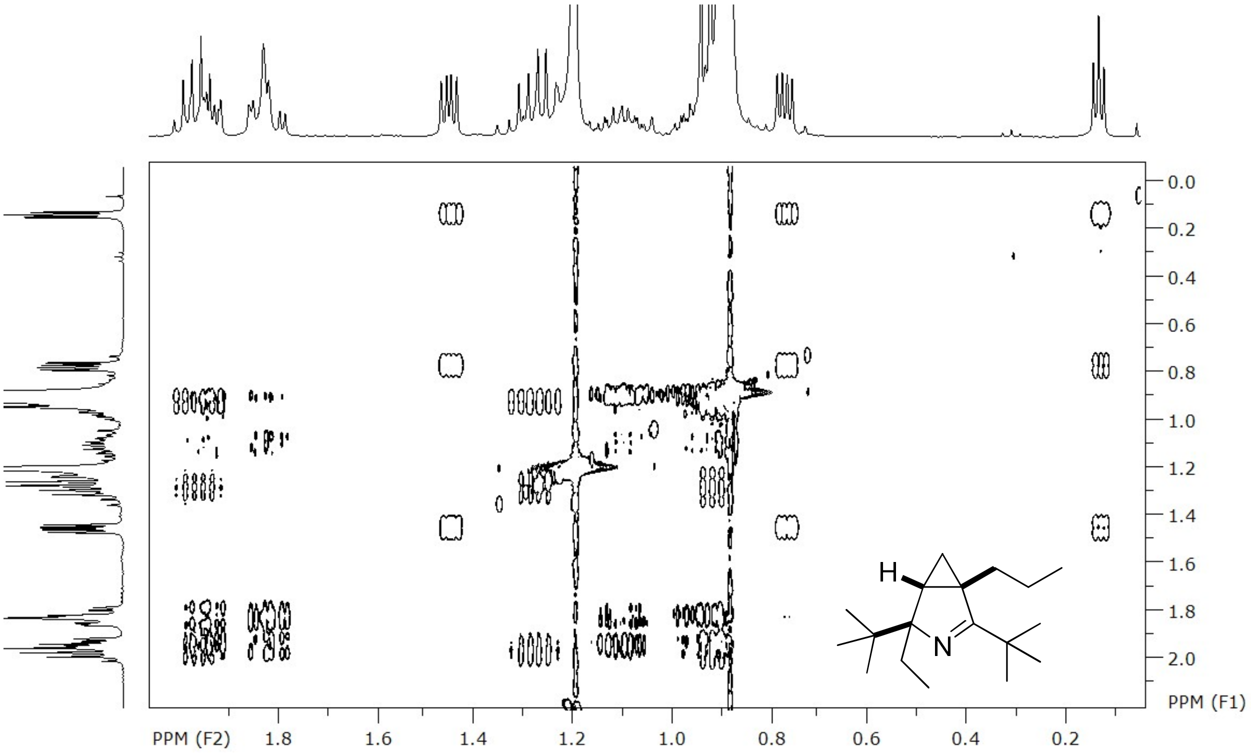

Figure S41. HSQC (400 MHz, $CDCl_3$) of (1S(R),4R(S),5R(S))-2,4-di-*tert*-butyl-4-ethyl-1-propyl-3-azabicyclo[3.1.0]hex-2-ene (**15**)

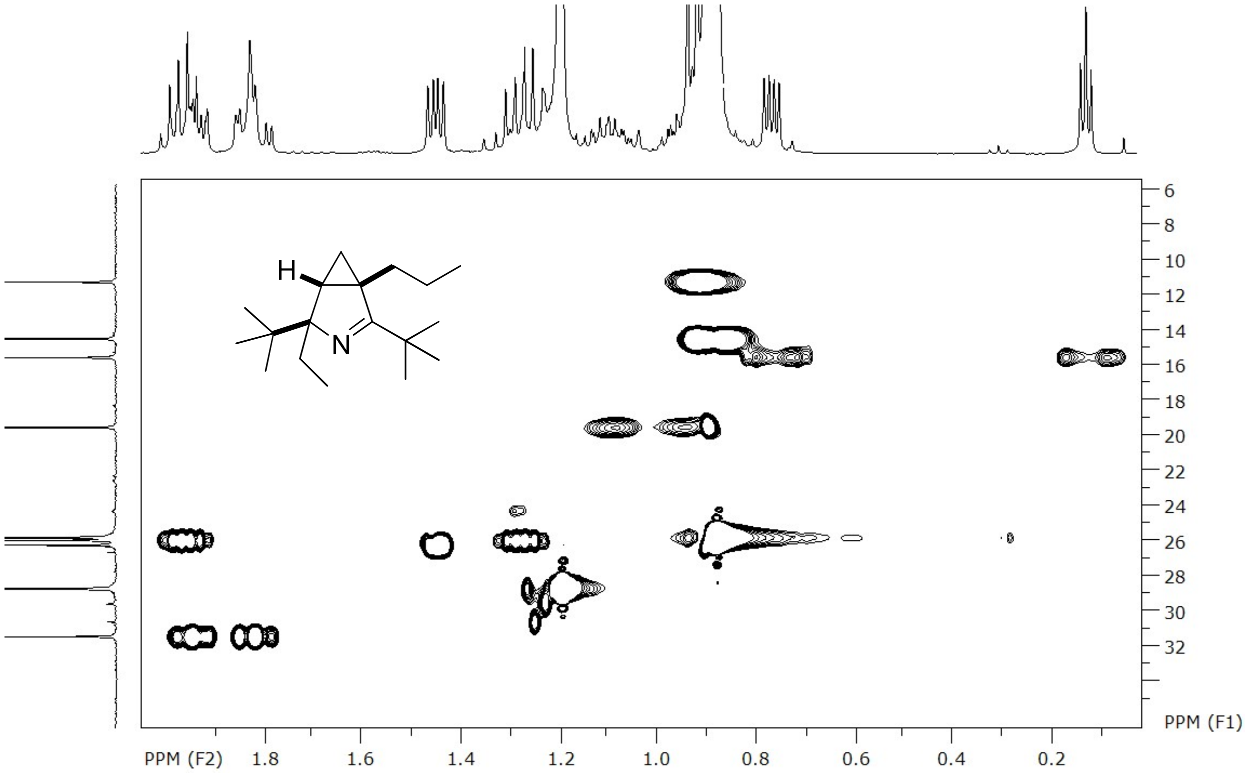

Figure S42. HMBC (400 MHz, $CDCl_3$) of (1S(R),4R(S),5R(S))-2,4-di-*tert*-butyl-4-ethyl-1-propyl-3-azabicyclo[3.1.0]hex-2-ene (**15**)

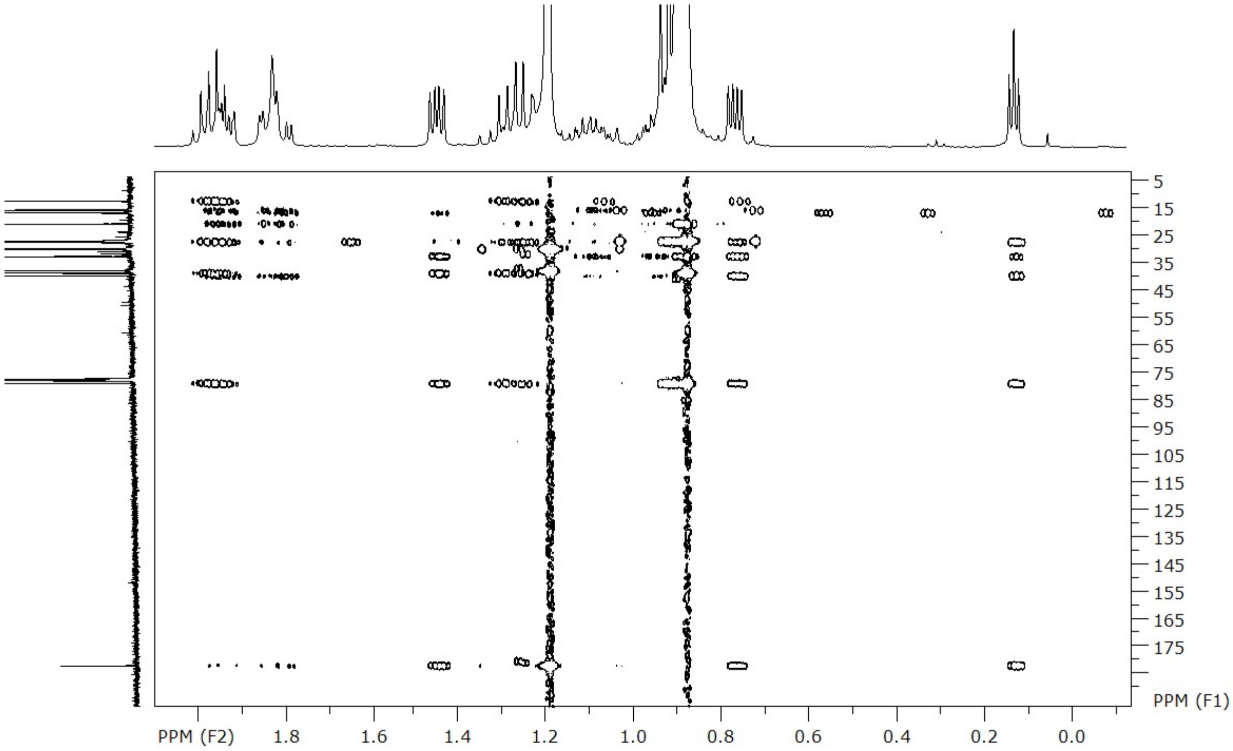

Figure S43. NOESY (400 MHz, $CDCl_3$) of (1S(R),4R(S),5R(S))-2,4-di-*tert*-butyl-4-ethyl-1-propyl-3-azabicyclo[3.1.0]hex-2-ene (**15**)

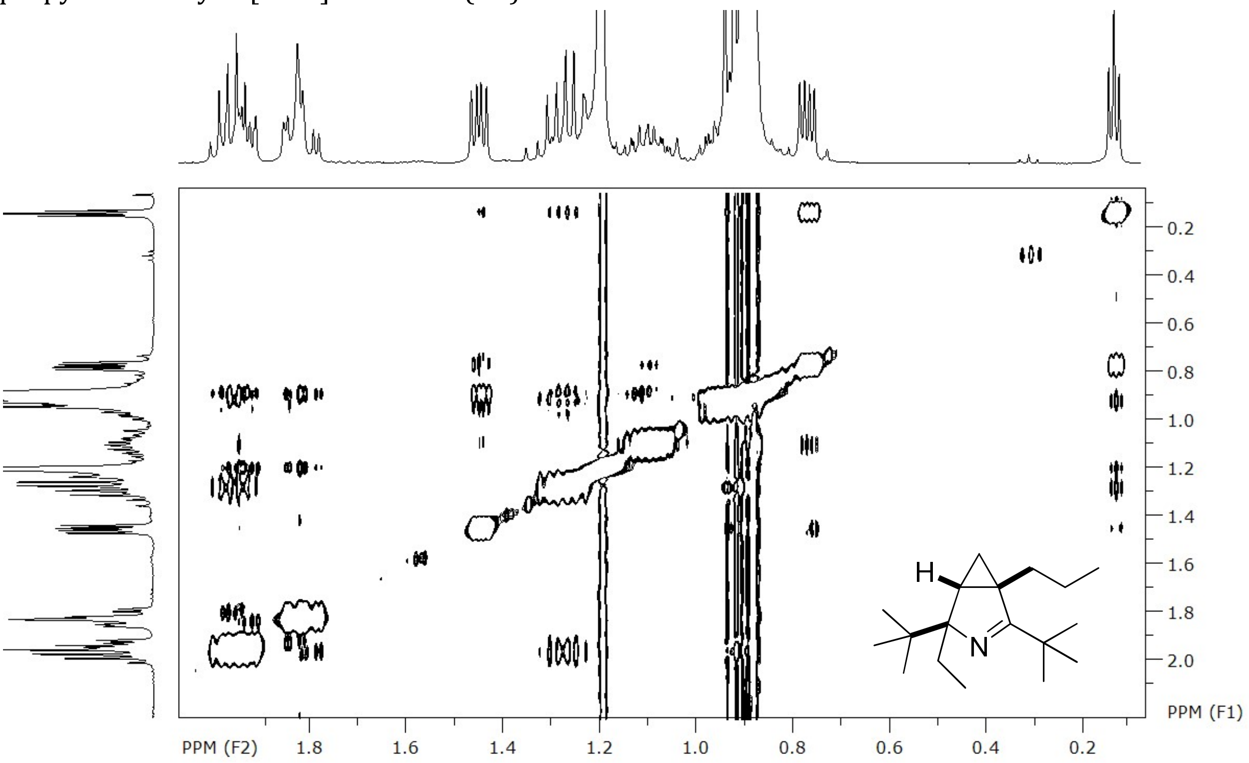

Figure S44. COSY (400 MHz; CD_3OD, Zn/CF_3COOH system) of ((2S(R),3R(S),5S(R))-2,5-di-*tert*-butyl-2,5-diethyl-3-((((3-(trimethylammonio)propyl)carbamoyl)oxy)methyl)pyrrolidin-1-oxyl) monoiodide (**26**)

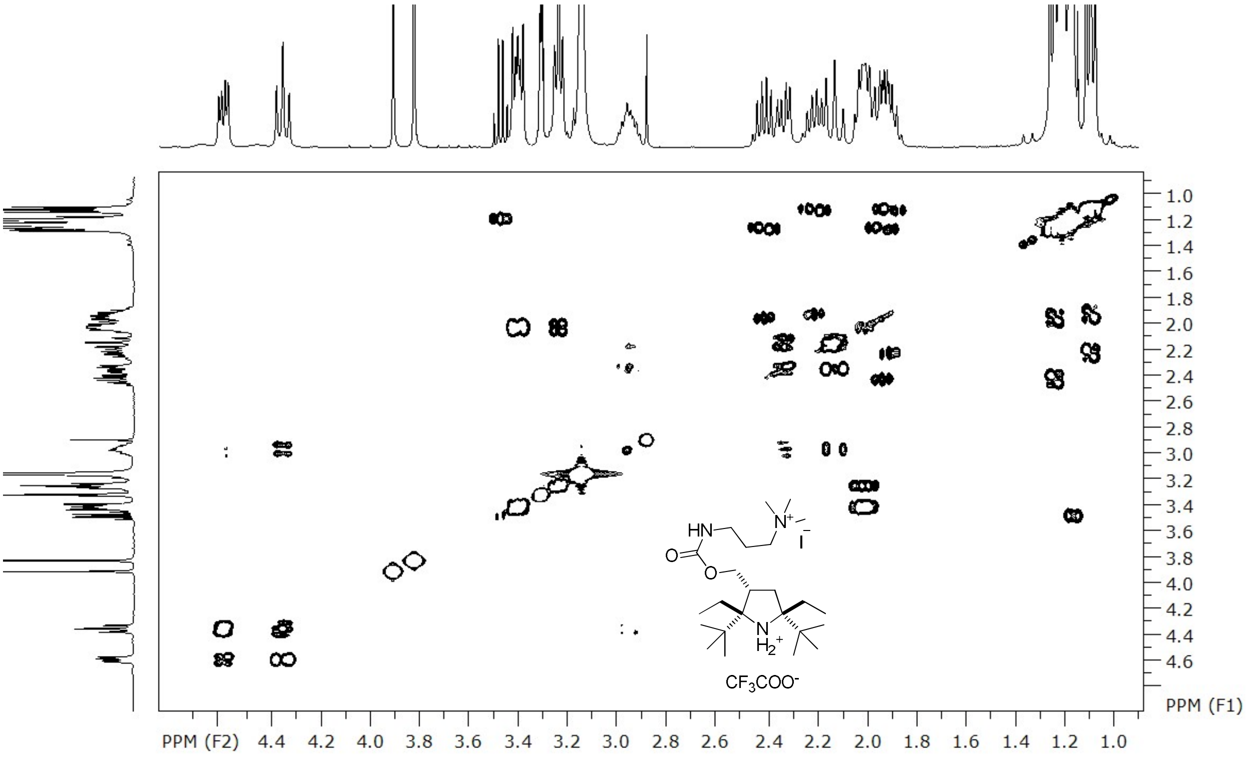

Figure S45. HSQC (400 MHz; CD_3OD, Zn/CF_3COOH system) of ((2S(R),3R(S),5S(R))-2,5-di-*tert*-butyl-2,5-diethyl-3-((((3-(trimethylammonio)propyl)carbamoyl)oxy)methyl)pyrrolidin-1-oxyl) monoiodide (**26**)

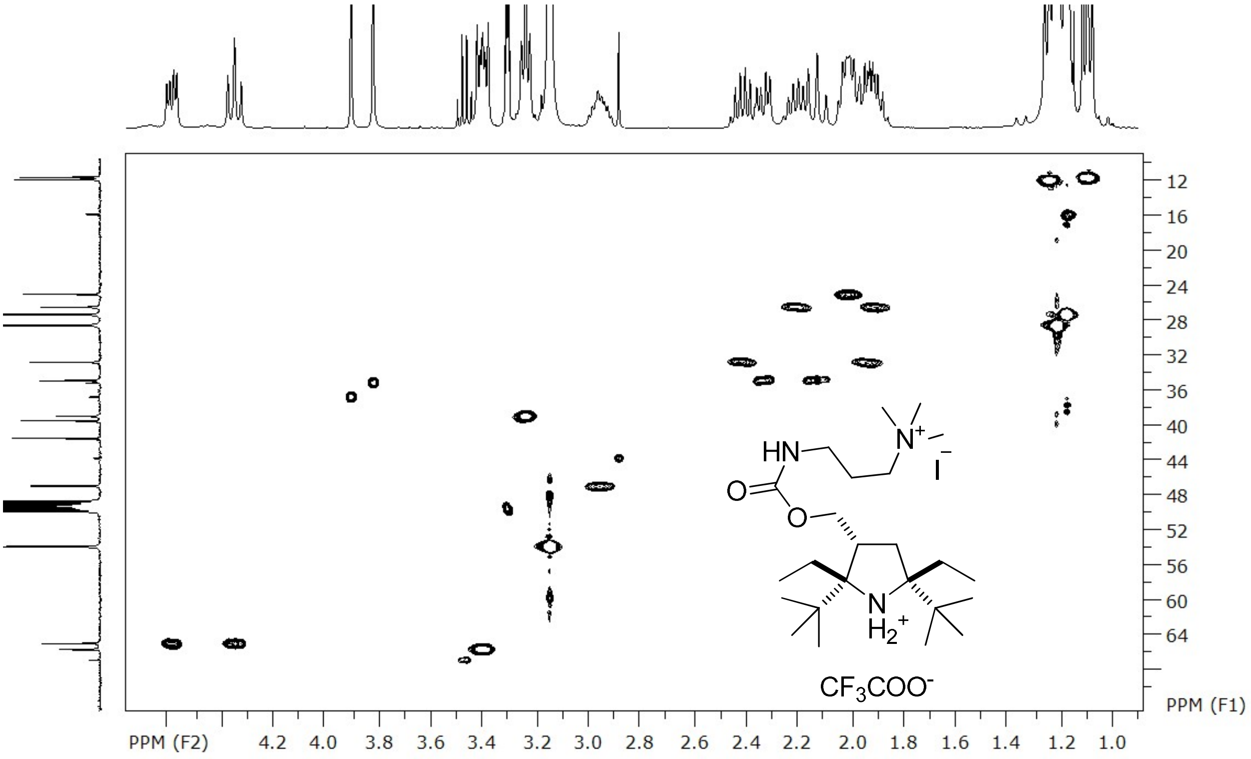

Figure S46. HMBC (400 MHz; CD_3OD, Zn/CF_3COOH system) of ((2S(R),3R(S),5S(R))-2,5-di-*tert*-butyl-2,5-diethyl-3-((((3-(trimethylammonio)propyl)carbamoyl)oxy)methyl)pyrrolidin-1-oxyl) monoiodide (**26**)

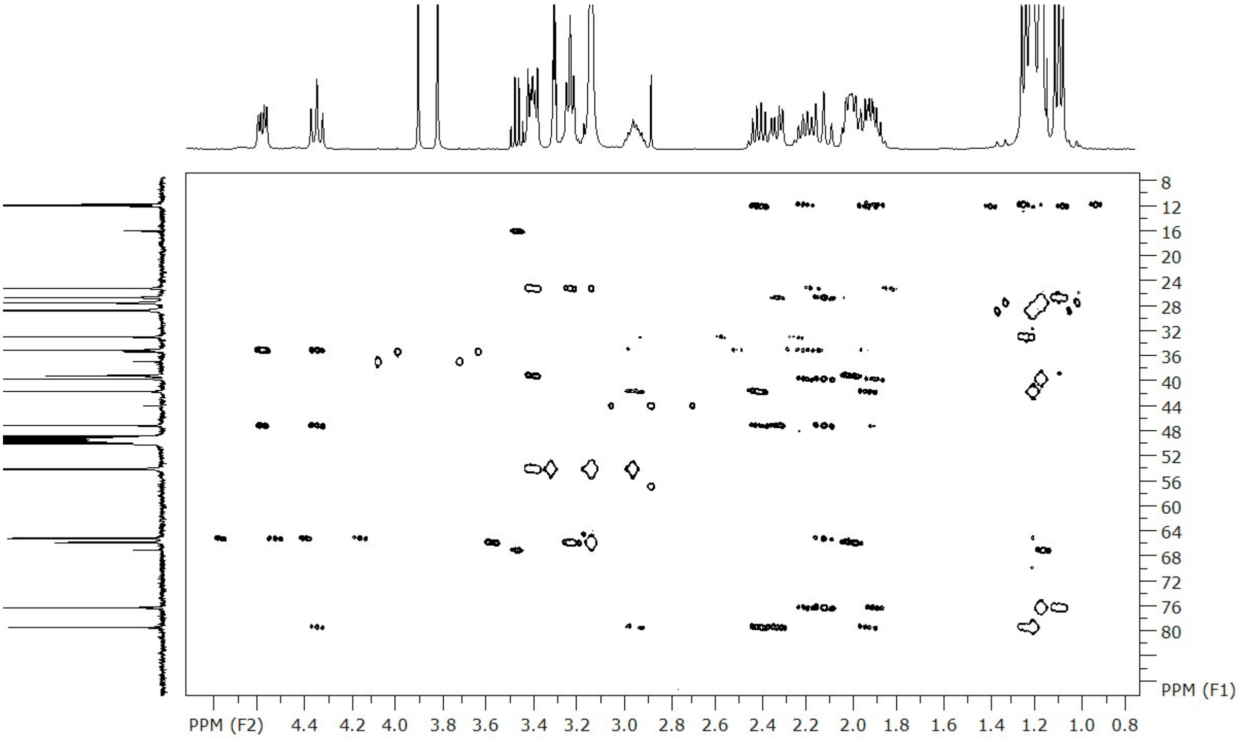

Figure S47. NOESY (400 MHz; CD_3OD, Zn/CF_3COOH system) of ((2S(R),3R(S),5S(R))-2,5-di-*tert*-butyl-2,5-diethyl-3-((((3-(trimethylammonio)propyl)carbamoyl)oxy)methyl)pyrrolidin-1-oxyl) monoiodide (**26**)

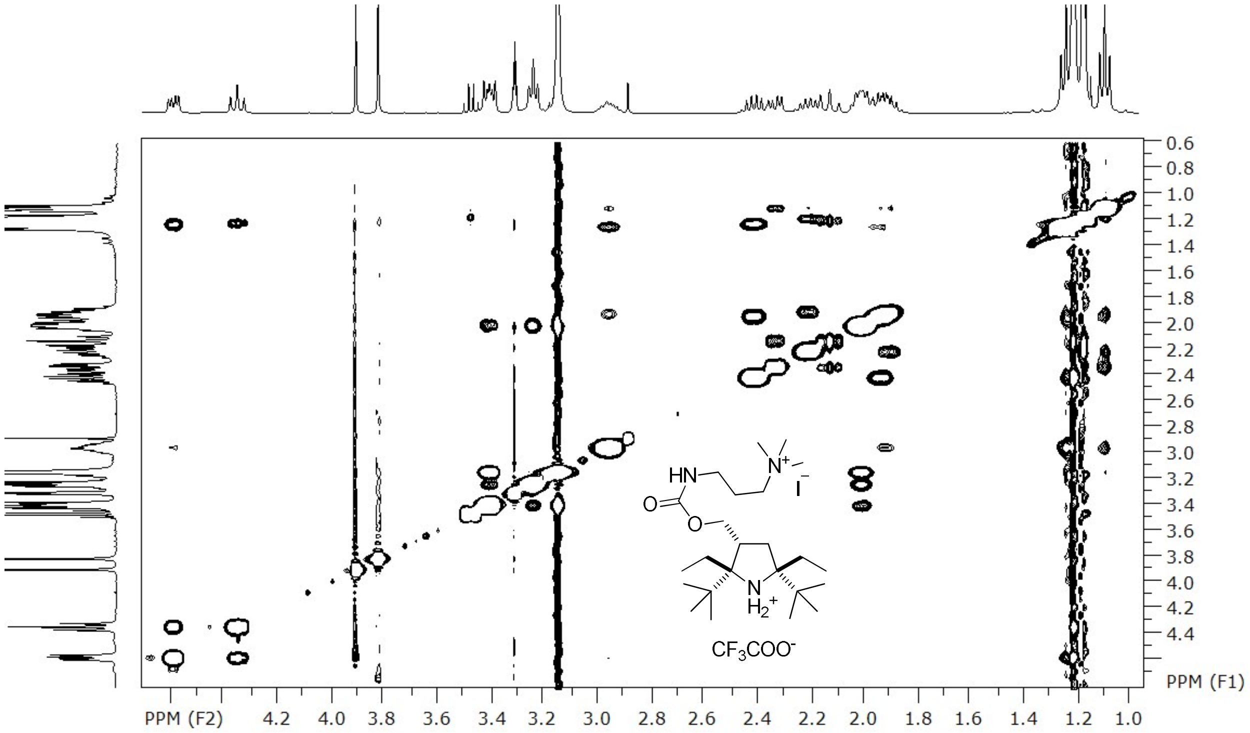

Line shape analysis

The analysis was performed using the gNMR 5.0 software [Budzelaar, P. H. M. "gNMR, version 5.0. 6.0." *Ivorysoft, Nijmegen, Netherlands* (2006).].

Figure S48. Simulated spectrum for (1S(R),4R(S),5R(S))-2,4-di-tert-butyl-4-ethyl-1-propyl-3-azabicyclo[3.1.0]hex-2-ene (**15**)

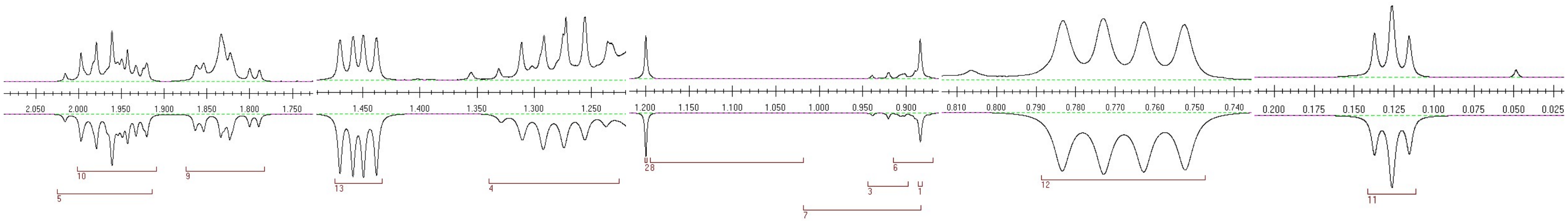

Table S1. Parameters of spins system (1S(R),4R(S),5R(S))-2,4-di-tert-butyl-4-ethyl-1-propyl-3-azabicyclo[3.1.0]hex-2-ene (**15**)

#	n	Shift	Width	J[1]	J[2]	J[3]	J[4]	J[5]	J[6]	J[7]	J[8]	J[9]	J[10]	J[11]	J[12]
1	9	0.884	1.53												
2	9	1.200	1.41	-											
3	3	0.921	1.24	-	-										
4	1	1.283	2.94	-	-	7.85									
5	1	1.969	1.72	-	-	7.21	-14.54								
6	3	0.892	1.25	-	-	-	-	-	7.4*						
7	1	0.951*	-	-	-	-	-	-	7.4*	-14.4*					
8	1	1.106*	-	-	-	-	-	-	-	4.31	12.31				
9	1	1.829	1.77	-	-	-	-	-	-	5.18	11.42	-13.57			
10	1	1.955	1.82	-	-	-	-	-	-	-	-	-	-		
11	1	0.126	1.78	-	-	-	-	-	-	-	-	-	-		
12	1	0.768	1.97	-	-	-	-	-	-	-	-	-	-	-4.20	
13	1	1.454	1.70	-	-	-	-	-	-	-	-	-	-	4.56	8.24

*****the data is not reliably determined

Figure S49. Simulated spectrum for mono((2S(R),3R(S),5S(R))-2,5-di-*tert*-butyl-2,5-diethyl-3-((((3-(trimethylammonio)propyl)carbamoyl)oxy)methyl)pyrrolidin-1-oxyl) monoiodide (**26**)

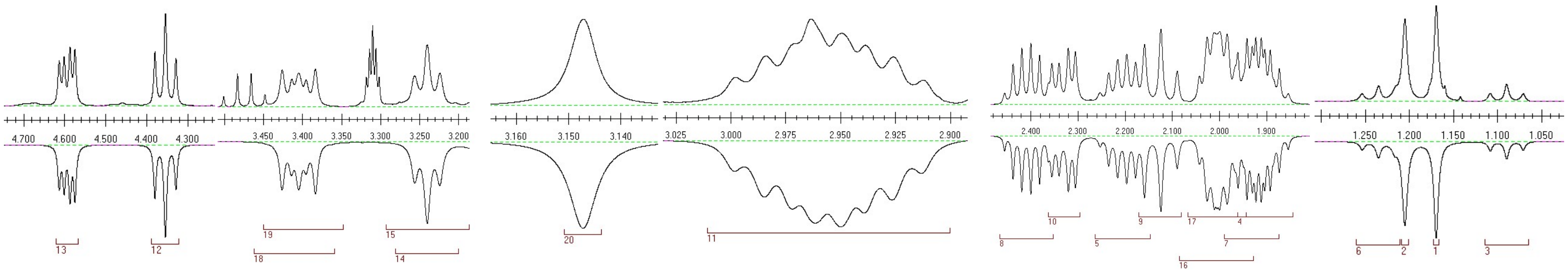

Table S2. Parameters of spins system for mono((2S(R),3R(S),5S(R))-2,5-di-*tert*-butyl-2,5-diethyl-3-((((3-(trimethylammonio)propyl)carbamoyl)oxy)methyl)pyrrolidin-1-oxyl) monoiodide (**26**)

#	n	Shift	Width	J[1]	J[2]	J[3]	J[4]	J[5]	J[6]	J[7]	J[8]	J[9]	J[10]	J[11]	J[12]	J[13]	J[14]	J[15]	J[16]	J[17]	J[18]	J[19]
1	9	1.169	2.45																			
2	9	1.205	2.84	-																		
3	3	1.090	2.25	-	-																	
4	1	1.903	2.89	-	-	7.59																
5	1	2.205	3.33	-	-	7.37	-15.41															
6	3	1.235	2.59	-	-	-	-	-														
7	1	1.933	2.75	-	-	-	-	-	7.43													
8	1	2.409	2.44	-	-	-	-	-	7.25	-15.23												
9	1	2.126	3.73	-	-	-	-	-	-	-	-											
10	1	2.329	3.35	-	-	-	-	-	-	-	-	-13.94										
11	1	2.955	3.92	-	-	-	-	-	-	-	-	13.72	5.99									
12	1	4.355	3.10	-	-	-	-	-	-	-	-	-	-	9.98								
13	1	4.594	3.19	-	-	-	-	-	-	-	-	-	-	4.85	-10.59							
14	1	3.241	4.20	-	-	-	-	-	-	-	-	-	-	-	-	-						
15	1	3.240	2.88	-	-	-	-	-	-	-	-	-	-	-	-	-	-16.26*					
16	1	2.006	2.63	-	-	-	-	-	-	-	-	-	-	-	-	-	4.75	9.92				
17	1	2.005	3.40	-	-	-	-	-	-	-	-	-	-	-	-	-	2.98	8.09	-16.53*			
18	1	3.410	2.85	-	-	-	-	-	-	-	-	-	-	-	-	-	-	-	6.83	10.43		
19	1	3.399	2.62	-	-	-	-	-	-	-	-	-	-	-	-	-	-	-	14.69	2.46	-15.78*	
20	9	3.147	2.47	-	-	-	-	-	-	-	-	-	-	-	-	-	-	-	-	-	-	-

*the data is not reliably determined

IR Spectra

Figure S50. IR (neat) of (2R(S),3R(S),4R(S),5S(R))-2,5-di-*tert*-butyl-3,4-bis(methoxycarbonyl)-2-ethylpyrrolidine (**8**)

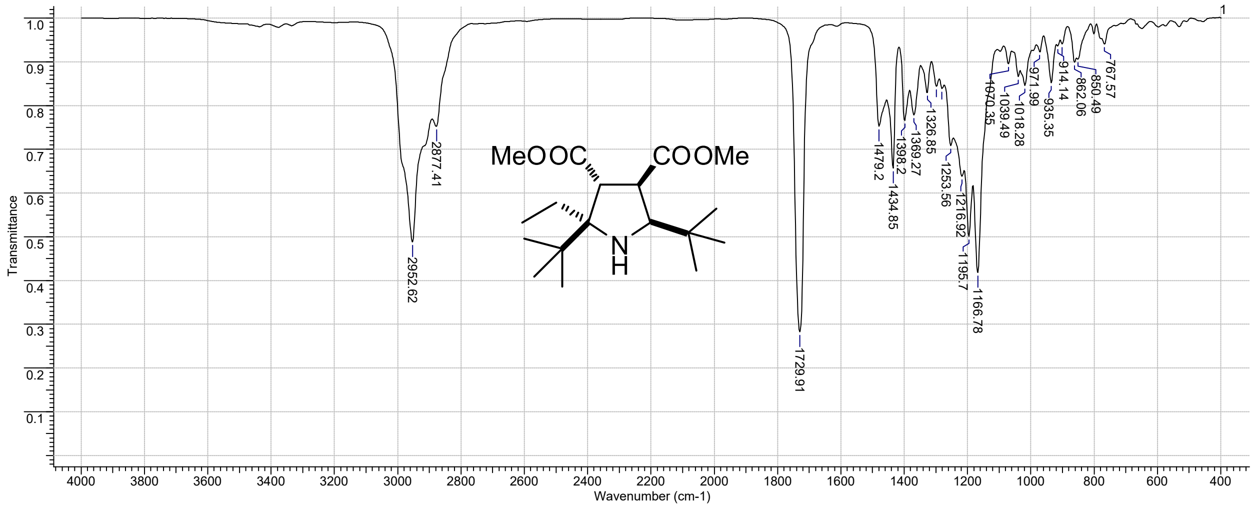

Figure S51. IR (neat) of (2S(R),3R(S),4R(S),5R(S))-2,5-di-*tert*-butyl-3,4-bis(methoxycarbonyl)-2-ethylpyrrolidine (**9**)

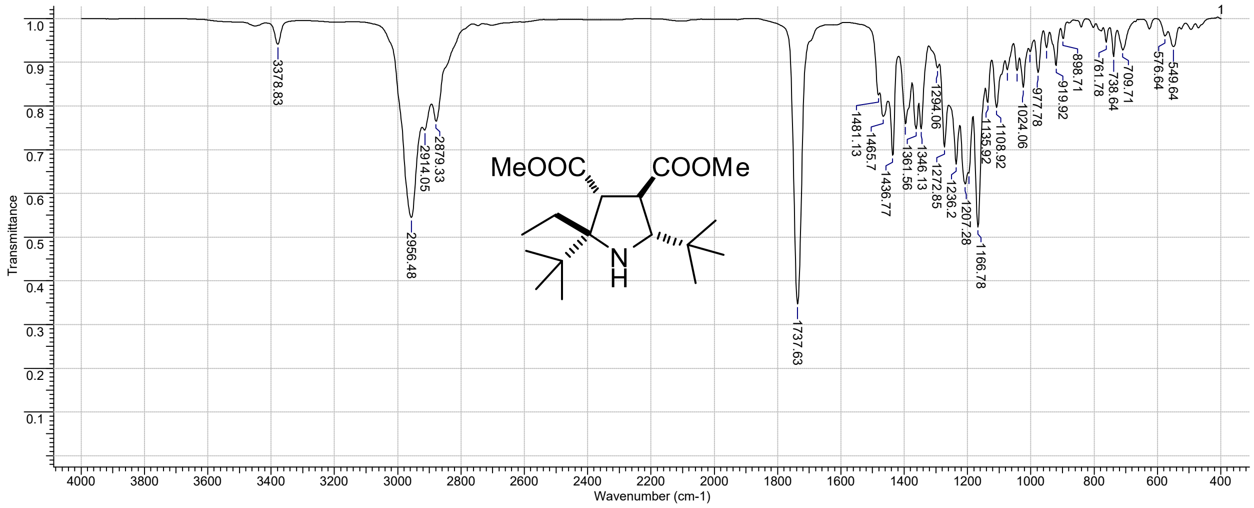

Figure S52. IR (neat) of dimethyl 2-(1-((2,2-dimethylpentan-3-ylidene)amino)-2,2-dimethylpropyl)succinate (**10**)

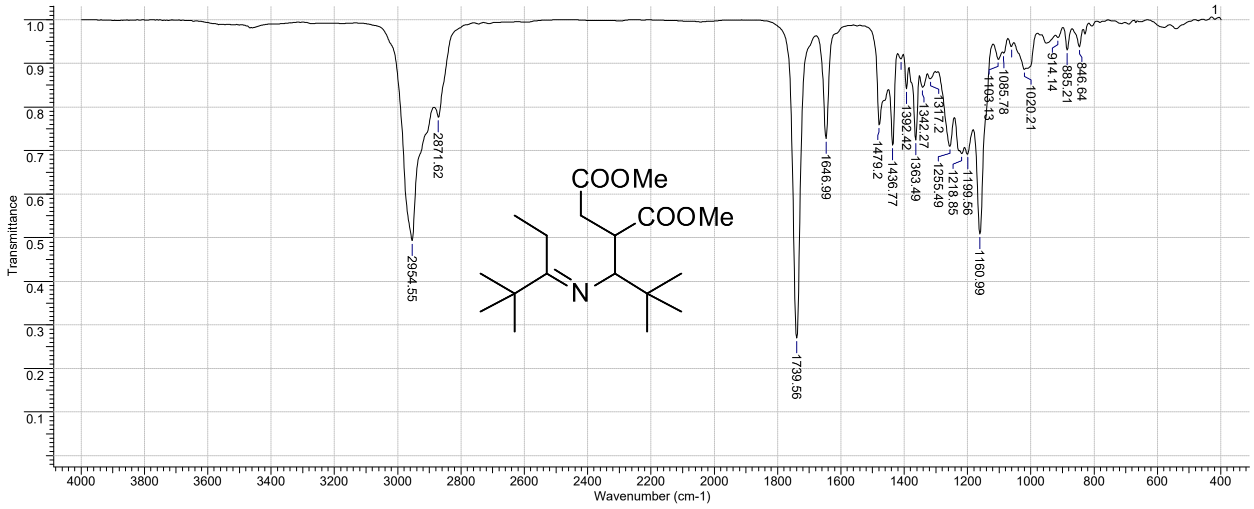

Figure S53. IR (KBr) of (2R(S),3R(S),4R(S),5S(R))-2,5-di-tert-butyl-3,4-bis(hydroxymethyl)-2-ethylpyrrolidine (**11**)

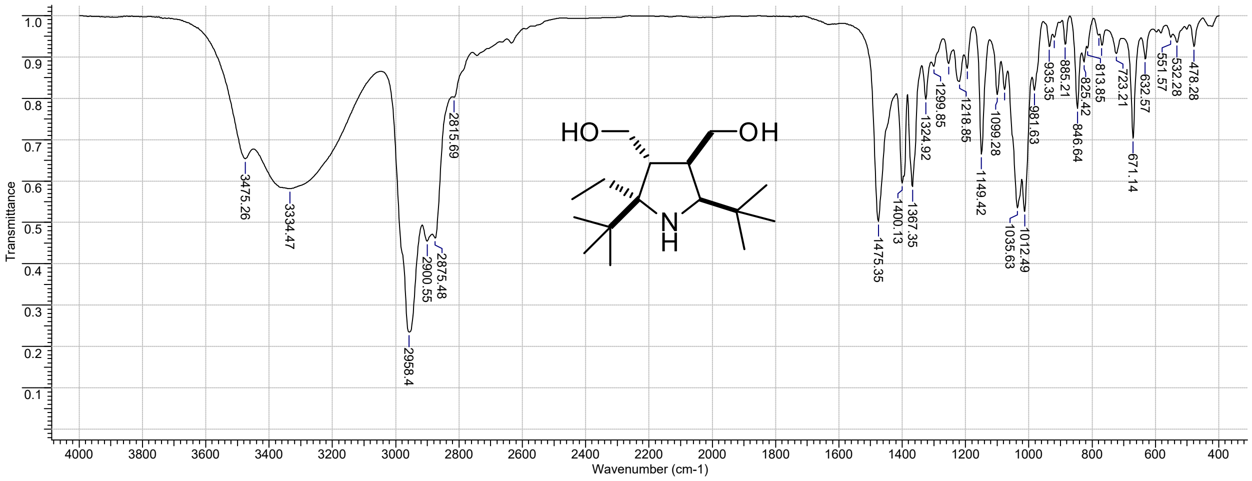

Figure S54. IR (KBr) of (2R(S),3R(S),4R(S))-2,5-di-tert-butyl-2-ethyl-3,4-bis(hydroxymethyl)-3,4-dihydro-2H-pyrrole 1-oxide (**12**)

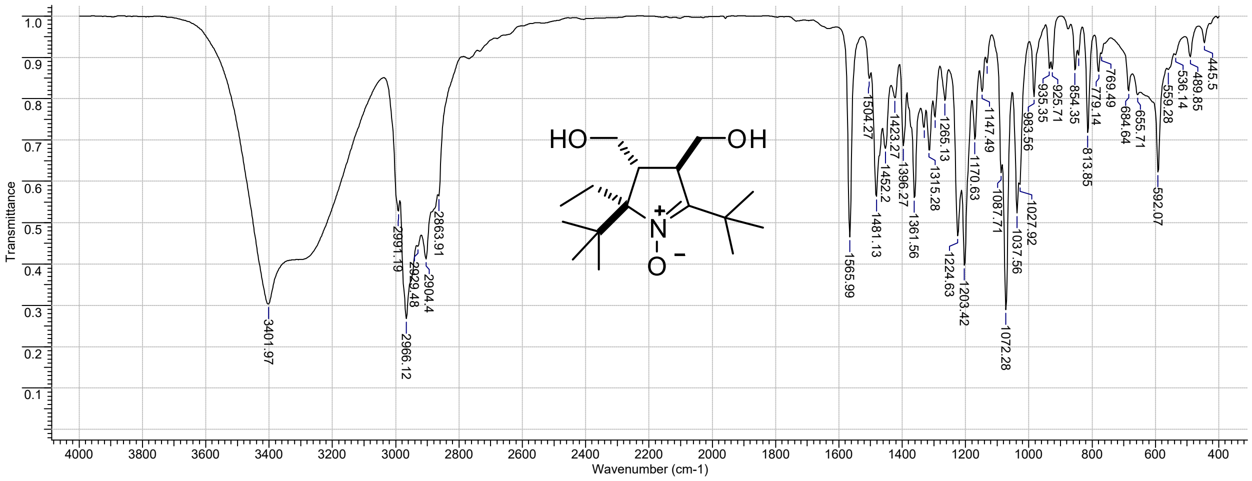

Figure S55. IR (KBr) of 2,2,5-Triethyl-5-tert-butyl-3,4-bis(hydroxymethyl)-pyrrolidine-1-oxyl (**1a**)

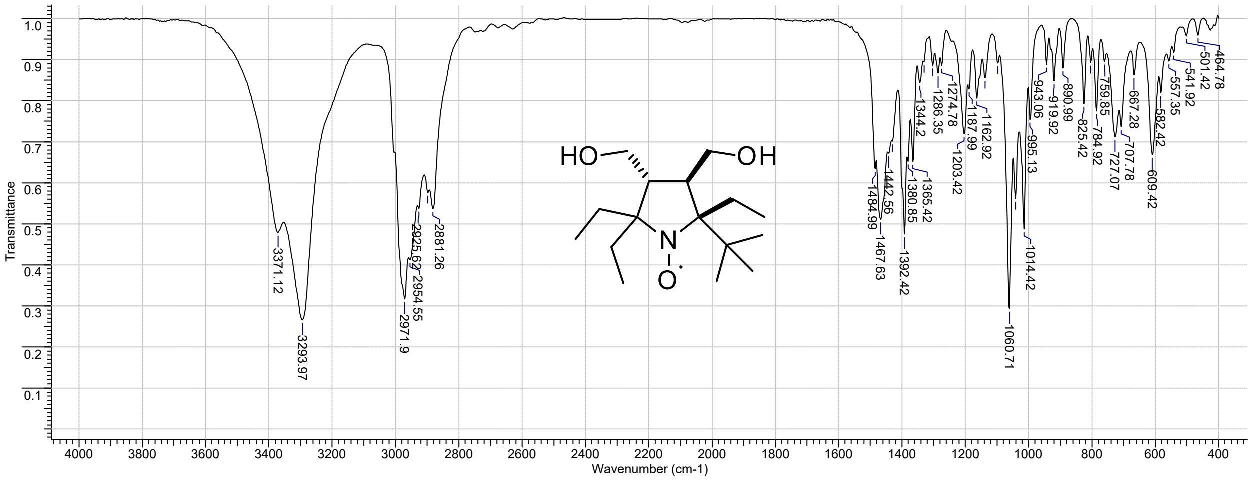

Figure S56. IR(KBr) of (2R(S),3R(S),4R(S))-2,5-di-tert-butyl-2-ethyl-3,4-bis(((2-methoxypropan-2-yl)oxy)methyl)-3,4-dihydro-2H-pyrrole 1-oxide (**14**)

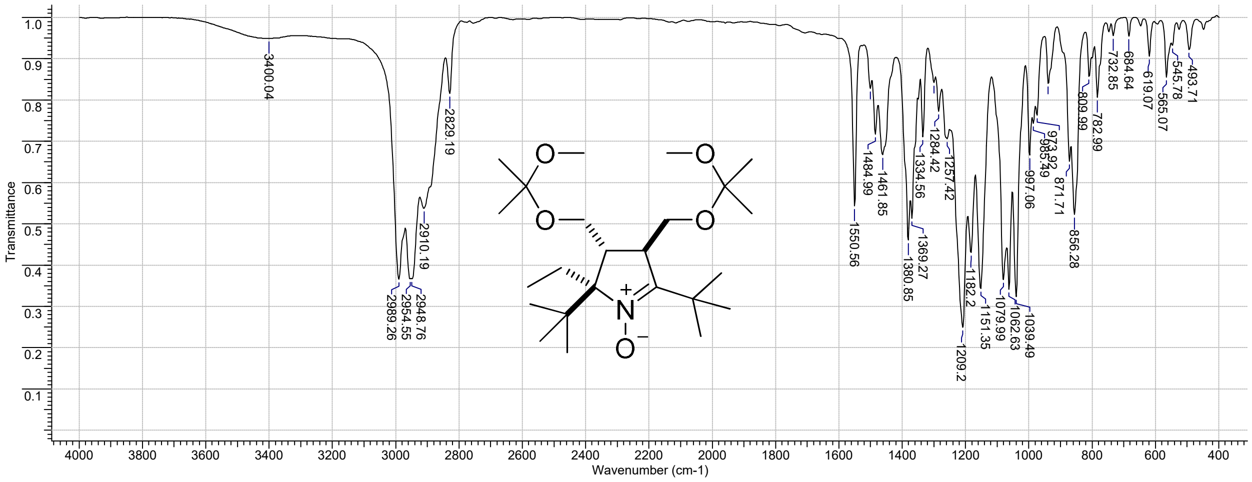

Figure S57. IR (neat) of (1S(R),4R(S),5R(S))-2,4-di-*tert*-butyl-4-ethyl-1-propyl-3-azabicyclo[3.1.0]hex-2-ene (**15**)

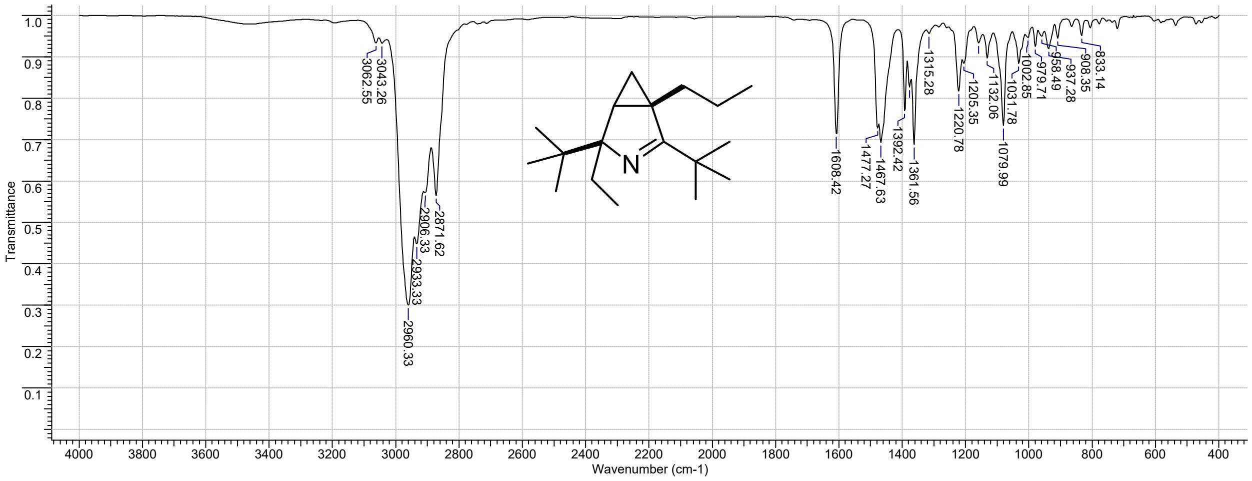

Figure S58. IR (neat) of (2S(R),3R(S),4R(S))-2,5-di-*tert*-butyl-2-ethyl-3,4-bis(methoxycarbonyl)-3,4-dihydro-2*H*-pyrrole 1-oxide (**16**)

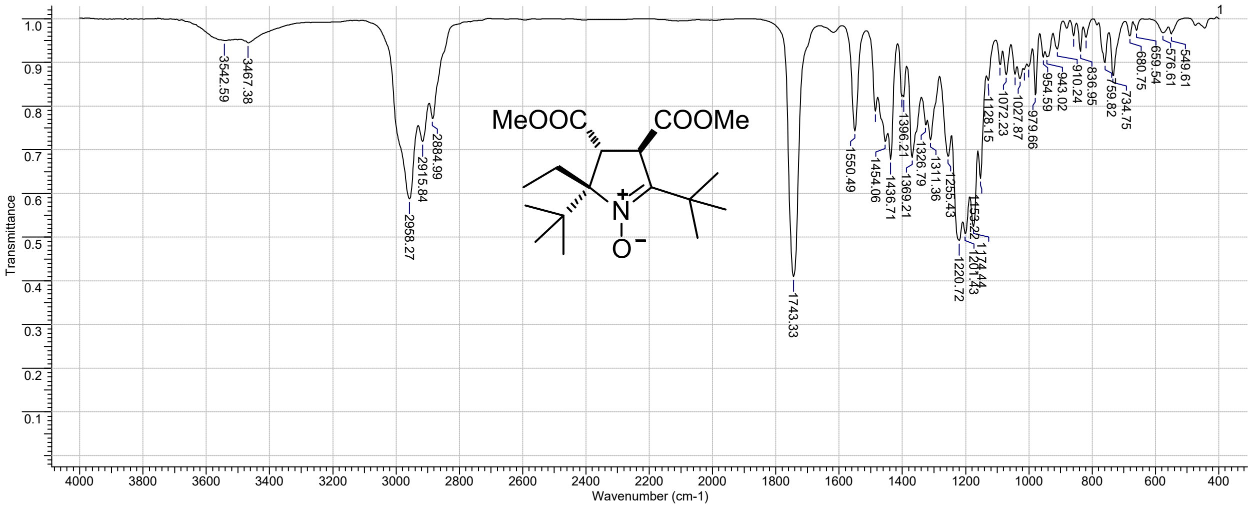

Figure S59. IR (KBr) of (2S(R),3R(S))-2,5-di-tert-butyl-2-ethyl-3-(methoxycarbonyl)-3,4-dihydro-2H-pyrrole 1-oxide (**17**)

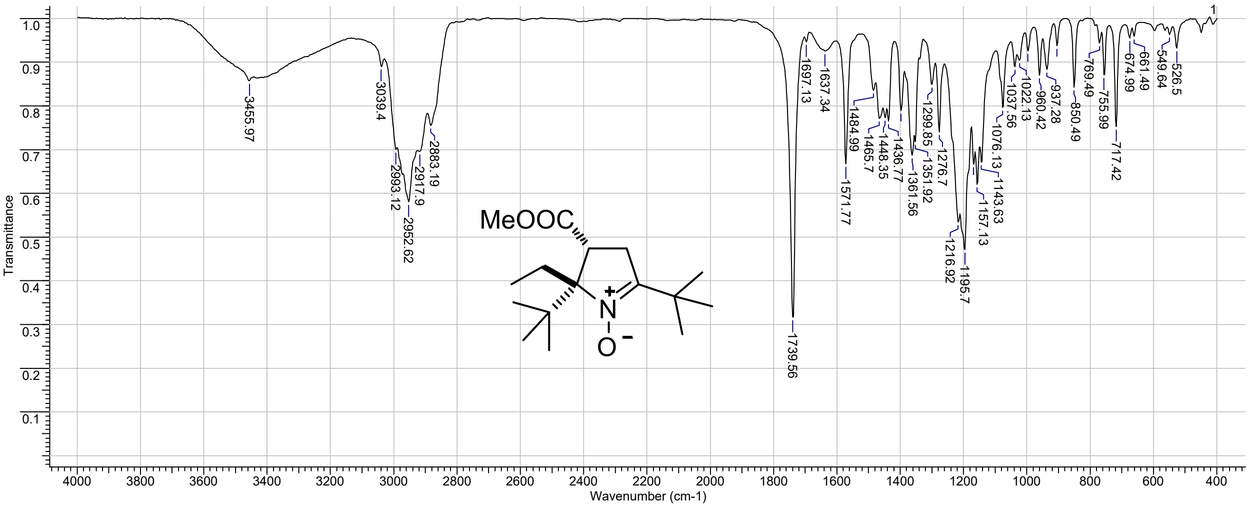

Figure S60. IR (KBr) of (2S(R),3S(R))-2,5-di-tert-butyl-2-ethyl-3-(methoxycarbonyl)-3,4-dihydro-2H-pyrrole 1-oxide (**18**)

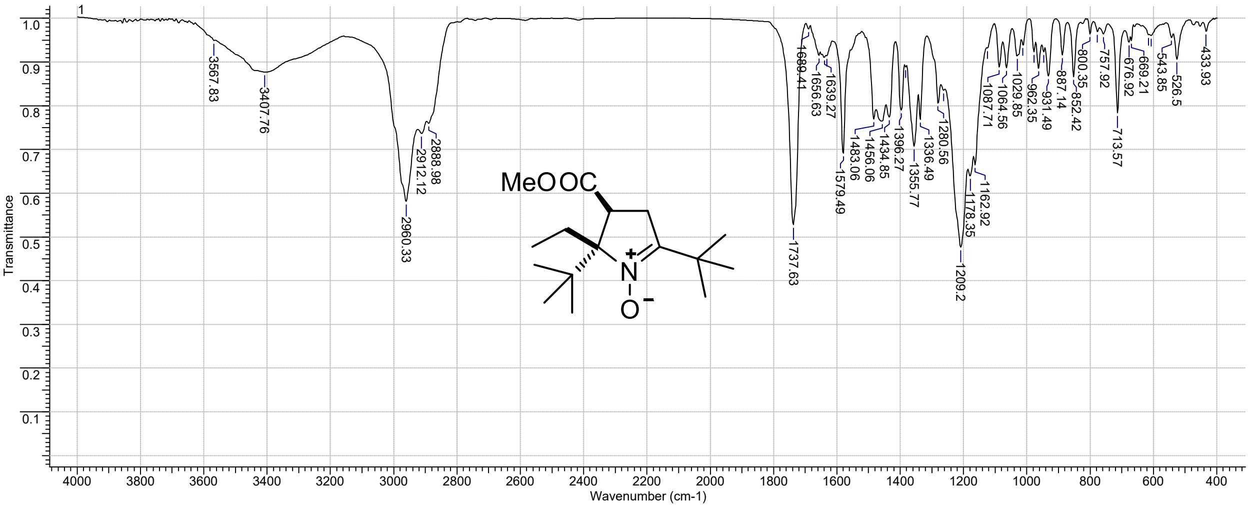

Figure S61. IR (KBr) of (2S(R),3R(S))-2,5-di-tert-butyl-2-ethyl-3-(hydroxymethyl)-3,4-dihydro-2H-pyrrole 1-oxide (**20**)

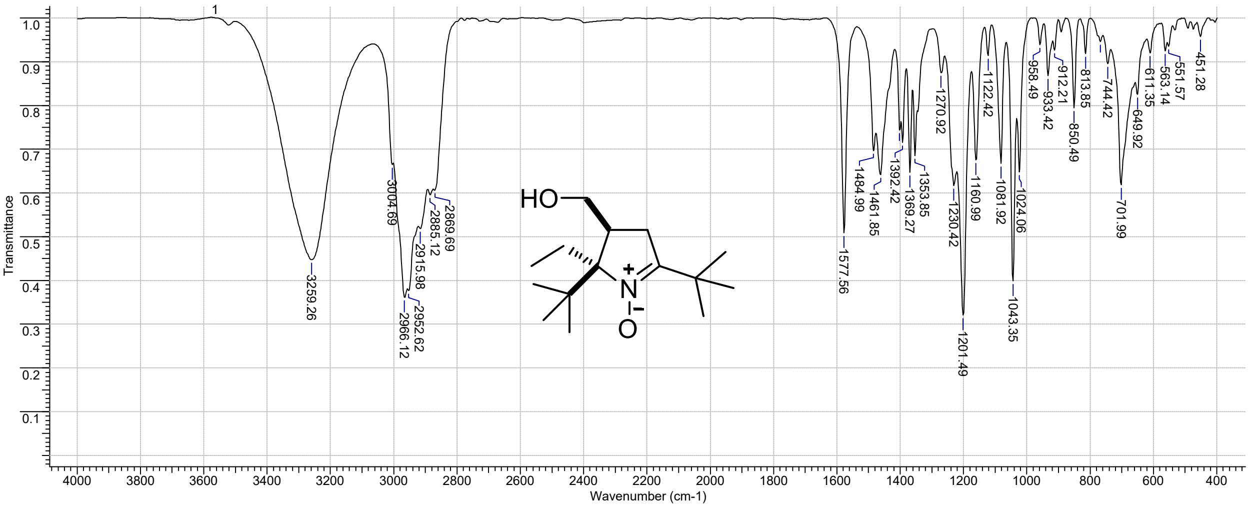

Figure S62. IR (KBr) of (2S(R),3S(R))-2,5-di-tert-butyl-2-ethyl-3-(hydroxymethyl)-3,4-dihydro-2H-pyrrole 1-oxide (**21**)

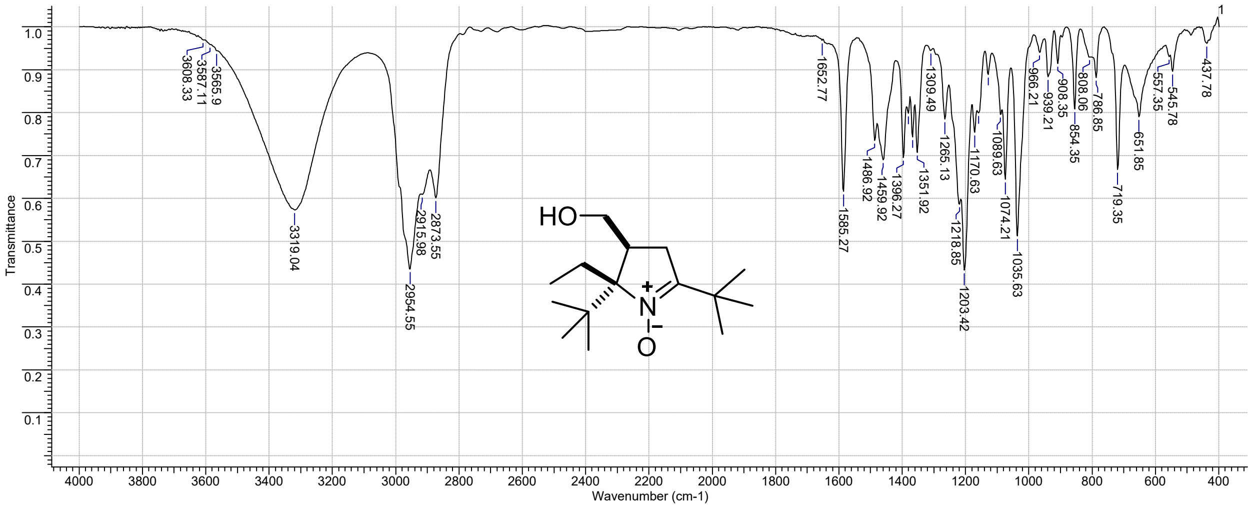

Figure S63. IR (neat) of (2S(R),3R(S))-2,5-di-tert-butyl-2,5-diethyl-3-(hydroxymethyl)pyrrolidin-1-oxyl (**23**)

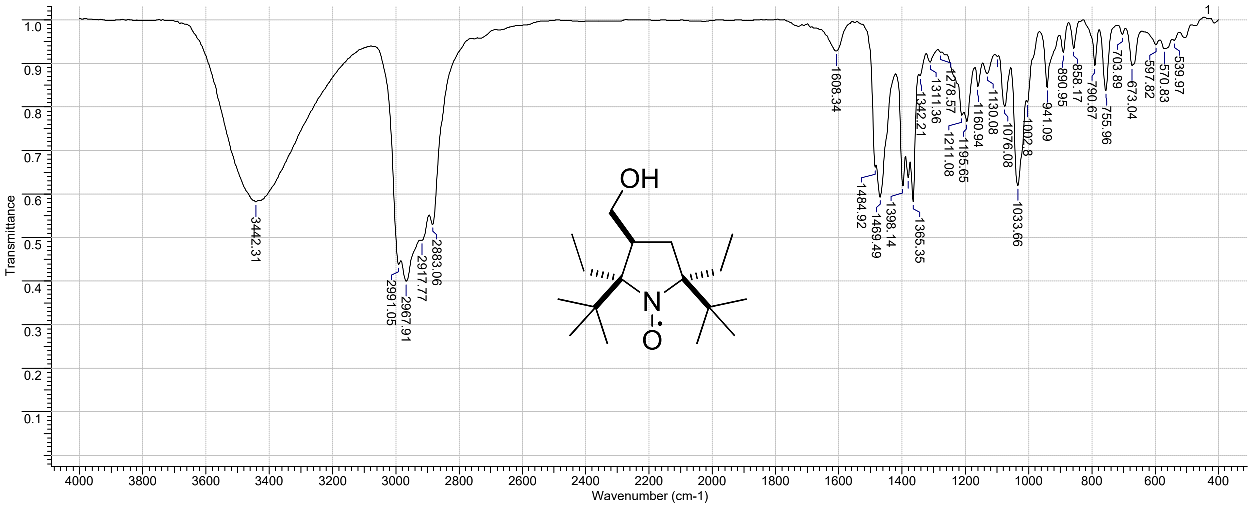

Figure S64. IR (neat) of (2S(R),3R(S),5S(R))-2,5-di-*tert*-butyl-3-((((3-(dimethylamino)propyl)carbamoyl)oxy)methyl)-2,5-diethylpyrrolidin-1-oxyl (**25**)

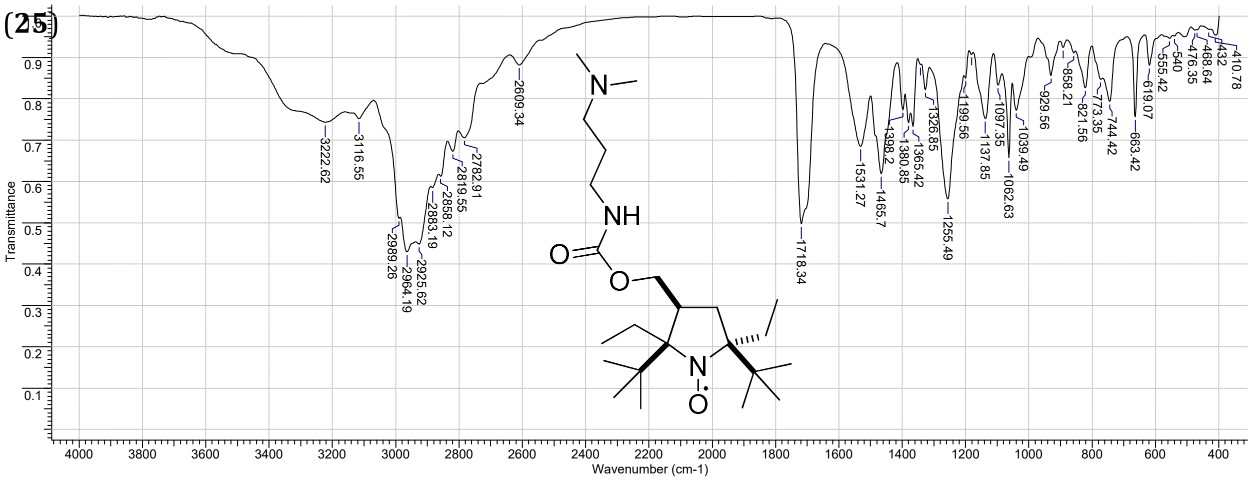

Figure S65. IR (neat) of mono((2S(R),3R(S),5S(R))-2,5-di-*tert*-butyl-2,5-diethyl-3-((((3-(trimethylammonio)propyl)carbamoyl)oxy)methyl)pyrrolidin-1-oxyl) monoiodide (**26**)

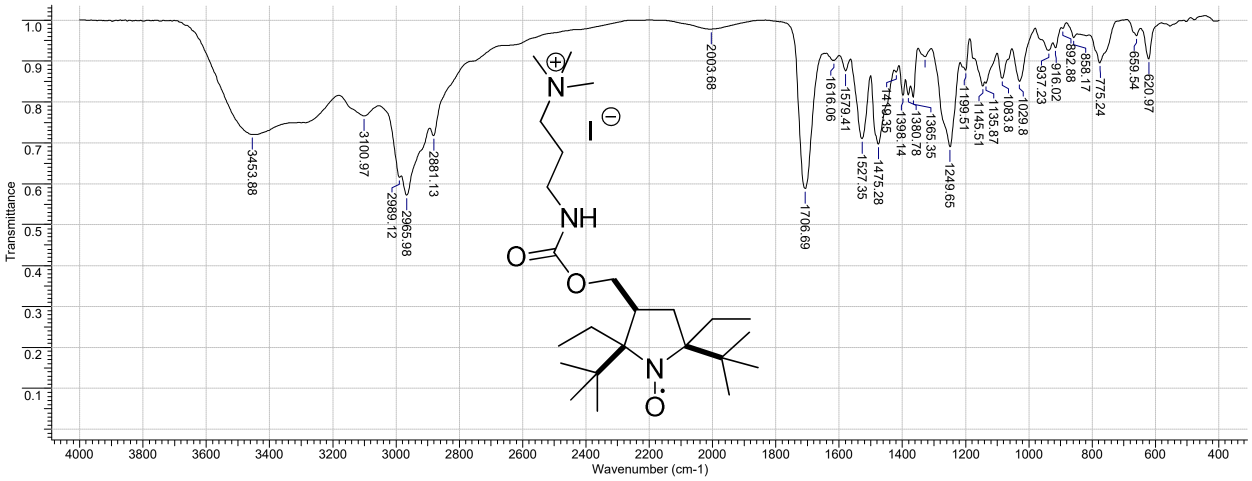

XRD Data

Chemical structures of the molecules **20** and **21** obtained by X-ray single crystal diffractometry:

Figure S66. Crystal structure for (2S(R),3R(S))-2,5-di-tert-butyl-2-ethyl-3-(hydroxymethyl)-3,4-dihydro-2H-pyrrole 1-oxide (**20**)

Figure S67. Crystal structure for (2S(R),3S(R))-2,5-di-tert-butyl-2-ethyl-3-(hydroxymethyl)-3,4-dihydro-2H-pyrrole 1-oxide (**21**)

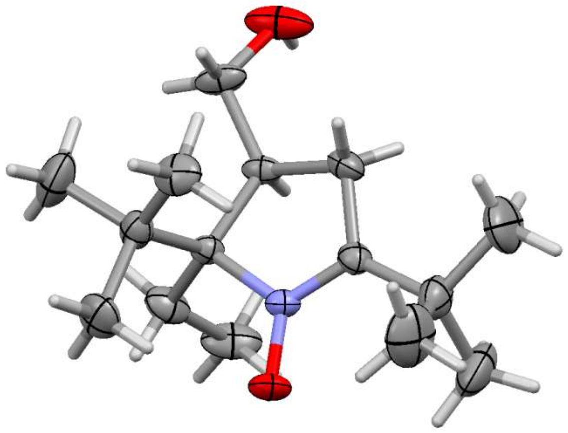

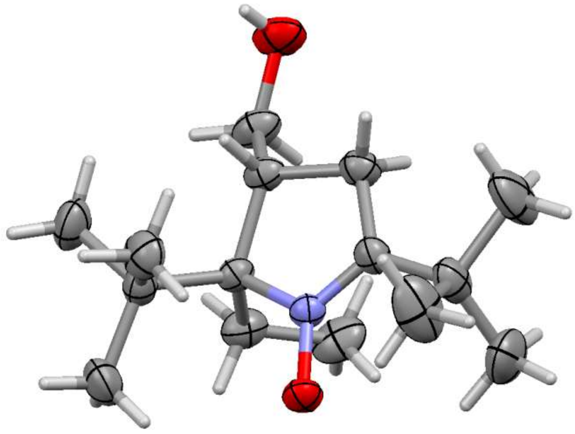

Crystal data **20**: $C_{15}H_{29}NO_2$, M = 255.39, monoclinic, space group C2/c, at 296 K: a = 20.1597(14), b = 15.1070(10), c = 12.4016(7) Å , β = 119.835(2)°, V = 3276.3(4) Å^3, Z = 8, d_{calc} = 1.036 g·cm^{-3}, μ = 0.067 mm^{-1}, a total of 26540 (θ_{max} = 28.02°), 3964 I unique (R_{int} = 0.0623), 2877 [I > 2σ(I)], 170 parameters. GooF = 1.001, R_1 = 0.0887, wR_2 = 0.2676 [I > 2σ(I)], R_1 = 0.1107, wR_2 = 0.3048 (all data), max/min diff. peak 1.39/-0.32 e·Å^{-3}. CCDC 2312570.

Crystal data **21**: $C_{15}H_{29}NO_2$, M = 255.39, triclinic, space group P-1, at 296 K: a = 9.5627(8), b = 12.9565(13), c = 13.4896(14) Å , α = 85.641(4), β = 88.100(4), γ = 79.040(3)°, V = 1635.8(3) Å^3 , Z = 4, d_{calc} = 1.037 g·cm $^{-3}$, μ = 0.067 mm^{-1}, a total of 16573I (θ_{max} = 25.21°), 5832 unique (R_{int} = 0.0642), 3298 [I > 2σ(I)], 339 parameters. GooF = 1.015, R_1 = 0.0616, wR_2 = 0.1775 [I > 2σ(I)], R_1 = 0.1144, wR_2 = 0.2142 (all data), max/min diff. peak 0.34/-0.18 e·Å^{-3}. CCDC 2312569.

EPR Data

Figure S68. Reduction profiles of **1a** (0.2 mM) in anaerobic conditions in phosphate-citrate-borate buffer (5 mM, pH 7.4) in presence of GSH (5 mM) and ascorbate (200 (,)■300●( )mM). The amplitude of the low-field component of the EPR spectrum was followed, spectrometer settings: frequency, 9.87 GHz; centerfield, 350.6 mT; sweep range, 10 mT; microwave power, 2.0 mW; modulation amplitude, 0.1 mT; time constant, 10.24 ms; and conversion time, 20.48 ms.

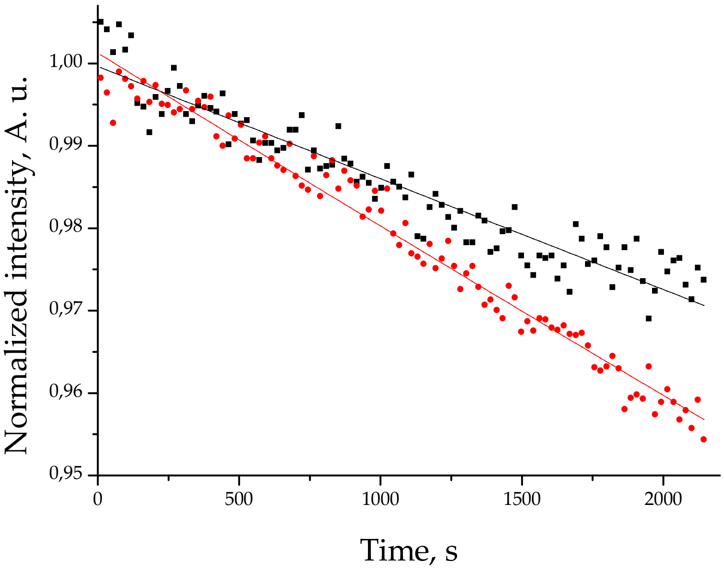

Figure S69. The plot of the nitroxide **1a** first order decay constant k_1 versus ascorbate concentration and the linear fitcorresponding to k_2 = (7.0 ±2×) 10^{-5} $M^{-1}s^{-1}$.

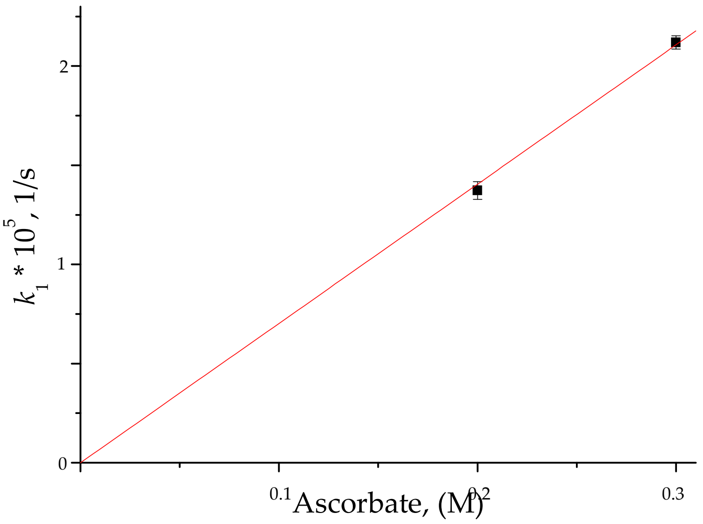

Figure S70. EPR spectrum of **26** (0.2 mM) in distilled water. Spectrometer settings: frequency, 9.87 GHz; microwave power, 5.0 mW; modulation amplitude, 0.05 mT; time constant, 50–100 ms; and conversion time, 5.12 ms.

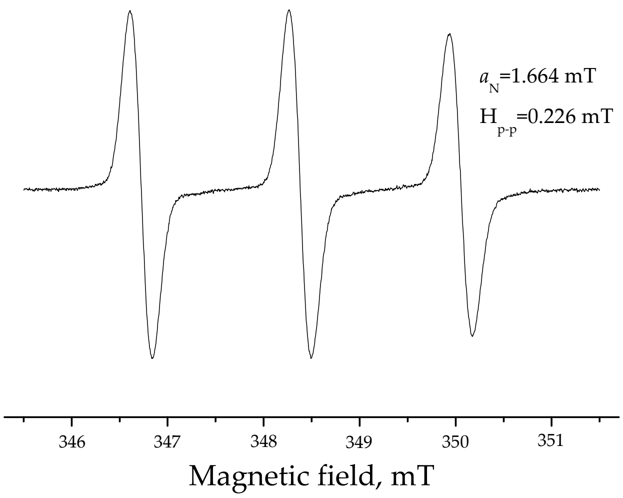

Figure S71. Reduction profiles of **26** (0.2 mM) in anaerobic conditions in phosphate-citrate-borate buffer (5 mM, pH 7.4) in presence of GSH (5 mM) and ascorbate (100 (,)■200●( ) and 300 (◆) mM). The amplitude of the low-field component of the EPR spectrum was followed, spectrometer settings: frequency, 9.87 GHz; centerfield, 350.6 mT; sweep range, 10 mT; microwave power, 2.0 mW; modulation amplitude, 0.1 mT; time constant, 10.24 ms; and conversion time, 20.48 ms.

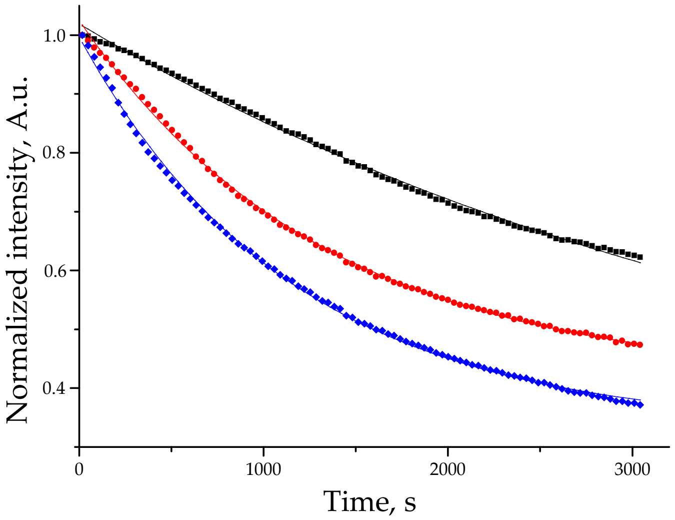

Figure S72. The plot of the nitroxide **26** first order decay constant k_1 versus ascorbate concentration and the linear fit corresponding to k_2 = (2.0 ±0.1×) 10^{-3} $M^{-1}s^{-1}$.

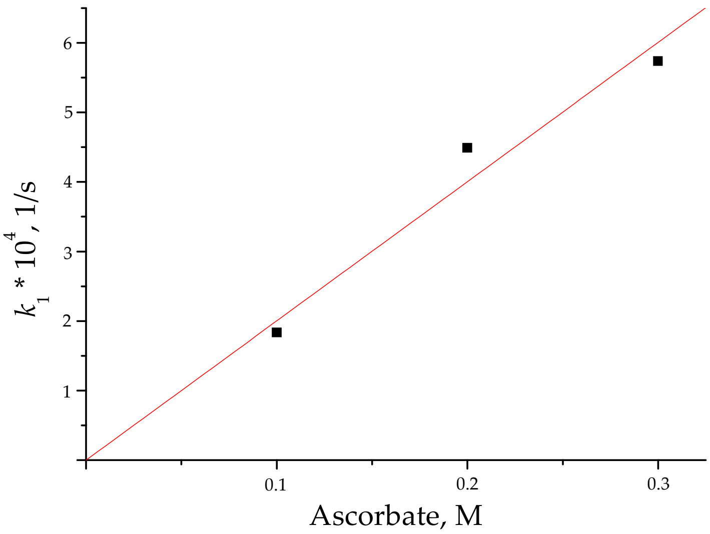

Supplement: Supplementary file 1 [file molecules-29-00599-s001.zip › molecules-2789571-supplementary.pdf]
